# High-resolution landfill characterization using SAR remote sensing and cloud-based processing

**DOI:** 10.1038/s41598-025-32908-9

**Published:** 2025-12-21

**Authors:** Shashank Agrawal, Shivukumar Rakkasagi, Manish Kumar Goyal

**Affiliations:** Department of Civil Engineering, Indian Institute of Technology Indore, Indore, India

**Keywords:** Sentinel-1 DEM generation, Remote sensing, Landfill statistics, Indian landfill sites, Landfill monitoring, Engineering, Environmental sciences, Environmental social sciences

## Abstract

Solid waste management in developing countries such as India faces persistent challenges due to weak monitoring systems and the absence of reliable reporting mechanisms for landfill statistics. To address this gap, this study develops a remote sensing methodology that integrates Python programming with the Sentinel Application Platform (SNAP) to generate Digital Elevation Models (DEMs) from Sentinel-1 synthetic aperture radar (SAR) imagery for quantifying landfill characteristics. Key parameters, including waste height and volumetric estimates, were extracted from satellite observations and processed through Google Earth Engine (GEE), enabling efficient large-scale analysis. A total of 80 landfill sites distributed across India were examined, providing the first nationwide assessment of landfill volume using a uniform and replicable framework. Field validation was conducted at two representative sites, Gondiya Landfill and Ujjain Ring Road Trenching Ground, through drone surveys and Differential Global Positioning System (DGPS) measurements. The evaluation showed deviations of 21.12% and 0.12% in height, 0.7% and 0.65% in area delineation, and 20.21% and 0.8% in volume for Gondiya and Ujjain, respectively, confirming the reliability of the proposed approach. These results demonstrate that SAR-based DEMs offer a cost-effective and scalable solution for systematic, near real-time monitoring of landfills across large regions. The framework not only supports capacity planning, environmental assessments, and policy formulation but also provides a pathway for developing countries to transition toward data-driven waste management strategies in the context of rapid urbanization and increasing waste generation.

**Supplementary Information:**

The online version contains supplementary material available at 10.1038/s41598-025-32908-9.

## Introduction

Global solid waste generation has experienced unprecedented growth, reaching approximately 2.1 billion tonnes in 2023, with projections indicating a surge to 3.8 billion tonnes by 2050 driven by rapid urbanization, population expansion, and changing consumption patterns^1,2^. This exponential increase poses significant environmental and public health challenges, particularly in developing nations where waste management infrastructure struggles to keep pace with generation rates^3^. India, as one of the world’s most populous countries, generates over 170,300 metric tons of municipal solid waste daily, with per capita waste generation ranging from 0.2 to 0.6 kg per day across urban areas^4,5^. The country’s landfills are predominantly characterized by unscientific disposal practices, with over 90% operating as open dumps without proper engineering controls, liner systems, or leachate management^6,7^. This inadequate landfill management has resulted in severe environmental degradation, groundwater contamination, and greenhouse gas emissions, with urbanization, industrialization, and unplanned urban expansion significantly disrupting waste generation dynamics^8^. The critical need for effective landfill monitoring systems has become paramount, particularly focusing on waste accumulation patterns, height variations, and volumetric assessments^9^. Accurate estimation of waste height and volume is essential for understanding landfill capacity utilization, predicting lifespan, planning waste diversion strategies, and ensuring compliance with environmental regulations^10,11^. Such monitoring enables authorities to make informed decisions regarding landfill expansion, closure planning, and implementation of waste reduction measures, ultimately contributing to sustainable waste management practices^12^.

Traditional landfill monitoring techniques have predominantly relied on ground-based surveying methods, including topographical surveys using total stations, GPS measurements, and manual volume calculations through geometric approximations^13–15^. These conventional approaches, while providing reasonable accuracy for localized assessments, are often time-consuming, labor-intensive, and costly, particularly for large-scale landfill sites^16,17^. Ground-based methods also pose safety risks to personnel due to unstable waste surfaces, hazardous gas emissions, and potential equipment damage from harsh landfill environments^18,19^. The spatial and temporal resolution of conventional monitoring is often inadequate for comprehensive landfill characterization, particularly for sites spanning several hectares with complex topography^20^. Recent studies have highlighted these limitations, emphasizing that traditional waste monitoring approaches in developing countries lack uniformity, objectivity, and wide coverage capabilities^21–23^. The methodology for measuring landfill dumping statistics globally using Digital Elevation Change maps has emerged as a significant advancement, offering systematic approaches to overcome these traditional limitations^24^.The inherent limitations of ground-based techniques have necessitated the exploration of remote sensing technologies that can offer synoptic coverage, temporal repeatability, and cost-effective monitoring solutions^25^. Remote sensing approaches provide the capability to monitor landfills from space-borne or airborne platforms, eliminating safety concerns while enabling regular assessment of waste height variations and volumetric changes across entire landfill sites with consistent spatial as well as temporal resolution^26–29^.

Remote sensing technologies like Synthetic Aperture Radar (SAR), particularly Sentinel-1 imagery from the European Space Agency’s Copernicus program, has become a modern tool for landfill monitoring applications due to its usefulness in generating high-resolution Digital Elevation Models (DEMs) through interferometric processing techniques^30,31^. Sentinel-1’s C-band radar operates with a temporal revisit frequency of 6–12 days, providing consistent data acquisition regardless of weather conditions or illumination constraints, making it ideal for continuous landfill monitoring^32–34^. The interferometric SAR (InSAR) processing of Sentinel-1 data enables the generation of DEMs suitable for assessing surface elevation changes over landfill areas, thereby facilitating volume calculation. Although InSAR-derived DEMs have demonstrated sub-meter vertical accuracy in controlled studies, the practical accuracy over landfill sites is influenced by site-specific surface conditions and data quality^35–37^. Recent research has demonstrated successful applications of SAR interferometry for monitoring ground subsidence at landfill sites, with studies utilizing persistent scatterer methods on Sentinel-1 data achieving high precision in deformation measurements^38,39^. Unlike optical remote sensing systems that are limited by cloud cover and atmospheric conditions, Sentinel-1’s active radar system penetrates atmospheric disturbances, ensuring reliable data acquisition throughout the year^40–42^. Advanced InSAR processing techniques have shown significant improvements in terrain feature-guided algorithms for DEM generation, with recent developments in 2024 demonstrating enhanced void-filling capabilities and improved accuracy^43^. Recent comprehensive surveys on solid waste detection and monitoring in remote sensing images have highlighted the growing importance of SAR optical data fusion for enhanced landfill characterization^17,25^.

This study addresses the critical gap in cost-effective, scientifically robust landfill monitoring methodologies by demonstrating the application of Sentinel-1 derived DEMs for estimation of landfill waste height and volume. The study contributes to the advancement of remote sensing applications in waste management, through analysis of 80 landfill sites across India, providing a replicable framework for landfill monitoring that can be implemented across diverse geographical and operational contexts. This research demonstrates the methodology robustness and applicability for estimating landfill height and volume parameters, providing valuable insights for waste management assessment and monitoring.

## Methodology

**Fig. 1:**
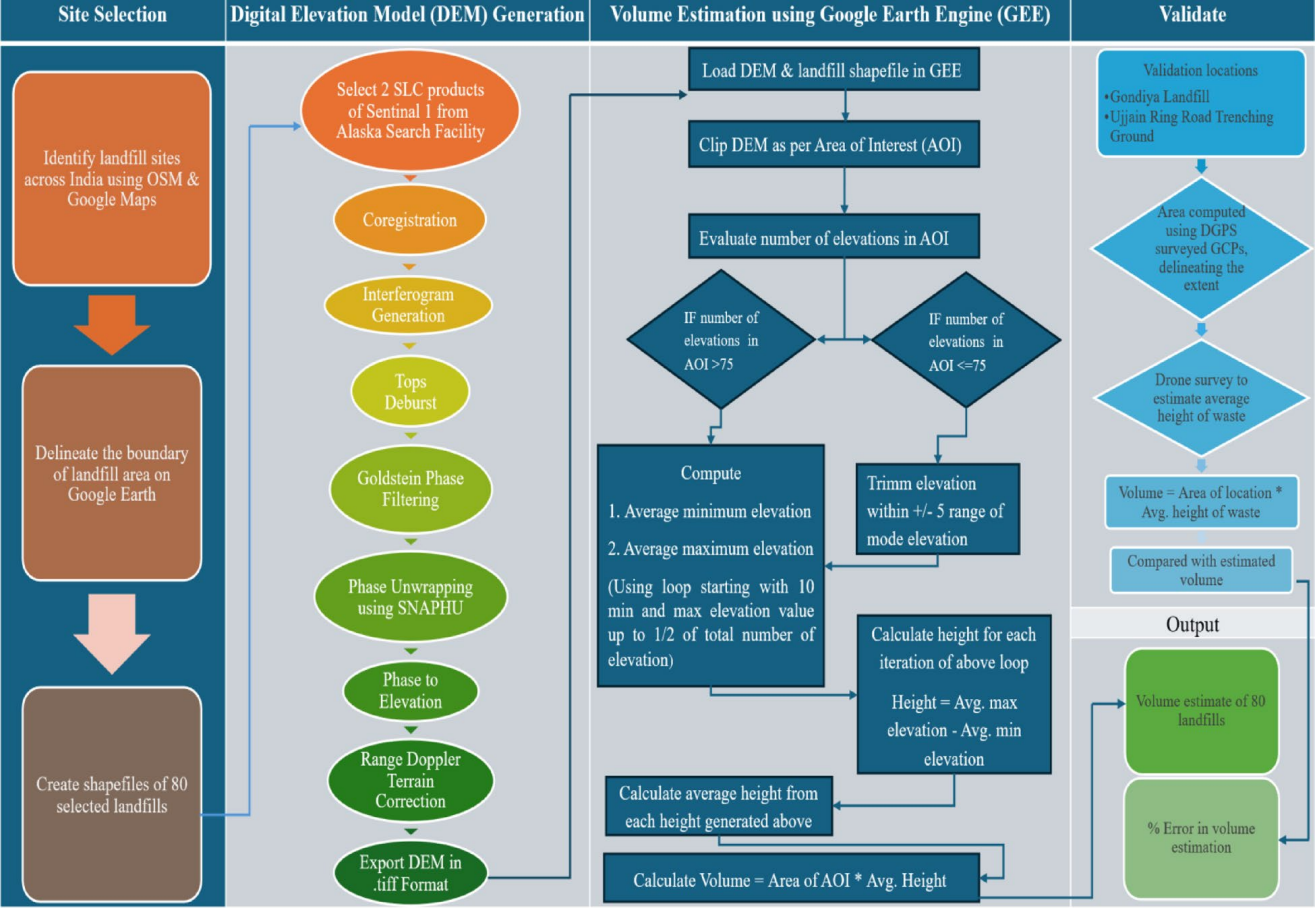
Schematic representation of the proposed study methodology, illustrating the sequential steps and data processing workflow. This figure was created using Microsoft PowerPoint Presentation 365, Version 2510 (https://www.microsoft.com/en-in/microsoft-365/powerpoint).

The study encompassed 80 selected landfill sites throughout India, analyzed during January 2025 to determine average height and solid waste volume parameters for each location. Validation of the proposed analytical approach was achieved through detailed field surveys conducted at two representative case study sites. Figure 1 provides a schematic representation of the complete methodological framework.

### Landfill site selection

**Fig. 2:**
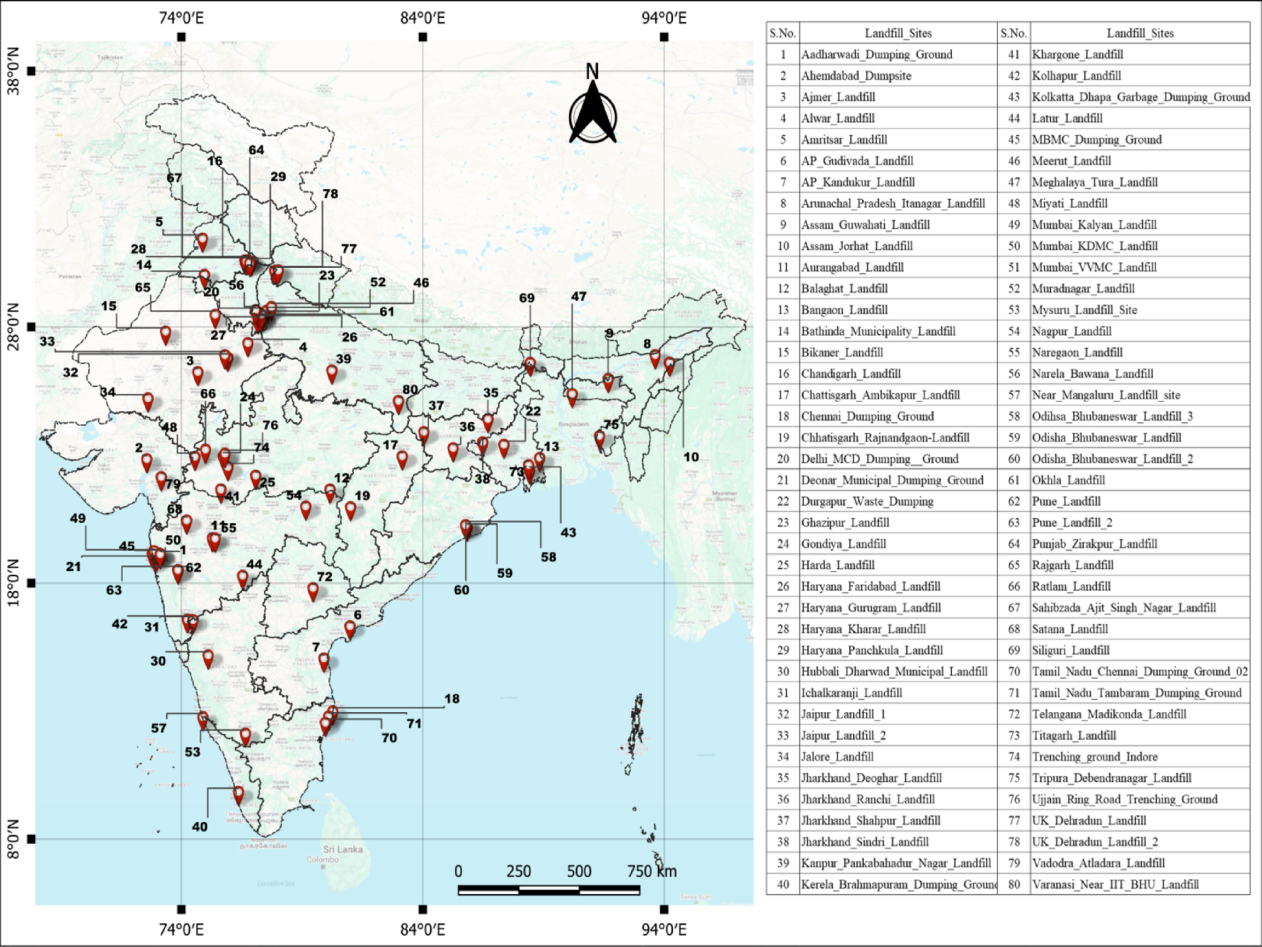
Study area map displaying 80 landfill locations distributed throughout India, represented by red placemark symbols to show the comprehensive geographic coverage of the research sites. This figure was created using QGIS 3.40.3 (Quantum Geographic Information System; https://download.qgis.org/downloads/) and the background shows the base-map of Google Maps from HCMGIS Plugin. The shape file of State boundary outline map of India is obtained from the Survey of India’s official Free Other Maps repository (https://onlinemaps.surveyofindia.gov.in/FreeOtherMaps.aspx).

The primary considerations for selecting case-study sites were to ensure comprehensive coverage across India’s diverse geographical and administrative regions, demonstrating the methodology’s applicability across varied Indian contexts. Open dumpsites were included to represent the heterogeneous waste management landscape in India. The initial identification of potential landfill sites was conducted using OpenStreetMap (OSM) data^44^ accessed through the OSMnx Python library^45^ in Google Colab environment. Indian states were systematically queried to identify features tagged with “landuse”: “landfill” and “amenity”: “waste_disposal” to capture existing waste disposal facilities and designated landfill areas. While OSM provides comprehensive crowdsourced geographic data, certain limitations were acknowledged, including potential regional variations in data completeness and classification consistency among contributors, particularly in rural areas where mapping coverage may be less comprehensive than urban regions^46,47^. However, ground-truthing through Google Earth revealed that many OSM tagged locations did not correspond to actual operational or identifiable landfill sites, with no visible waste accumulation or landfill infrastructure present. To address this Google Maps was subsequently employed as a supplementary data source, using targeted keyword searches including “dumping grounds,” “landfill,” and “solid waste management” to identify additional sites. This multisource approach, combining OSM automated tagging, satellite imagery verification, and manual search methods, ultimately resulted in the selection of 80 verified landfill sites across India. The final dataset shown in Fig. 2 encompasses sites of varying sizes, ranging from 10,000 sq m to 1,000,000 sq m, providing a representative sample of India’s landfill infrastructure for methodological validation and regional analysis. Satellite Imageries and other details for each site were provided in Appendix B.

### Description of data used

The dataset utilized in this study consists of Sentinel-1 Single Look Complex (SLC) products acquired in January 2025 and downloaded from the Alaska Satellite Facility (ASF) Data Search platform^48^. These C-band synthetic aperture radar (SAR) data products provide the foundation for digital elevation model (DEM) generation through interferometric processing techniques. To enable spatial analysis focused specifically on landfill areas, boundary shapefiles for each of the landfill sites were manually delineated using Google Earth 2025 imagery^49^ as the reference base, with coordinates recorded in the WGS84 geographic coordinate system (EPSG:4326). This approach ensures spatial definition of the landfill boundaries, accounting for the irregular geometries typical of waste disposal sites and enabling accurate extraction of elevation data within the areas of interest. The high-resolution optical imagery available through Google Earth provides sufficient detail to distinguish landfill boundaries from surrounding terrain and infrastructure, facilitating accurate digitization of site perimeters^50^. The combination of radar derived elevation data and optically defined spatial boundaries creates a robust dataset for analyzing elevation characteristics and potential changes within the designated landfill areas across the study region.

### Generation of digital elevation models (DEMs)

Digital elevation models for the study areas were generated using Sentinel-1 Single Look Complex (SLC) products obtained from the Alaska Satellite Facility (ASF) Data Search platform^48^. The digital elevation model (DEM) was generated through interferometric processing of Sentinel-1 Single Look Complex (SLC) data using a Python-based workflow integrated with the European Space Agency’s Sentinel Application Platform (SNAP) software^51^. The DEM generation process involved a few key processing steps such as coregistration of interferometric pairs of SLC images to ensure precise geometric alignment, followed by interferogram generation to calculate the phase difference between radar signals and coherence estimation to assess data quality. Afterwards phase unwrapping was executed to convert wrapped phase values into continuous elevation measurements, with topographic phase removal applied to isolate terrain related phase components. Finally, geocoding and radiometric calibration were performed to transform the radar geometry data into georeferenced elevation products suitable for spatial analysis^31,52–55^. The procedure of DEM generation is described in more detail in Appendix A. This interferometric processing chain produces high-resolution DEMs with a spatial resolution of approximately 10 m that accurately represent the topographic characteristics of the selected landfill sites across the study region^56^. The DEMs generated offer several key advantages, including all weather acquisition capability independent of cloud cover, consistent temporal coverage enabling change detection analysis, and the ability to penetrate vegetation canopy to some extent, providing more accurate ground surface representation^57,58^. The 10 m DEMs generated in this study were suitable for estimating average height characteristics across landfill areas of varying sizes, providing sufficient spatial detail to capture elevation variations and waste pile distributions in larger sites. This resolution enables reliable height estimation for landfills of all sizes within the study dataset, facilitating comparative analysis across sites with different spatial extents and waste management practices. Elevations profile for each landfill site is available in Appendix B.

### Volume Estimation of landfill sites

Volume estimation for each landfill site was conducted using the Google Earth Engine (GEE) platform through a comprehensive statistical approach that processes elevation data within the defined area of interest (AOI)^59–62^. The methodology begins by loading the generated DEM raster and corresponding landfill boundary shapefile as assets within the GEE environment. The DEM is subsequently clipped to the precise AOI geometry to ensure that elevation analysis is confined strictly to the landfill boundaries. First, all elevation values within the AOI are extracted, creating an extensive dataset of pixel-level elevation measurements. These elevation values are then sorted in ascending order to facilitate statistical analysis and determine the appropriate computational approach based on sample size.

The methodology employs an adaptive processing strategy that selects the appropriate computational approach based on the total number of elevation values (N) within each AOI. This dual-approach framework ensures optimal processing efficiency while maintaining computational accuracy across landfill sites of varying sizes.

- **For AOIs with N ≤ 75 elevation values**, the algorithm implements a statistical mode filtering approach to enhance data robustness. The modal elevation value serves as a statistical reference baseline for identifying anomalous elevation measurements that exceed acceptable deviation thresholds. The elevation trimming algorithm implements a conditional replacement function mathematically expressed as:
  1 $$\begin{aligned} \rm{E{^\prime}_i }= &\{ \\&\rm{Mode(E) \ if \ |E_i - Mode(E)|> \delta} \\& \rm{ E_i \quad otherwise} \\&\}  \end{aligned}$$

where E’_i_ represents the filtered elevation value, E_i_ denotes the original pixel elevation, Mode(E) is the statistical mode of the elevation distribution, and δ = 5 m defines the outlier threshold parameter.

- **For AOIs with N > 75 elevation values**, the algorithm bypasses the trimming process and directly processes the sorted elevation dataset to maintain computational efficiency for larger datasets. This approach leverages the statistical robustness inherent in larger sample sizes, where outlier effects are naturally minimized through increased data density.
- **Height Difference Computation**.

**Fig. 3:**
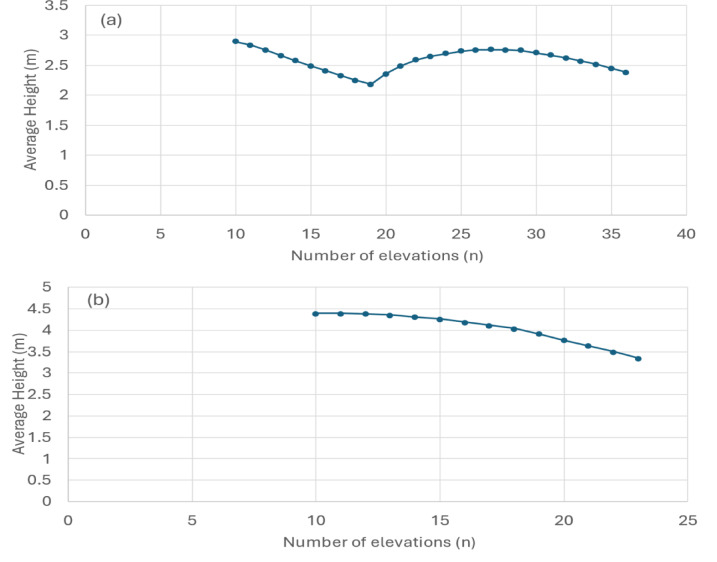
Average height (m) as a function of number of elevation pixel population (n) for two landfill sites: (**a**) Gondiya landfill and (**b**) Ujjain Ring Road Trenching Ground. This figure was created using Microsoft Excel 365, Version 2510 (https://www.microsoft.com/en-in/microsoft-365/excel).

Both processing pathways employ an iterative statistical sampling approach that computes elevation differentials across variable sample sizes (n ∈ [10, N/2]), where N represents the total pixel population within the AOI. This methodology calculates the arithmetic mean of the n-lowest and n-highest elevation subsets to derive representative baseline and peak elevation values, respectively as shown in Fig. 3(a) and 3(b) for the two landfill sites used for validation.

2 $$\:{H}_{GEE}\:=\:\left(\frac{1}{k}\right)\times\:\:\varSigma_{j}^{=1k}\:\left[{\bar E}^{\prime}_{\rm max}\left(n_j \right)- \:{\bar E}^{\prime}_{\rm min} \left(n_j\right)\right]$$

where ${\bar E}^{\prime}_{\rm min} \left(n\right)\:=\:(1/n)\:\times\:\:\varSigma_{i=1}^n \:E^{\prime}_{(i)}$(arithmetic mean of n-lowest ranked elevation values), ${\bar E}^{\prime}_{\rm max} \left(n\right)\:=\:(1/n)\:\times\:\:\varSigma_{i=1(n -n+1)}^N\: E^{\prime}_{(i)}$(arithmetic mean of n-highest ranked elevation values), k represents the total number of iterations across varying sample sizes, E’₍_i_₎ denotes the i^th^ ranked elevation value in ascending order, and $\:{\bar H}_{GEE}$ represents the estimated average height through Google Earth Engine.

- **Volume Calculation**

The volumetric estimation was computed consistently using the standardized formula:

3 $$\:{V\:}_{GEE}=\:{H}_{GEE}\:\times\:\:{A}_{GEE}\:$$

where $\:{V\:}_{GEE}$ represents the estimated landfill volume (m^3^), $\:{\bar H}_{GEE}\:$denotes the average height (m), and $\:{A}_{GEE}$ represents the planimetric area of the AOI (m^2^) derived from geometric polygon calculations using GEE.

This adaptive GEE-based approach enables efficient processing of heterogeneous datasets while maintaining computational accuracy and providing standardized volume estimates across all 80 landfill sites in the study, regardless of their spatial extent or pixel density. The methodology also computes essential statistical parameters like minimum and maximum elevations, mode elevation (where applicable), and total AOI area to estimate the required output of landfill waste volume and average height of waste in landfills for each landfill site.

### On-site survey-based validation of two landfill sites

**Fig. 4:**
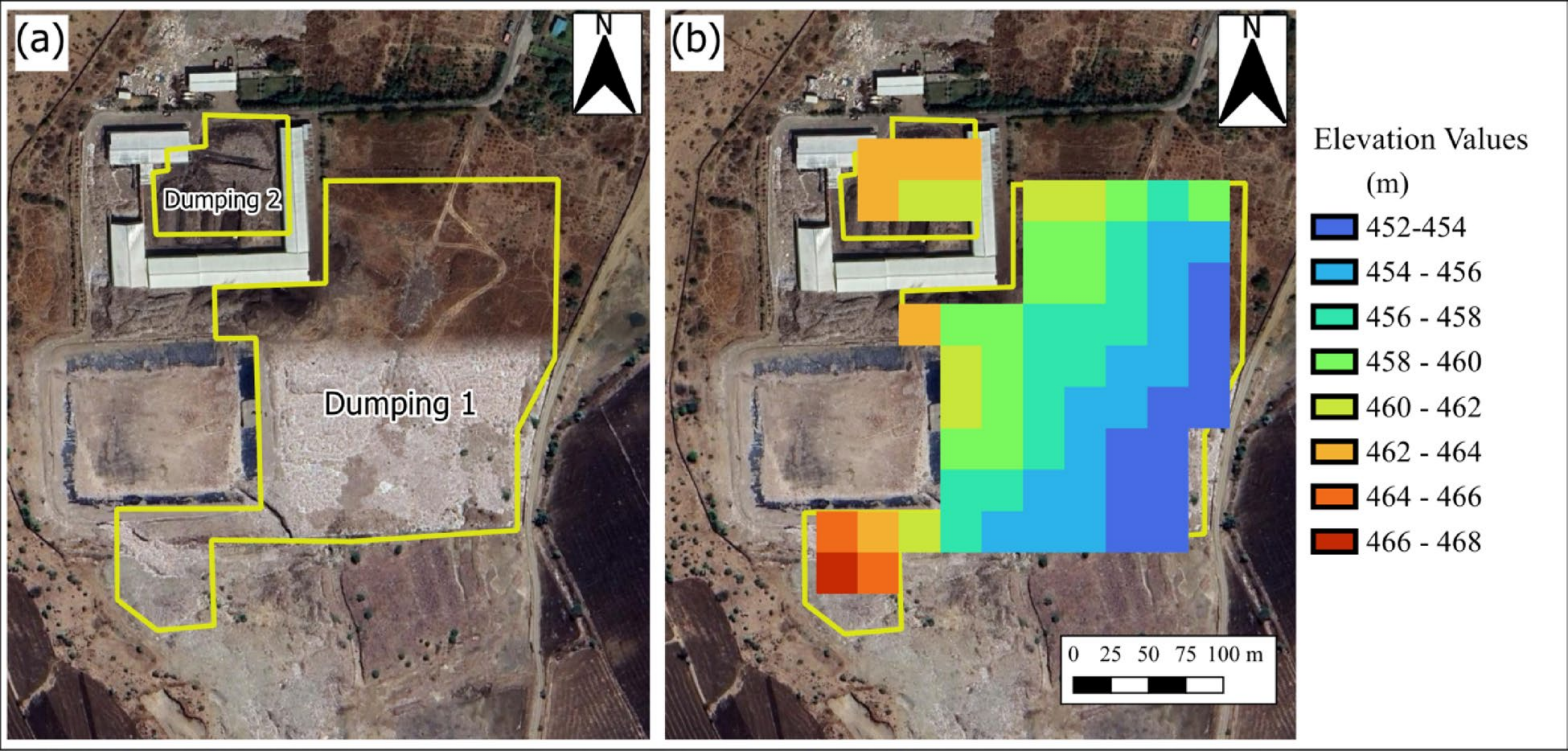
Spatial representation of the Gondiya landfill in India, showing (**a**) Google Earth imagery with active waste zones outlined in yellow, and (**b**) Landfill elevation variations from Sentinel-1 DEM data. This figure was created using QGIS 3.40.3 (Quantum Geographic Information System; https://download.qgis.org/downloads/) and the background shows the base-map of Google Satellite from HCMGIS Plugin.

**Fig. 5:**
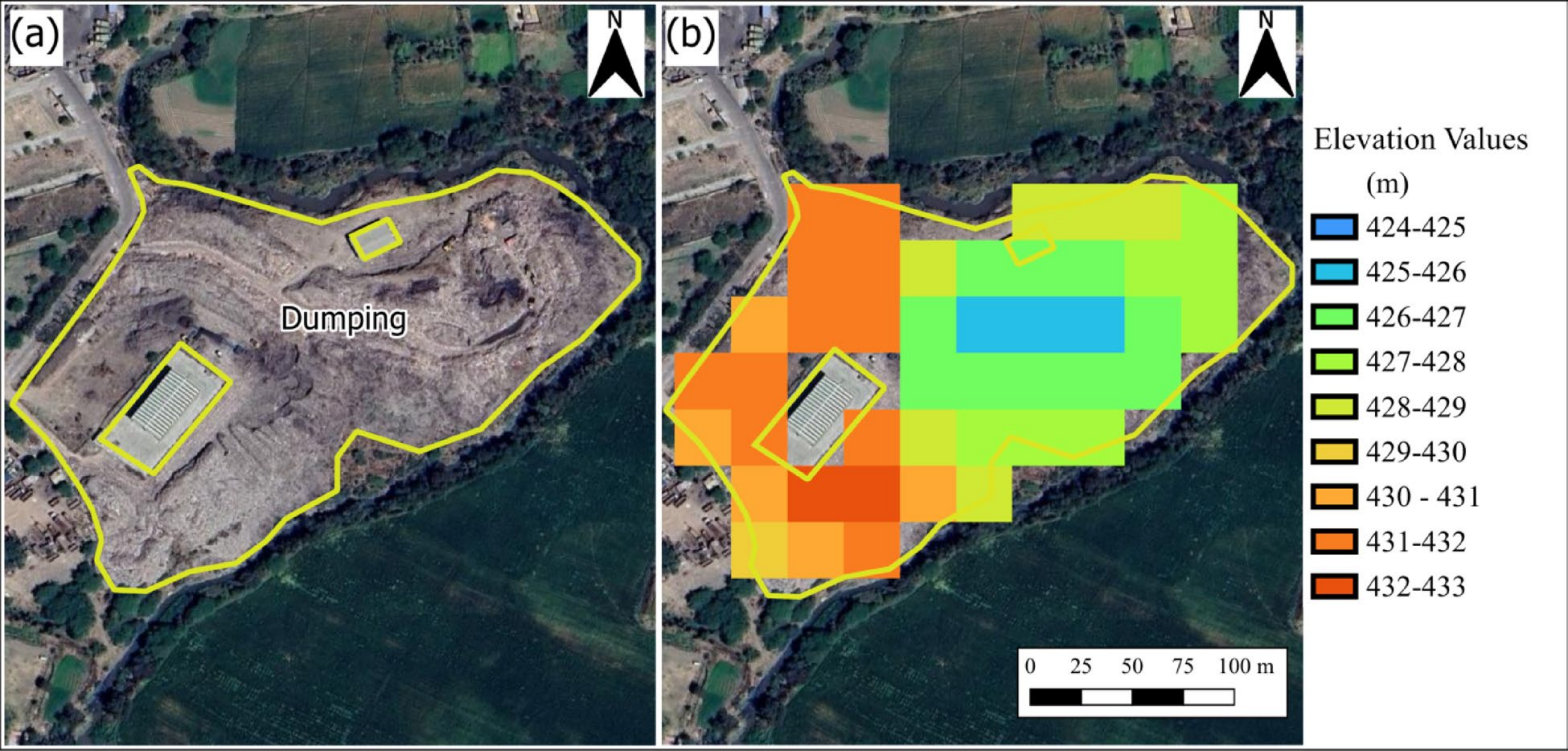
Spatial representation of the Ujjain Ring Road Trenching Ground landfill in India, showing (**a**) Google Earth imagery with active waste zones outlined in yellow, and (**b**) Landfill elevation variations from Sentinel-1 DEM data. This figure was created using QGIS 3.40.3 (Quantum Geographic Information System; https://download.qgis.org/downloads/) and the background shows the base-map of Google Satellite from HCMGIS Plugin.

To assess the accuracy of the proposed remote sensing-based volume estimation methodology, a field validation study was conducted using two representative landfill sites: Gondiya Landfill (Location: 23.0900564°N, 75.7416676°E; Area: 59,966.81 m^2^) shown in Fig. 4 and Ujjain Ring Road Trenching Ground (Location: 23.193452574°N, 75.805920405°E; Area: 39,302.804 m^2^) shown in Fig. 5. The field validation employed Differential Global Positioning System (DGPS) to establish Ground Control Points (GCPs) for accurate mapping of landfill boundaries and topography, while detailed elevation measurements were conducted using Total Station to establish elevation points across each survey area. The site survey details are documented in Appendix A. Field-based volume estimation followed a systematic approach where height differential was calculated from arithmetic mean elevations at waste pile peaks and open ground level, and volume was estimated by multiplying the height differential by the AOI area. The validation analysis involved direct comparison between remote sensing-derived estimates and field measurements through percentage error calculations for both height differential and volume estimates. This validation framework provides a quantitative assessment of the proposed methodology’s accuracy across different landfill sizes and establishes confidence in the volume estimation approach for the broader 80-site study. The field-based volume estimation followed a systematic approach:

- **Field Height Calculation**

The on-site height of waste at landfill sites were estimated by using the equation:

4 $$\:{H}_{field}\:=\:{H}_{top}\:-\:{H}_{ground}\:$$

where $\:{\bar H}_{top}$ and $\:{\bar H}_{ground}$ represents the average elevations at waste pile peaks and open ground level, respectively (m), and $\:{H}_{field}$ represents the average height of waste (m).

- **Field Volume Estimation**

The volume of waste at field was estimated using the formula:

5 $$\:{V}_{field}=\:{H}_{field}\times\:\:{A}_{field}$$

where $\:{H}_{field}$ represents the average height of waste (m), $\:{A}_{field}\:$ represents the planimetric area of the AOI (m^2^) derived from the field survey.

- **Validation Parameters**

The validation analysis involved a direct comparison between remote sensing derived estimates and field measurements:

- **Height Estimation Accuracy**: Computation of percentage error between GEE derived and field-measured height differences.

6 $$\:\%\:Erro{r}_{height}=\:\left(\frac{\left({H}_{GEE}\:-\:{H}_{field}\right)}{{H}_{field}}\right)\times\:\:100$$

- **Area Delineation Accuracy**: Assessment of errors in delineating the boundary of the landfill using Google Earth and field survey.

7 $$\:\%\:Erro{r}_{area}=\:\left(\frac{\left({A\:}_{GEE}-{A}_{field}\right)}{{A}_{field}}\right)\times\:\:100$$

- **Volume Estimation Accuracy**: Assessment of volumetric estimates using percentage error calculations.

8 $$\:\%\:Erro{r}_{volume}=\:\left(\frac{\left({V\:}_{GEE}-{V}_{field}\right)}{{V}_{field}}\right)\times\:\:100\:$$

This validation framework provides a quantitative assessment of the proposed methodology’s accuracy across different landfill sizes and establishes confidence in the volume estimation approach for the broader 80-site study.

## Results

### Height and volume estimation

**Fig. 6:**
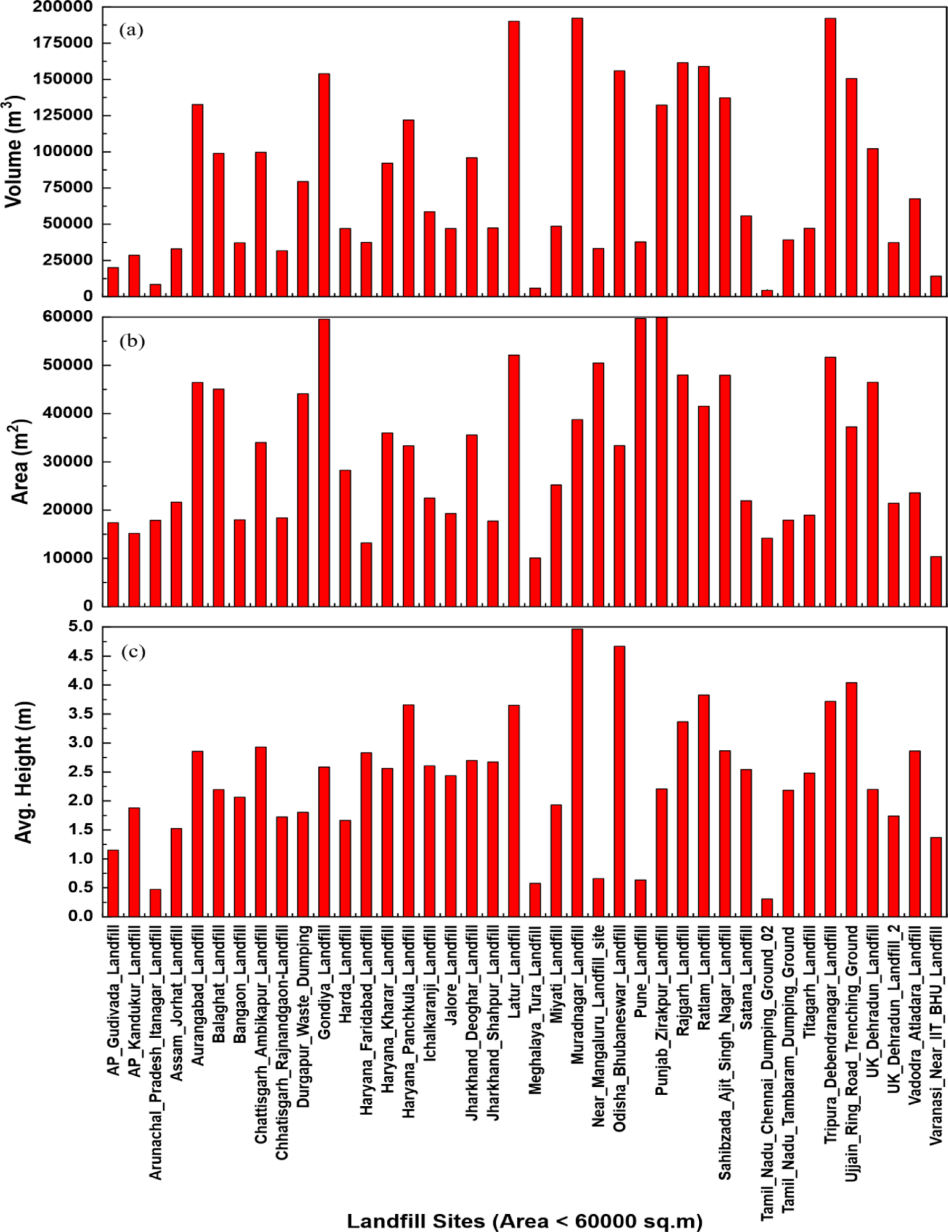
Graph showing comparative analysis of Indian landfill sites with area < 60,000 m^2^, illustrating (**a**) volume (m^3^), (**b**) area (m^2^), and (**c**) average height (m), arranged alphabetically by location. This figure was created using OriginPro 2025 (Learning Edition; https://www.originlab.com/OriginProLearning.aspx).

**Fig. 7:**
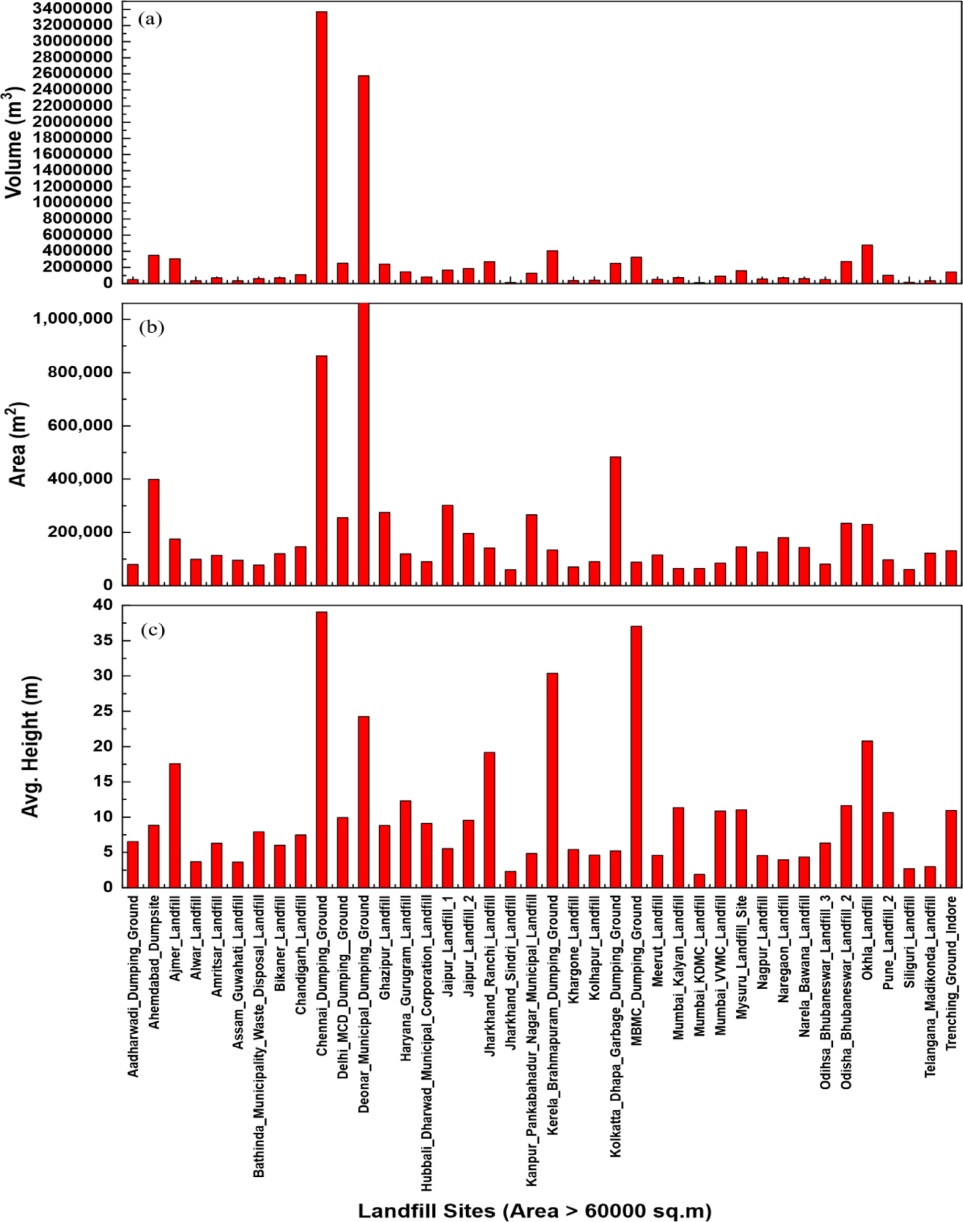
Graph showing comparative analysis of Indian landfill sites with area > 60,000 m^2^, illustrating (**a**) volume (m^3^), (**b**) area (m^2^), and (**c**) average height (m), arranged alphabetically by location. This figure was created using OriginPro 2025 (Learning Edition; https://www.originlab.com/OriginProLearning.aspx).

Height estimation was successfully conducted for all 80 landfill sites using elevation data analysis, with sites categorized based on a threshold of 75 elevation values to ensure data quality. For landfill sites with ≤ 75 elevation points, a trimming process was implemented that removed elevation values deviating beyond ± 5 m from the mode elevation, while sites with > 75 elevation points required no such processing due to sufficient data density. The height estimated using Eq. (1) revealed distinct patterns based on landfill area, where sites below 60,000 m² demonstrated maximum waste heights of 4.96 m, indicating appropriate waste management practices relative to available land area, whereas landfill sites exceeding 60,000 m² exhibited considerably greater waste heights from 2.71 m to 39 m primarily due to legacy waste accumulation over extended operational periods as shown in Fig. 6(c) and Fig. 7(c). Volume calculations were performed using Eq. (2), incorporating both area and height parameters, with results shown in Fig. 6(a) and Fig. 7(a) demonstrating the variation in waste accumulation height across different landfill areas. As per the study, the Chennai Dumping Ground in Chennai and Deonar Municipal Dumping Ground in Mumbai exhibited the largest waste accumulations, with volumes of 33,684,541.94 and 25,765,470.45 cubic meters respectively, reflecting their roles as major municipal waste disposal facilities serving large urban populations. The analysis confirmed that volume estimation is critically dependent on both geometric factors like correct area delineation of waste and height determination, with larger sites generally corresponding to higher volumes, though height variability attributed to waste compaction processes, disposal chronology, and accumulated legacy materials significantly impacts volumetric computations.

### Percentage error estimation

To validate the accuracy and reliability of the proposed methodology employed for landfill volume assessment, a comprehensive ground validation study was conducted at two representative landfill sites: Gondiya Landfill and Ujjain Ring Road Trenching Ground. These sites were selected to provide independent verification of the remote sensing-based approach through direct field measurements using established surveying techniques. The validation process involved a multi instrument approach combining Differential Global Positioning System (DGPS) technology with unmanned aerial vehicle (Drone) surveys to precisely delineate the landfill boundaries and establish accurate ground control points for area estimation. The DGPS system used for boundary mapping, while the drone survey enabled comprehensive aerial coverage and photogrammetric analysis of the landfill extent. Height measurements were obtained using a total station, a high precision surveying instrument capable of measuring both horizontal and vertical distances with millimeter accuracy. The field height measurement using Eq. (3) required two steps: first, measuring the average ground elevation at multiple reference points around the landfill boundary to establish the baseline level, and second, systematically measuring the average elevation of the waste surface to determine the height difference. This approach ensured that the height estimation accounted for natural ground variations and provided a representative measure of actual waste accumulation height. The field volumetric computation utilizing Eq. (4) was subsequently executed through the mathematical product of the derived mean height parameter and the delineated landfill areal extent, yielding the aggregate volumetric magnitude of waste deposition at each surveyed location. This approach assumes uniform waste distribution across the landfill area and provides a simplified volumetric estimate suitable for comparison with remote sensing derived calculations. The field-measured height and volume data for both validation sites were compared with the corresponding values derived from the remote sensing methodology, with results comprehensively tabulated in Table 1. Percentage error calculations were performed using established error estimation Eqs. (5), (6), (7) providing quantitative measures of the accuracy and precision of the remote sensing approach. The validation assessment yielded percentage deviations of 21.12% and 0.12% for height parameters, 0.7% and 0.65% for spatial area extraction, and 20.21% and 0.8% for volumetric computations at Gondiya Landfill and Ujjain Ring Road Trenching Ground respectively.

**Table 1:** Comparison of survey data versus estimated data for Gondiya landfill and Ujjain ring road trenching Ground, showing average height, area, and volume with corresponding percentage errors between actual survey values and computational estimates.

Landfill Sites	Gondiya Landfill			Ujjain Ring Road Trenching Ground		
	Survey Data	Estimated Data	**% Error from Survey Data**	Survey Data	Estimated Data	**% Error from Survey Data**
Average Height (m)	2.13	2.58	**21.12**	4.047	4.042	**0.12**
Area (sq. m)	59966.81	59541.53	**0.7**	39302.80	39044.22	**0.65**
Volume (cu. m)	127783.48	153937.30	**20.21**	159096.29	157816.73	**0.8**

## Discussion

The methodology proposed in this study represents a significant advancement in remote sensing applications for landfill management, offering a comprehensive approach to estimate landfill volumes across extensive geographical areas using Sentinel-1 synthetic aperture radar (SAR) imagery. As demonstrated in the study area (Fig. 2), the 80 landfill sites distributed across India provides important statistics in waste management practices across different regions and urban development levels.

This remote sensing-based approach covers the critical gaps in current waste management practices by providing a standardized, cost-effective method for volume estimation that eliminates the need for expensive and time-consuming on-site surveys^17^. The utilization of Sentinel-1 data with its all-weather imaging capability, consistent temporal resolution, and global coverage, making it particularly suitable for systematic monitoring of landfill sites represented in the study area^63^. The methodology presented was scalable from regional to global applications as of the global coverage of Sentinel-1 data offers unique opportunities for environmental monitoring agencies, municipal authorities, and waste management organizations to implement uniform assessment protocols across their operational domains^64,65^. Furthermore, the temporal dimension of Sentinel-1 data acquisition enables the expansion of this methodology to track volumetric changes over time^25^, providing insights into waste accumulation rates^66^ and long-term trends that are essential for strategic planning and policy formulation. This temporal analysis capability is particularly valuable for legacy landfill sites where historical records may be incomplete or unreliable, enabling authorities to better understand the evolution of waste disposal practices and their environmental implications.

The accuracy and reliability of the proposed methodology are fundamentally dependent on several critical technical factors, with Digital Elevation Model (DEM) quality serving as the keystone of accurate volume calculations. As illustrated in the results (Figs. 6 and 7), the relationship between landfill area and height demonstrates clear patterns that validate the methodology’s effectiveness, while also highlighting the importance of precise measurements for accurate volume estimations. The precision of DEMs derived from Sentinel-1 interferometric processing directly influences the accuracy of height measurements^31,67–69^, which in turn affects volume estimates through the multiplicative relationship between area and height parameters. Atmospheric corrections, phase unwrapping algorithms, and temporal decorrelation effects significantly impact DEM quality, necessitating robust processing workflows that incorporate multiple image pairs and advanced filtering techniques to minimize noise and artifacts^35,54,70^. The geometric accuracy of DEMs is particularly crucial in landfill environments where waste surfaces may exhibit irregular topography, steep slopes, and heterogeneous material properties that can introduce systematic errors in radar backscatter measurements.

Additionally, the delineation of active dumping areas represents another critical factor affecting methodology accuracy, as precise boundary identification is essential for accurate area calculations and subsequent volume estimations^71^. Manual digitization approaches, while providing high accuracy, are labor-intensive and subjective, whereas automated classification methods may struggle with the spectral similarity between waste materials and surrounding terrain features^72^. The integration of multi-temporal analysis and machine learning algorithms for boundary delineation shows promise for improving both accuracy and consistency in area estimation^73^. The accuracy of landfill boundary delineation critically affects volume estimation reliability, as manual digitization approaches introduce significant operational constraints that limit practical scalability^74^. Machine learning alternatives show technical promise, particularly semantic segmentation networks achieving 76–85% accuracy for waste facility detection in optical imagery^75,76^, SAR specific automated boundary detection remains predominantly at experimental implementation levels^77^. Consequently, operational SAR interferometry volume monitoring continues to rely heavily on manual approaches, constraining broader adoption for systematic landfill monitoring programs^78^.

Elevation-based height calculations require careful consideration of reference surface selection, as the choice of baseline elevation significantly affects the calculated waste height and subsequent volume estimates. The error analysis results presented in Table 1 demonstrate the methodology’s reliability, with validation through differential GPS surveys and total station measurements at Gondiya Landfill and Ujjain Ring Road Trenching Ground, providing essential calibration data for adjusting and validating remote sensing derived measurements across the broader dataset. The volume distribution observed in Figs. 6 and 7 clearly demonstrates the methodology’s capability to distinguish between different landfill characteristics, with sites like Chennai Dumping Ground and Deonar Municipal Dumping Ground showing distinctly higher volumes that correlate with their operational history and urban context.

A critical limitation of this study is that ground validation was conducted at only two landfill sites, both located near our research base. These sites were chosen due to logistical feasibility, accessibility, and resource constraints. While the validation provided robust cross-checking using DGPS, Total Station, and drone-based surveys, the limited number of sites does not fully capture the heterogeneity of landfills across India. Expanding validation to a larger and more geographically diverse set of sites would strengthen the robustness of the methodology and help reduce the observed variability in estimation errors. Also, methodological constraints could hinder the wider transferability and repeatability of this SAR interferometry technique for estimating landfill volume in varying ecological and operational contexts. Phase unwrapping remains a critical source of uncertainty, as errors in retrieving true phase values from wrapped phases can propagate through volume calculations, particularly in areas with steep topographic gradients or discontinuous waste surfaces that challenge conventional unwrapping algorithms^79,80^. Temporal decorrelation of SAR data frequently introduces significant elevation errors in DEMs derived from interferometric processing, leading to substantial volumetric change estimation errors in landfill applications^81,82^. The quality and resolution of reference DEMs fundamentally limit measurement precision, with coarse-resolution DEMs inadequately capturing micro-topographic variations essential for accurate volume change detection in complex landfill geometries^83,84^. Atmospheric effects, particularly tropospheric delays that vary spatially and temporally, introduce significant errors in repeat pass InSAR measurements, with effects potentially amplified in humid tropical climates or regions with high atmospheric variability^85,86^. These technical constraints collectively suggest that while the proposed methodology demonstrates viability for systematic landfill monitoring, careful site-specific calibration and validation would be essential for successful implementation across diverse geographic, climatic, and operational contexts.

The establishment of near real-time monitoring capabilities of landfill sites enables proactive management approaches, allowing authorities to identify rapidly filling sites, optimize collection routes, and implement timely interventions before capacity crises occur^15,87^. The standardization of landfill volume estimation procedures through remote sensing methodology facilitates comparative analysis between different sites, regions, and management practices, supporting evidence-based policy development and resource allocation decisions^88,89^. However, successful implementation requires addressing several challenges, including the need for technical expertise in remote sensing and GIS applications, access to appropriate software and computing resources, and the establishment of quality control procedures to ensure consistent results across different operators and time periods. The methodology’s dependence on satellite data availability and quality also necessitates contingency planning for data gaps or quality issues. Future developments should focus on automating key processing steps, developing user-friendly interfaces for non-technical users, and establishing standardized protocols for data collection, processing, and validation. The integration of additional remote sensing data sources, such as optical imagery and LiDAR measurements, could further enhance accuracy and provide complementary information for comprehensive landfill assessment and monitoring programs^12,90^, building upon the solid foundation established through this Sentinel-1 based approach.

## Conclusion

This study demonstrates the development and validation of a comprehensive remote sensing methodology for estimating landfill volumes across extensive geographical areas using Sentinel-1 SAR imagery. The proposed approach represents a significant advancement in waste management technology, offering a cost-effective, scalable, and standardized solution for monitoring landfill sites without the need for expensive ground-based surveys. Through the systematic analysis of 80 landfill sites distributed across India, this research establishes a robust framework that integrates Digital Elevation Model generation using Python-based approach integrated with SNAP, volume estimation through Google Earth Engine, and two landfill site survey for validation to ensure accuracy and reliability.

The methodology’s effectiveness is clearly demonstrated through the comprehensive results obtained from the diverse dataset spanning varying geographical and operational conditions. The height estimation analysis revealed distinct patterns based on landfill area, with sites below 60,000 m² showing waste heights appropriate for their scale, while larger sites exhibited significantly greater heights due to legacy waste accumulation. The validation study conducted at Gondiya Landfill and Ujjain Ring Road Trenching Ground using DGPS, drone surveys, and total station measurements provided essential ground truth data that estimates the accuracy of the remote sensing approach for the broader dataset.

The temporal monitoring capabilities inherent in Sentinel-1data provide opportunities for tracking volumetric changes over time, enabling authorities to identify trends, predict capacity requirements, and implement proactive management strategies. This capability is particularly valuable for developing countries facing rapid urbanization and limited waste management infrastructure, where early identification of problematic sites can prevent environmental crises and public health issues.

Future research directions should focus on enhancing automation through machine learning algorithms for boundary delineation, developing user-friendly interfaces for broader adoption by non-technical users, and integrating additional remote sensing data sources such as optical imagery and LiDAR measurements to further improve accuracy and provide complementary information. In conclusion, this study establishes a scalable methodology for remote landfill volume estimation that addresses critical needs in modern waste management while providing a foundation for future technological developments and practical implementation. The approach was validated at two representative sites, providing promising evidence of its accuracy, and was further demonstrated across 80 diverse landfill sites in India to highlight its robustness and applicability. While broader validation is still warranted, the results offer immediate practical benefits and establish a framework for continued advancement in remote sensing applications for environmental monitoring and management.

## Supplementary Information

Below is the link to the electronic supplementary material.

Supplementary Material 1

Supplementary Material 2

## Data Availability

The data sources used in this manuscript are fully referenced within the text. Any requests for additional information or data access can be directed to the corresponding author.
