# Supplementary material for: High-resolution landfill characterization using SAR remote sensing and cloud-based processing
Source: Sci Rep. 2025 Dec 21;16:3013. doi: 10.1038/s41598-025-32908-9 (PMC12830578; doi:10.1038/s41598-025-32908-9)
Supplement: Supplementary file 1 — Supplementary Material 1 [file 41598_2025_32908_MOESM1_ESM.pdf]

Appendix: A

High-resolution landfill characterization using SAR remote sensing and cloud-based processing

Shashank Agrawal[1], Shivukumar Rakkasagi[1*] and Manish Kumar Goyal[1]

[1] Department of Civil Engineering, Indian Institute of Technology Indore, India
[*]Corresponding author: Shivukumar Rakkasagi – Email ID: phd2101104006@iiti.ac.in
Address: Khandwa Road, Simrol, Indore Madhya Pradesh, 453552, India

---

1. SAR Interferometry Processing Methodology

The Digital Elevation Model (DEM) generation process employed in this study utilized a semi-automated approach leveraging the European Space Agency's (ESA) Sentinel Application Platform (SNAP) toolbox through its Python API, ESA SNAPPY[1]. The workflow encompassed the complete interferometric synthetic aperture radar (InSAR) processing chain, beginning with automated Sentinel-1 data acquisition and preprocessing, followed by interferogram generation, coherence estimation, and phase filtering operations executed through Python scripting. Subsequently, the processed interferometric products were exported in Statistical-Cost, Network-Flow Algorithm for Phase Unwrapping (SNAPHU)[2] compatible format for phase unwrapping, after which the unwrapped phase data was reimported into SNAP[3] for geocoding, phase-to-height conversion, and final DEM generation[4]. This integrated approach combines the computational efficiency of automated Python-based processing with the robust InSAR capabilities of the SNAP framework, enabling systematic and reproducible DEM extraction from Sentinel-1 data. All processing steps were implemented following the standardized workflow outlined in the Sentinel-1 Toolbox documentation and best practices for InSAR-based DEM generation[5]. This integrated

approach combines the computational efficiency of automated Python-based processing with the robust InSAR capabilities of the SNAP framework, enabling systematic and reproducible DEM extraction from Sentinel-1 data.

1.1 Data Acquisition and Preprocessing

The data acquisition process began by defining the Area of Interest (AOI) using a shapefile containing landfill boundary coordinates, which was loaded via Geo-Pandas and converted to EPSG:4326. Sentinel-1 Single Look Complex (SLC) data was acquired through the Alaska Satellite Facility (ASF)[6] DAAC API using authenticated requests, returning JSON metadata with orbit numbers, acquisition dates, and download URLs. The search results were organized by orbit tracks and sorted by absolute orbit numbers for optimal interferometric pair selection. The pair selection process prioritized temporal baseline optimization by selecting the earliest and latest acquisitions within the same relative orbit track, ensuring adequate temporal separation for deformation detection while maintaining interferometric coherence.

1.2 Steps of DEM Generation

The coregistration process begins with TOPSAR-Split operation, which extracts specific sub-swaths (IW1, IW2, or IW3) and polarizations (VV) from the original SLC products. Orbit file correction was applied to both master and slave images using automatically downloaded Sentinel Precise Orbit Ephemerides (POE) files. Back geocoding coregistration was performed using the Shuttle Radar Topography Mission (SRTM) 1 arcsecond Digital Elevation Model (DEM) as the reference surface. Interferogram generation involves complex multiplication of the coregistered master and conjugated slave images, followed by flat-earth phase removal and optional topographic phase subtraction. The TOPSAR-Deburst operation removes artificial phase

discontinuities and ensures phase continuity across burst boundaries within each sub-swath. Goldstein phase filtering was applied to reduce noise in the interferometric phase while preserving relevant deformation signals. The SNAPHU export process prepares the filtered interferogram for external phase unwrapping processing. The export operation converts SNAP-format products into SNAPHU compatible binary files along with comprehensive configuration parameters. The phase unwrapping procedure was executed using SNAPHU integrated within the SNAP environment. The unwrapped phase results were subsequently imported back into SNAP using the SNAPHU import function, which integrates the continuous phase information into the SNAP processing workflow. The unwrapped phase data was then converted to elevation information through the phase-to-elevation conversion module. Finally, Range-Doppler Terrain Correction was applied to geocode the elevation data, transforming it from the radar coordinate system to a geographic coordinate system while correcting for topographic distortions. This comprehensive processing chain ensures the generation of Digital Elevation Models suitable for quantitative analysis and geospatial applications[5,7–9].

2. On-site Landfill Parameters Estimation

This section includes drone imagery, contour maps, and elevation details obtained during the field visit of the Ujjain Ring Road Trenching Ground landfill and Gondiya landfill sites at Ujjain, Madhya Pradesh.

2.1 Ujjain Ring Road Trenching Ground Landfill Site

The drone survey at the landfill site captured high-resolution aerial images, which provide a detailed view of the site's waste accumulation, operational zones, and boundary extents shown in Fig. A.1 and Fig. A.2.

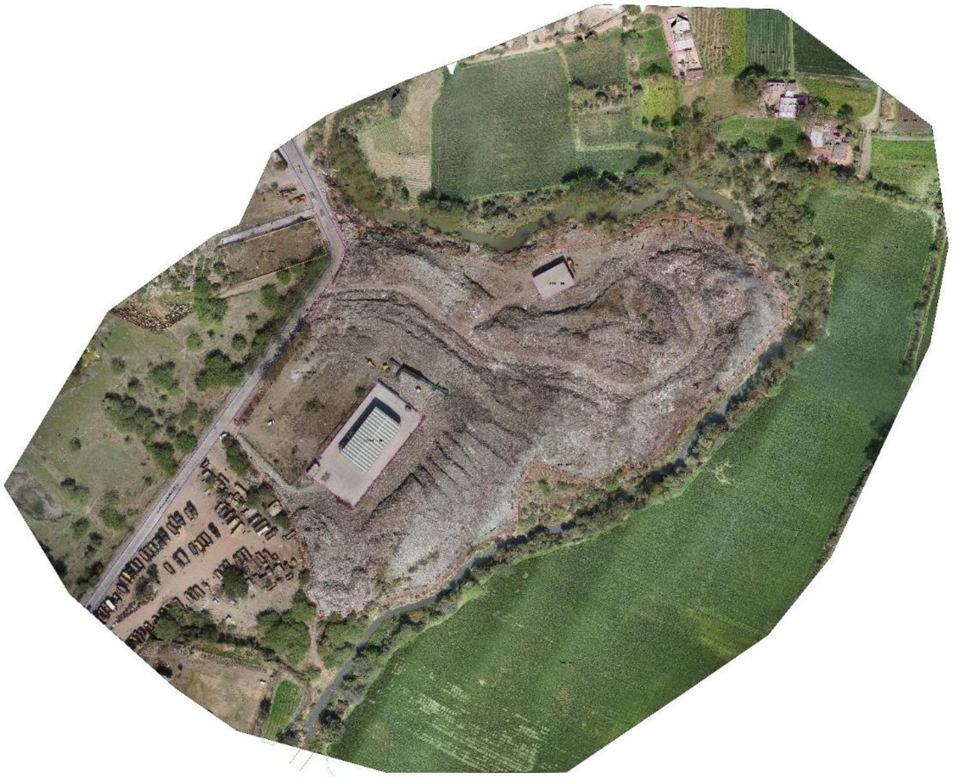

Fig. A.1. High resolution drone imagery of Ujjain Ring Road Trenching Ground.

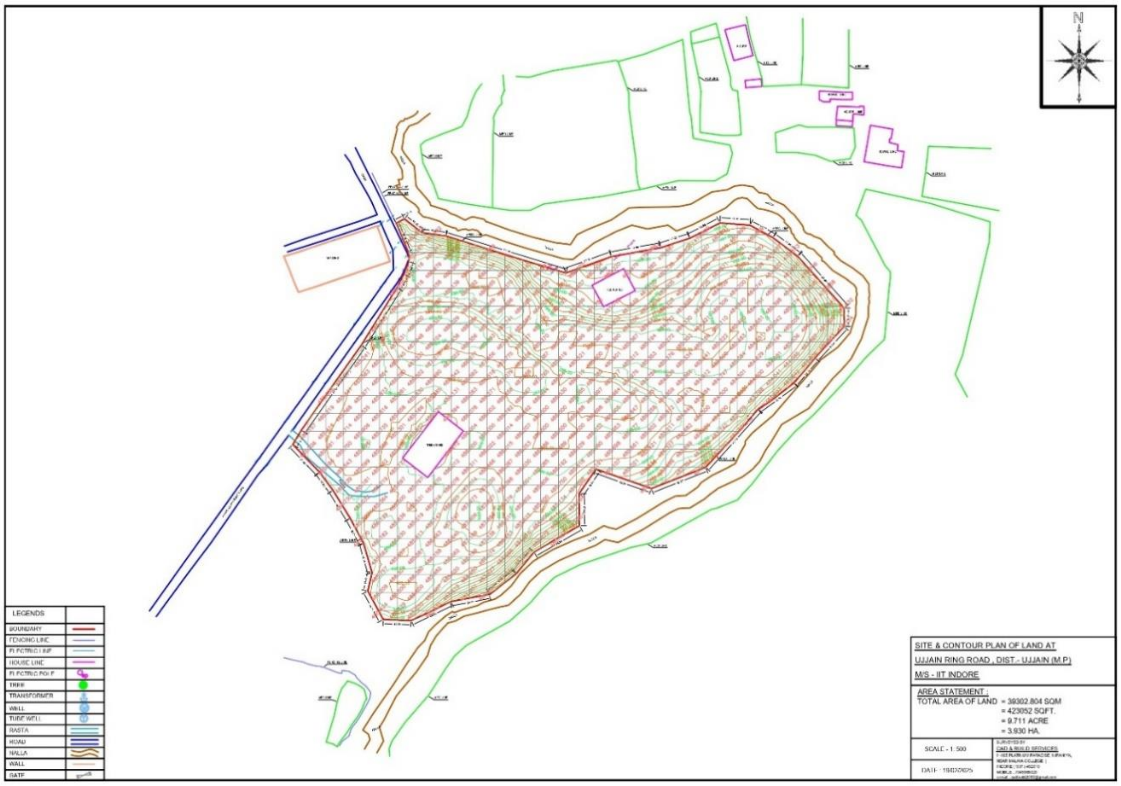

Fig. A.2 Site and Contour plan of land at Ujjain Ring Road trenching Ground landfill.

2.2 Gondiya Landfill Site

The drone images of the landfill site offer a broad aerial view of the waste spread and site contours as well as boundary extent as shown in Fig. A.3 and Fig. A.4.

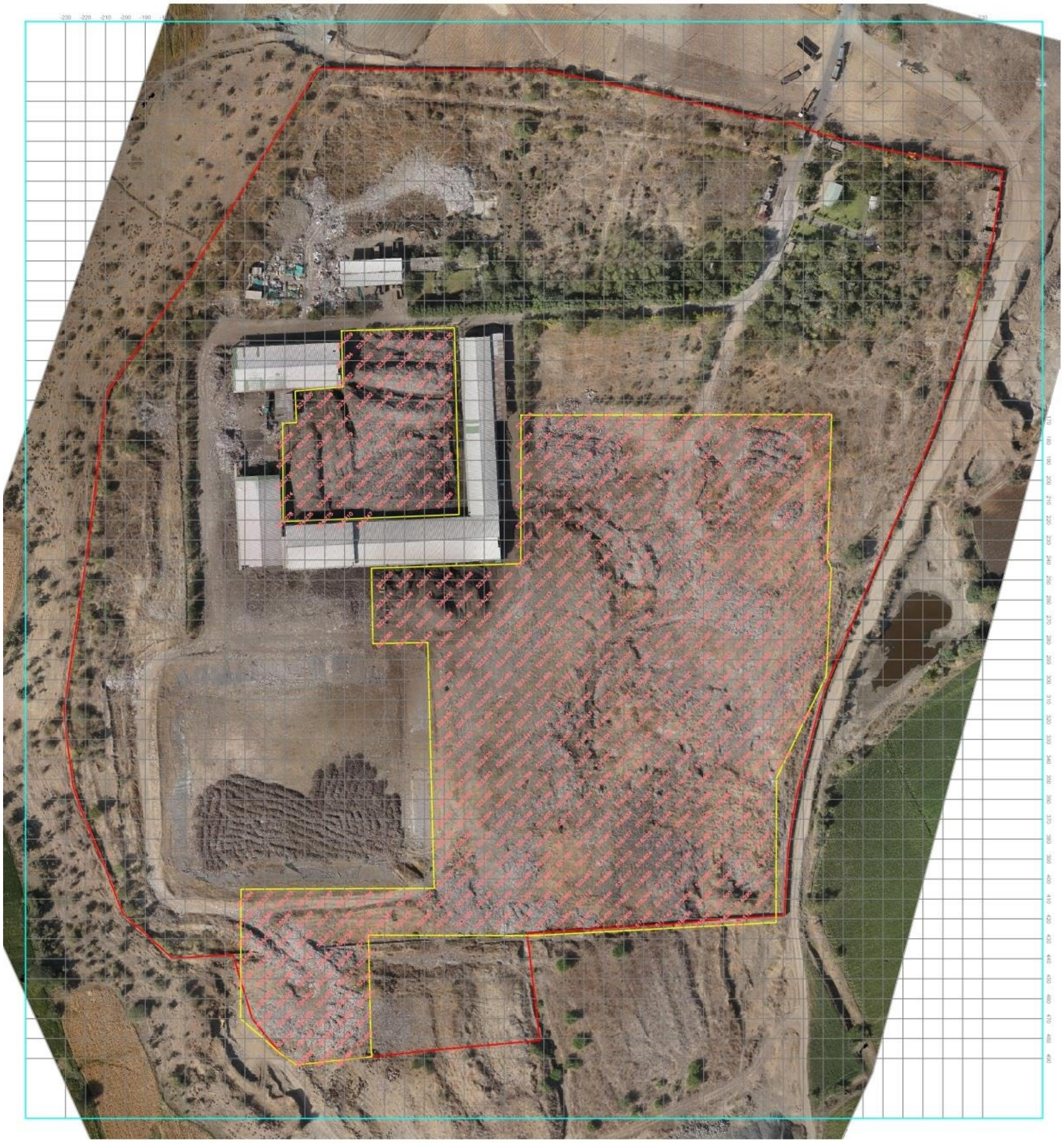

Fig. A.3 High resolution drone imagery Gondiya landfill.

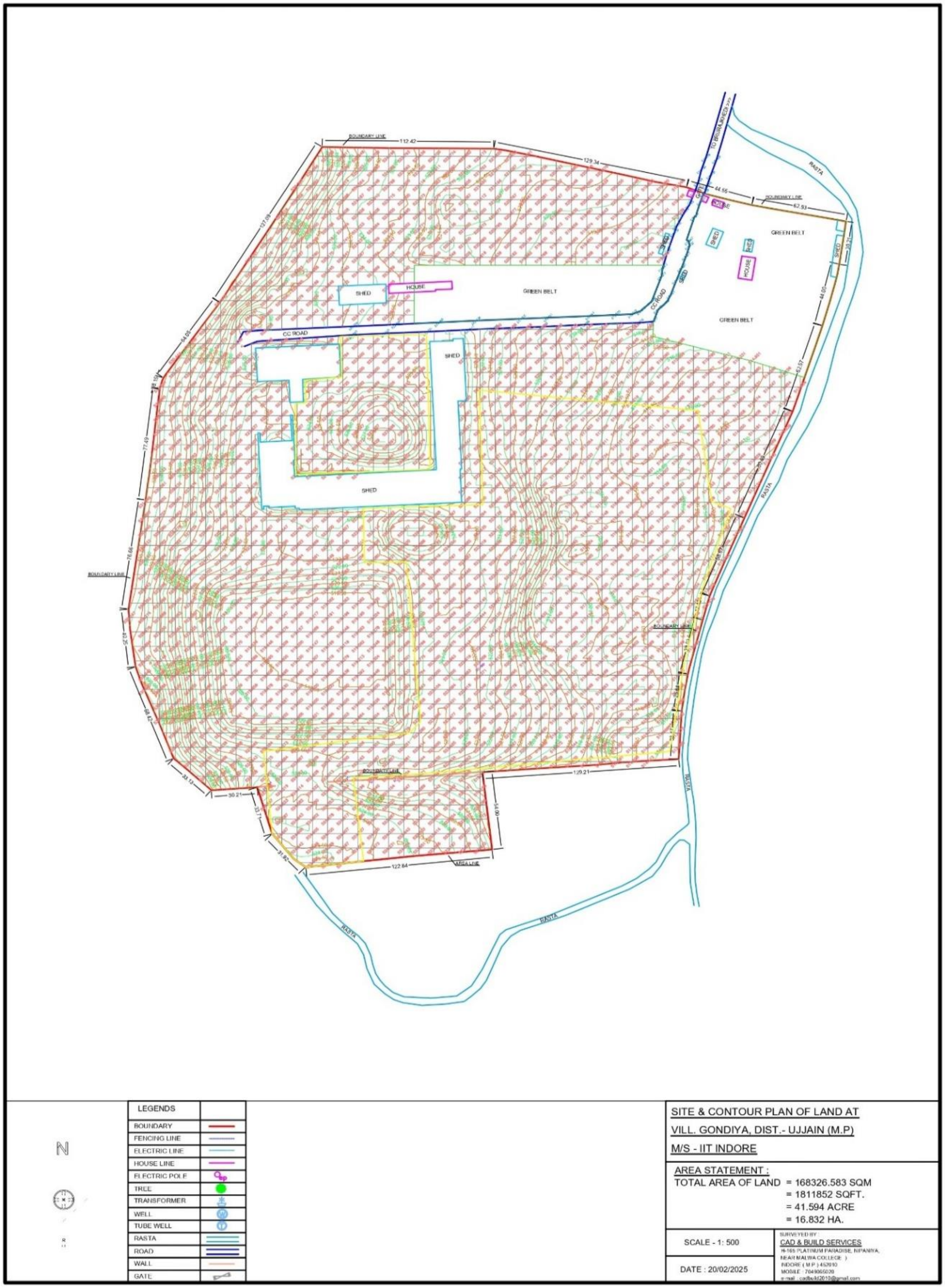

Fig. A.4 Site and Contour plan of land at Gondiya landfill.

2.3 Parameter details of the landfill site

The landfill site parameters obtained from the survey were shown in Table 1 and Table 2. The calculation of Average Top Level and Average original ground level was performed using the measured elevation values through Total Station.

Table A1. Details of estimated landfill parameters

**Landfill Site**	**Ujjain Ring Road Trenching Ground Landfill**	**Gondiya Landfill Site**
Average Top Level (H_{top})	484.84 m	520.84 m
Average Original Ground Level (H_{ground})	480.79 m	518.71 m
Average Height (H_{field})	4.047 m	2.13 m
Waste Spread Area (A_{field})	39,302.80 m^2	59,966.81 m^2
Waste Volume (V_{field})	1,59,096.30 m^3	1,27,783.48 m^3

References

1. Abad, L., Hölbling, D., Dabiri, Z. & Robson, B. An open-source Python package for DEM generation and landslide volume estimation based on Sentinel-1 imagery. at https://doi.org/10.5194/egusphere-egu22-693 (2022).
2. Chen, C. W., Zebker, H. A. SNAPHU: Statistical-cost, Network-flow Algorithm for Phase Unwrapping. at (2001).
3. European Space Agency. Sentinel Application Platform (SNAP). at https://step.esa.int/main/download/snap-download/previous-versions/ (2025).
4. Zaki, A. M. *et al.* Automated Python Workflow for Generating Sentinel-1 Psi and Sbas Interferometric Stacks Using Snap on Geospatial Computing Platform. at https://doi.org/10.2139/ssrn.4790006 (2024).
5. Braun, A. Retrieval of digital elevation models from Sentinel-1 radar data - Open applications, techniques, and limitations. *Open Geosci.* **13**, 532–569 (2021).
6. European Space Agency. Sentinel-1 SAR data. https://search.asf.alaska.edu/ (2025).
7. Zhang, S. *et al.* Verification of the accuracy of Sentinel-1 for DEM extraction error analysis under complex terrain conditions. *Int. J. Appl. Earth Obs. Geoinf.* **133**, 104157 (2024).
8. Soni, C., Chaudhary, A., Sharma, U. & Sharma, C. Satellite Radar Interferometry for DEM Generation Using Sentinel-1A Imagery. in 26–33 (2021). doi:10.1007/978-981-15-6067-5_4.

9. Nikolakopoulos, K. & Kyriou, A. Preliminary results of using Sentinel-1 SAR data for DSM generation. *Eur. J. Geogr.* **6**, 52–68 (2015).
