# Supplementary material for: High-resolution landfill characterization using SAR remote sensing and cloud-based processing
Source: Sci Rep. 2025 Dec 21;16:3013. doi: 10.1038/s41598-025-32908-9 (PMC12830578; doi:10.1038/s41598-025-32908-9)
Supplement: Supplementary file 2 — Supplementary Material 2 [file 41598_2025_32908_MOESM2_ESM.pdf]

Appendix B

High-resolution landfill characterization using SAR remote sensing and cloud-based processing

Shashank Agrawal[1], Shivukumar Rakkasagi[1*] and Manish Kumar Goyal[1]

[1] Department of Civil Engineering, Indian Institute of Technology Indore, India

[*]Corresponding author: Shivukumar Rakkasagi – Email ID: phd2101104006@iiti.ac.in

Address: Khandwa Road, Simrol, Indore Madhya Pradesh, 453552, India

---

This appendix shows the summary of the results for the remaining 78 landfill sites. The study sites were arranged in alphabetical order with necessary information of their place, location, average waste height (m), area of landfill site (m^2), volume of waste (m^3) and total pixel population within landfill site (N).

For each site three figures are shown, namely:

1.) Google Earth Imagery: Google Earth imagery of landfill sites highlighting the extent with yellow polygon. Google Earth imagery is copyrighted to Imagery © 2025 Google, Maxar Technologies, CNES / Airbus; Map data © 2025 Google. (URL: https://earth.google.com/web)

2.) Waste height (m) variation with change in pixel population (n): This figure shows the variation of average waste height (m) for varying pixel values (n) taken into consideration. All these figures were created using Microsoft Excel 365, Version 2510 (https://www.microsoft.com/en-in/microsoft-365/excel) .

3.) Landfill elevation variation: This figure depicts the elevation change within the landfill site obtained from the DEM generated from Sentinel-1 imagery. All these figures were created using QGIS 3.40.3 (Quantum Geographic Information System; https://download.qgis.org/downloads/) and the background shows the base-map of Google Satellite from HCMGIS Plugin.

Landfill Site 01: Aadharwadi Dumping Ground

Place: Mumbai, Maharashtra

Location: 19°15'1.20"N, 73° 7'6.97"E

Average Waste Height: 6.54 m

Area of Landfill: 79331.52 m^2

Estimated Volume of Waste: 518616.11 m^3

Total Pixel Population within AOI (N): 92

Sentinel-1 Image Dates: 07-Jan-2025 and 31-Jan-2025

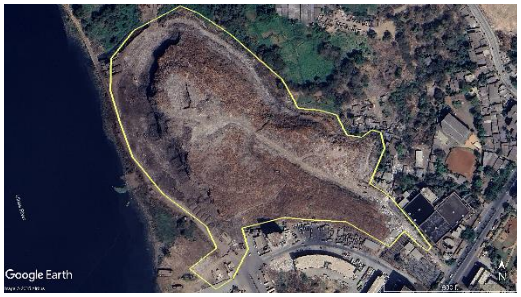

Fig. 1 Google Earth Imagery

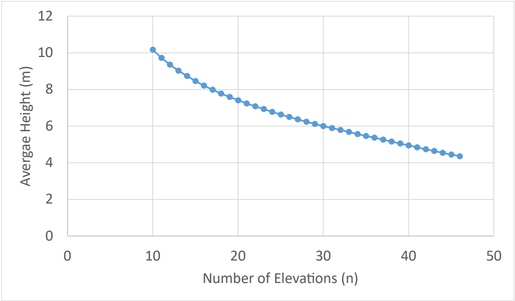

Fig. 2 Waste height (m) variation with change in pixel population (n)

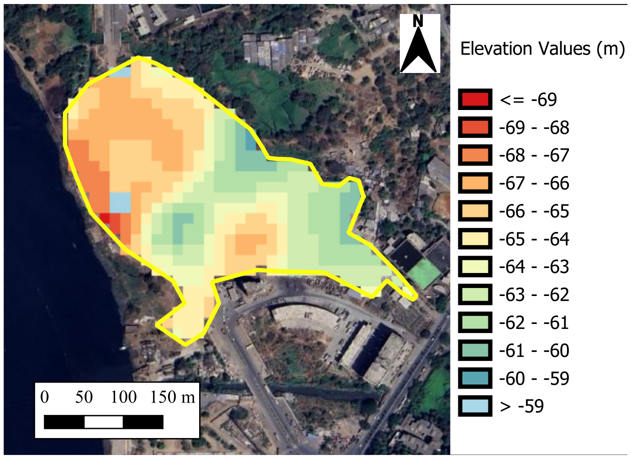

Fig. 3 Landfill elevation variations

Landfill Site 02: Ahemdabad Dumpsite

Place: Ahemdabad, Gujarat

Location: 22°58'50.62"N, 72°33'59.41"E

Average Waste Height: 8.84 m

Area of Landfill: 398796.53 m^2

Estimated Volume of Waste: 3526179.56 m^3

Total Pixel Population within AOI (N): 482

Sentinel-1 Image Dates: 07-Jan-2025 and 31-Jan-2025

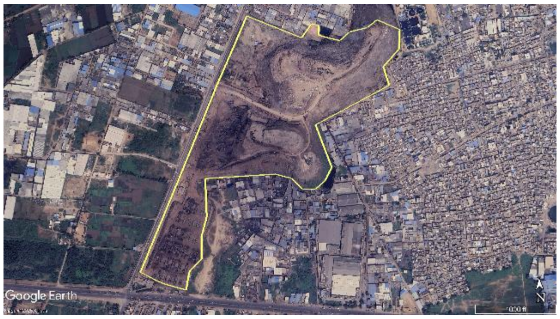

Fig. 4 Google Earth Imagery

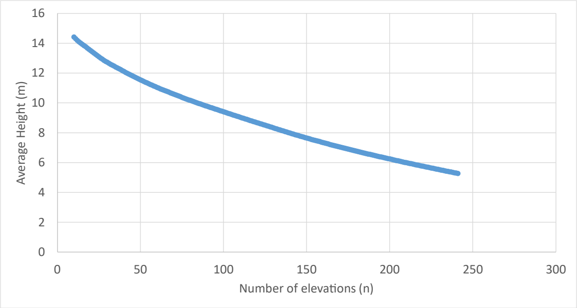

Fig. 5 Waste height (m) variation with change in pixel population (n)

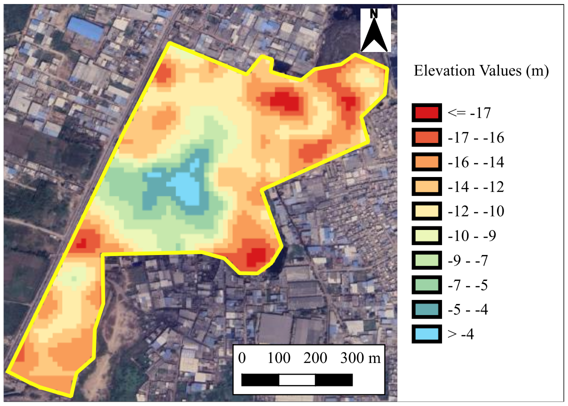

Fig. 6 Landfill elevation variations

Landfill Site 03: Ajmer Landfill

Place: Ajmer, Rajasthan

Location: 26°23'9.68"N, 74°40'44.01"E

Average Waste Height: 17.56 m

Area of Landfill: 174879.63 m^2

Estimated Volume of Waste: 3071126.45 m^3

Total Pixel Population within AOI (N): 215

Sentinel-1 Image Dates: 07-Jan-2025 and 31-Jan-2025

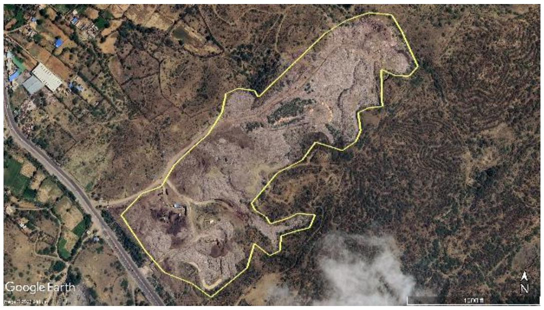

Fig. 7 Google Earth Imagery

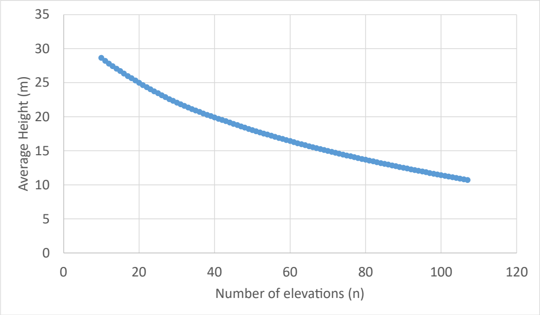

Fig. 8 Waste height (m) variation with change in pixel population (n)

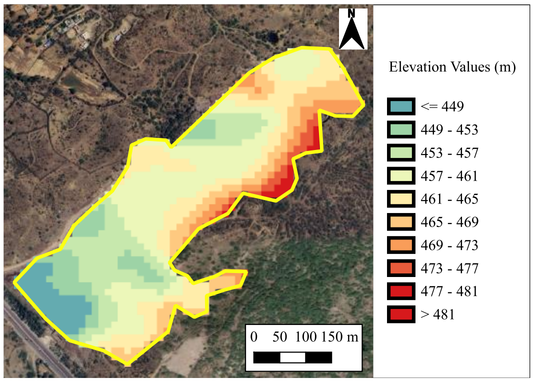

Fig. 9 Landfill elevation variations

Landfill Site 04: Alwar Landfill

Place: Alwar, Rajasthan

Location: 27°31'0.47"N, 76°45'15.19"E

Average Waste Height: 3.68 m

Area of Landfill: 98951.25 m^2

Estimated Volume of Waste: 364397.83m^3

Total Pixel Population within AOI (N): 123

Sentinel-1 Image Dates: 02-Jan-2025 and 26-Jan-2025

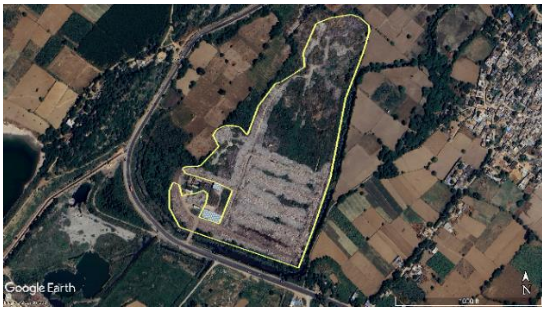

Fig. 10 Google Earth Imagery

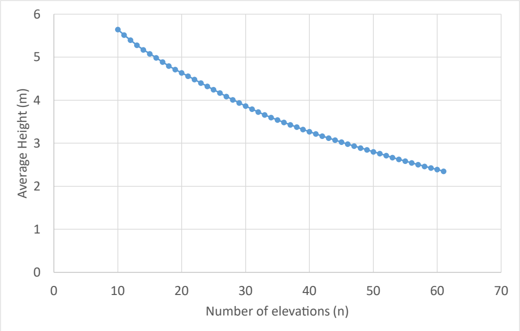

Fig. 11 Waste height (m) variation with change in pixel population (n)

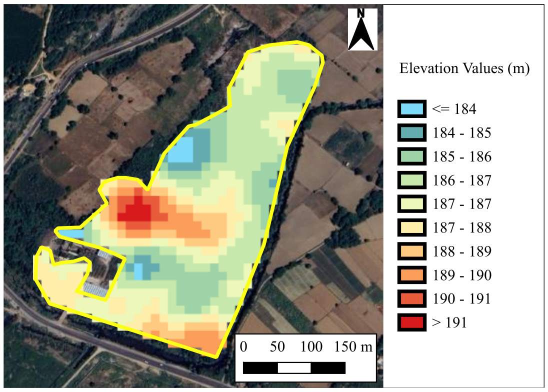

Fig. 12 Landfill elevation variations

Landfill Site 05: Amritsar Landfill

Place: Amritsar, Punjab

Location: 31°36'2.43"N, 74°52'27.82"E

Average Waste Height: 6.32 m

Area of Landfill: 114126.36 m^2

Estimated Volume of Waste: 720977.24 m^3

Total Pixel Population within AOI (N): 147

Sentinel-1 Image Dates: 07-Jan-2025 and 31-Jan-2025

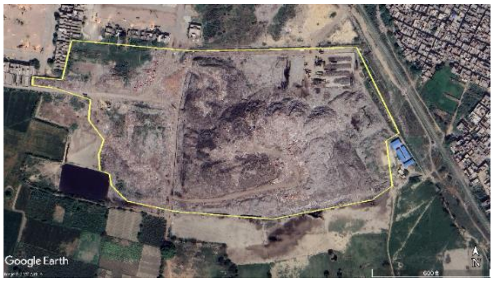

Fig. 13 Google Earth Imagery

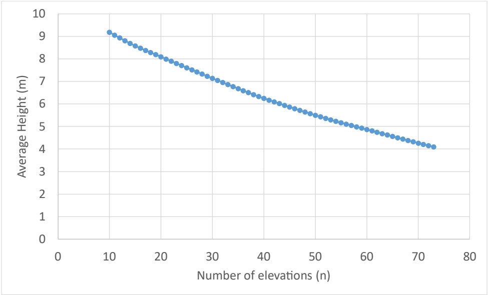

Fig. 14 Waste height (m) variation with change in pixel population (n)

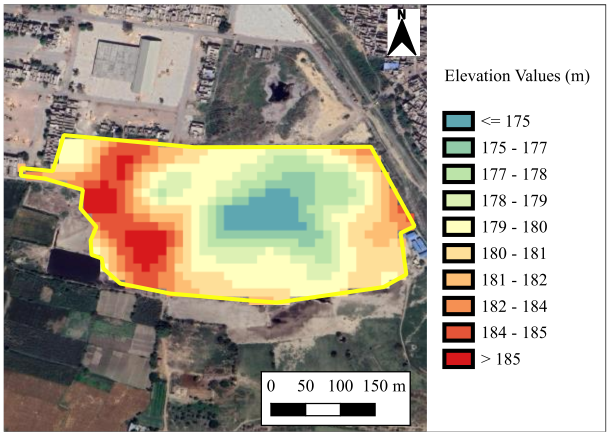

Fig. 15 Landfill elevation variations

Landfill Site 06: AP Gudivada Landfill

Place: Gudivada, Andhra Pradesh

Location: 16°27'49.79"N, 80°59'4.67"E

Average Waste Height: 1.15 m

Area of Landfill: 17388.10 m^2

Estimated Volume of Waste: 20042.53 m^3

Total Pixel Population within AOI (N): 22

Sentinel-1 Image Dates: 11-Jan-2025 and 23-Jan-2025

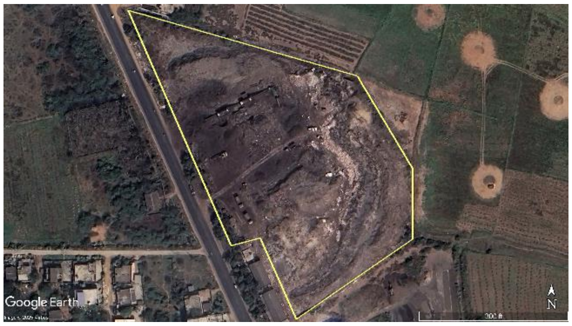

Fig. 16 Google Earth Imagery

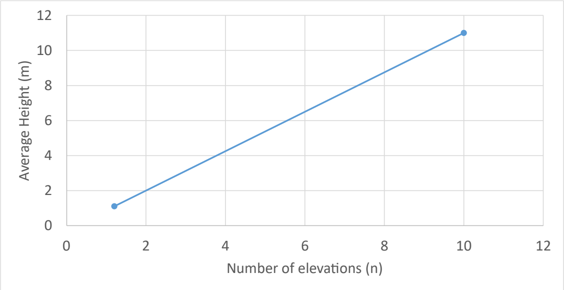

Fig. 17 Waste height (m) variation with change in pixel population (n)

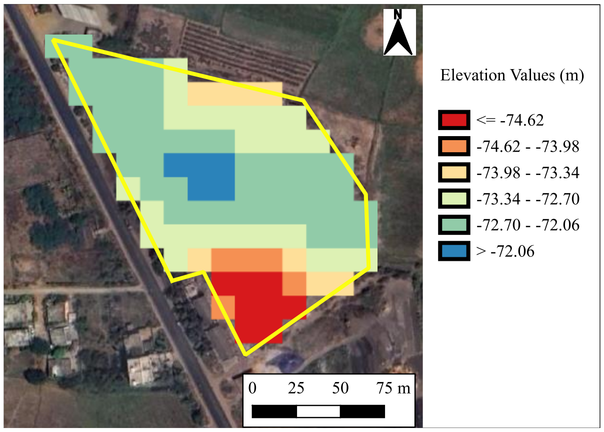

Fig. 18 Landfill elevation variations

Landfill Site 07: AP Kandukur Landfill

Place: Kandukur, Andhra Pradesh

Location: 15°12'15.95"N, 79°54'18.45"E

Average Waste Height: 1.88 m

Area of Landfill: 15208.15 m^2

Estimated Volume of Waste: 28596.64 m^3

Total Pixel Population within AOI (N): 20

Sentinel-1 Image Dates: 11-Jan-2025 and 23-Jan-2025

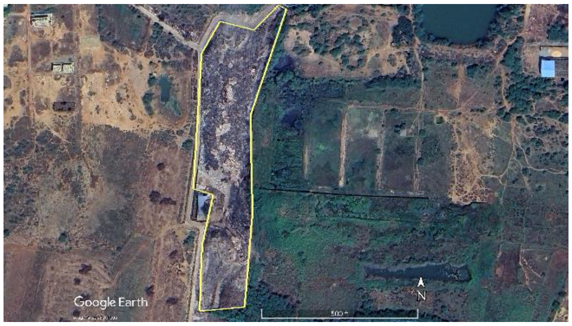

Fig. 19 Google Earth Imagery

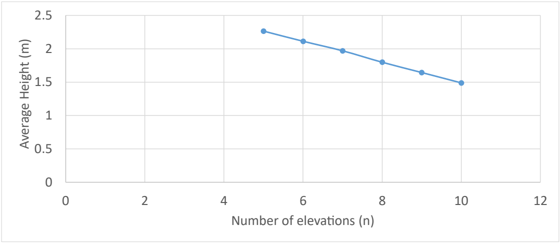

Fig. 20 Waste height (m) variation with change in pixel population (n)

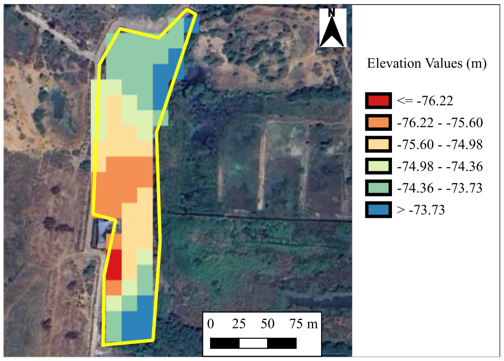

Fig. 21 Landfill elevation variations

Landfill Site 08: Arunachal Pradesh Itanagar Landfill

Place: Itanagar, Arunachal Pradesh

Location: 27° 2'43.53"N, 93°37'28.46"E

Average Waste Height: 0.47 m

Area of Landfill: 8465.13 m^2

Estimated Volume of Waste: 17894.77 m^3

Total Pixel Population within AOI (N): 23

Sentinel-1 Image Dates: 04-Jan-2025 and 28-Jan-2025

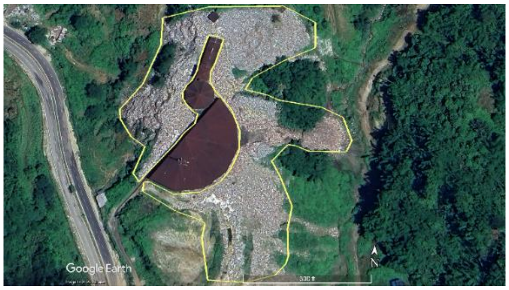

Fig. 22 Google Earth Imagery

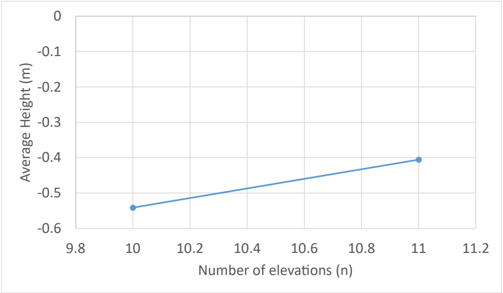

Fig. 23 Waste height (m) variation with change in pixel population (n)

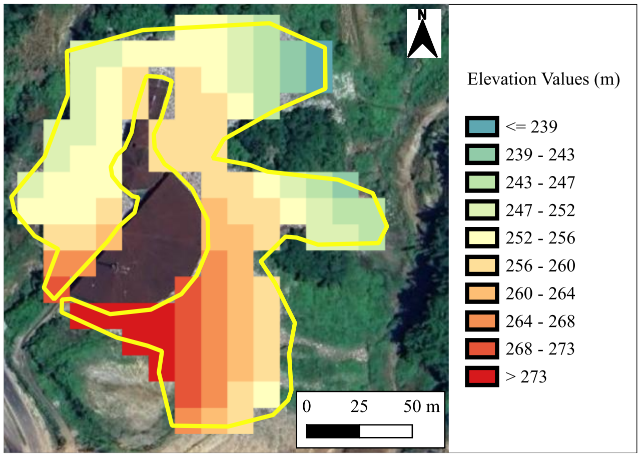

Fig. 24 Landfill elevation variations

Landfill Site 09: Assam Guwahati Landfill

Place: Guwahati, Assam

Location: 26° 6'47.95"N, 91°40'39.04"E

Average Waste Height: 3.63 m

Area of Landfill: 95752.04 m^2

Estimated Volume of Waste: 347431.33 m^3

Total Pixel Population within AOI (N): 119

Sentinel-1 Image Dates: 07-Jan-2025 and 31-Jan-2025

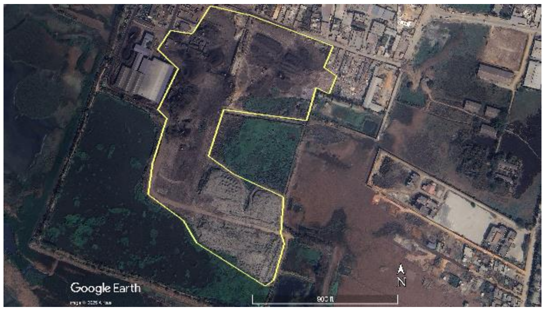

Fig. 25 Google Earth Imagery

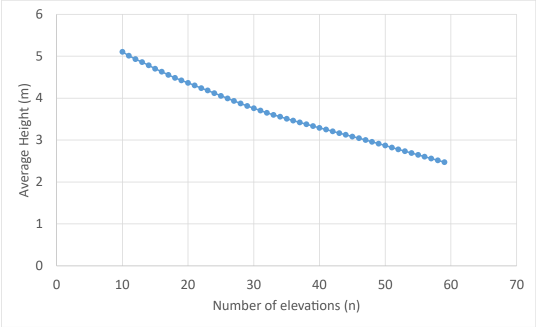

Fig. 26 Waste height (m) variation with change in pixel population (n)

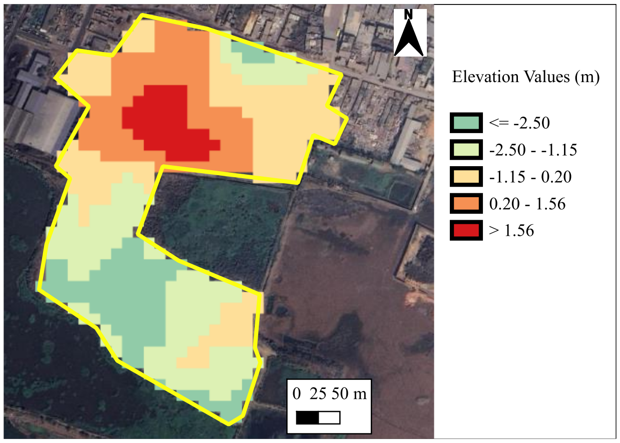

Fig. 27 Landfill elevation variations

Landfill Site 10: Assam Jorhat Landfill

Place: Jorhat, Assam

Location: 26°45'3.16"N, 94°13'27.31"E

Average Waste Height: 1.53 m

Area of Landfill: 21640.96 m^2

Estimated Volume of Waste: 33006.16 m^3

Total Pixel Population within AOI (N): 30

Sentinel-1 Image Dates: 02-Jan-2025 and 26-Jan-2025

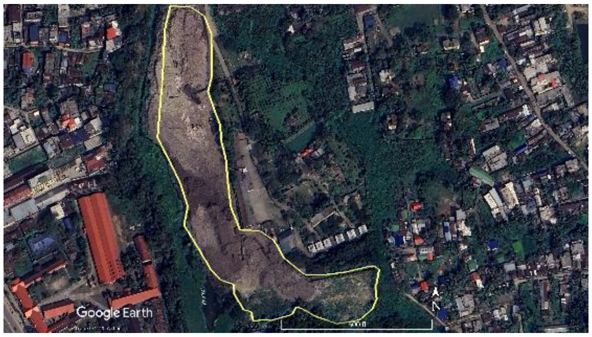

Fig. 28 Google Earth Imagery

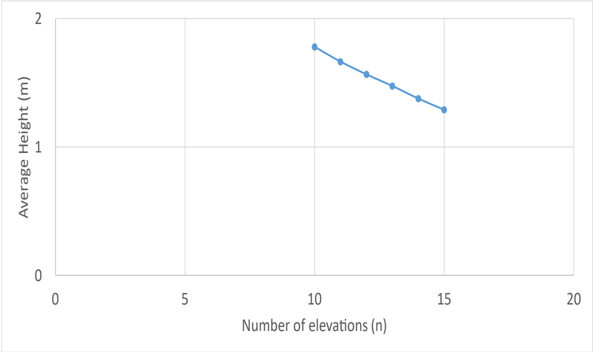

Fig. 29 Waste height (m) variation with change in pixel population (n)

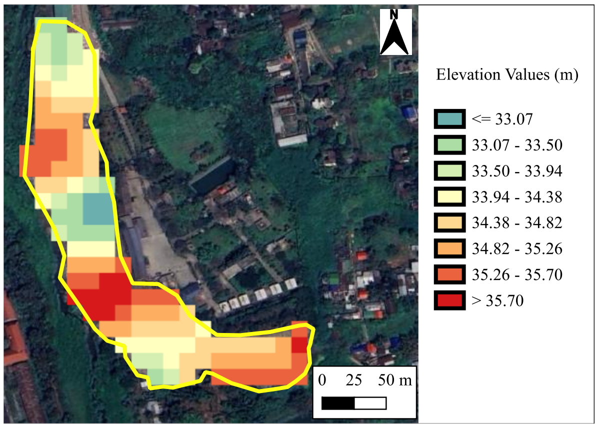

Fig. 30 Landfill elevation variations

Landfill Site 11: Aurangabad Landfill

Place: Aurangabad, Maharashtra

Location: 19°54'15.22"N, 75°17'9.86"E

Average Waste Height: 2.86 m

Area of Landfill: 46446.27 m^2

Estimated Volume of Waste: 132671.62 m^3

Total Pixel Population within AOI (N): 57

Sentinel-1 Image Dates: 14-Jan-2025 and 26-Jan-2025

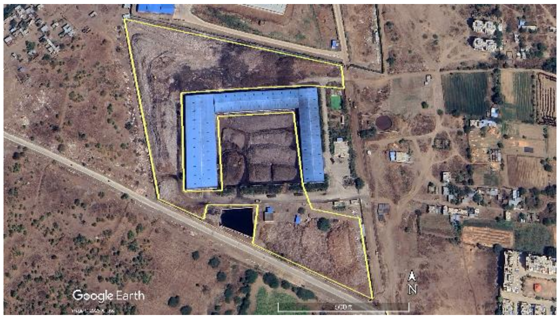

Fig. 31 Google Earth Imagery

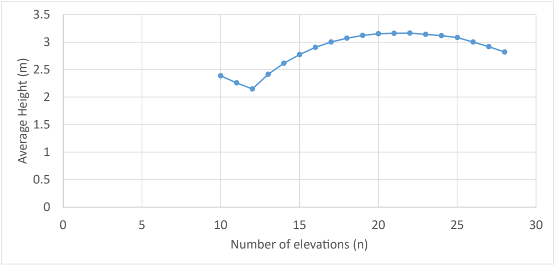

Fig. 32 Waste height (m) variation with change in pixel population (n)

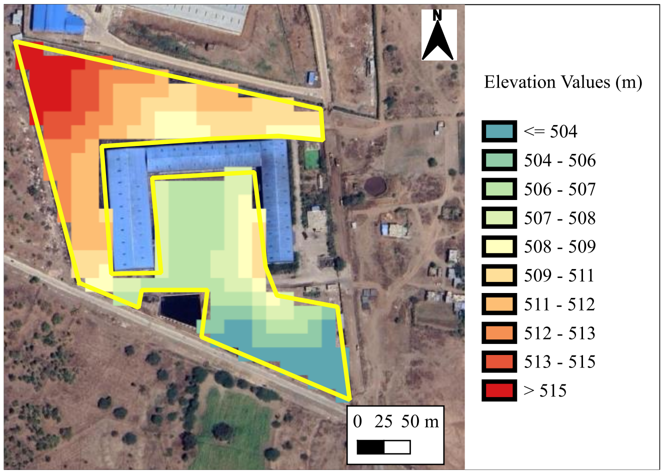

Fig. 33 Landfill elevation variations

Landfill Site 12: Balaghat Landfill

Place: Balaghat, Madhya Pradesh

Location: 21°47'51.34"N, 80° 9'17.44"E

Average Waste Height: 2.20 m

Area of Landfill: 45082.92 m^2

Estimated Volume of Waste: 98997.92 m^3

Total Pixel Population within AOI (N): 54

Sentinel-1 Image Dates: 04-Jan-2025 and 28-Jan-2025

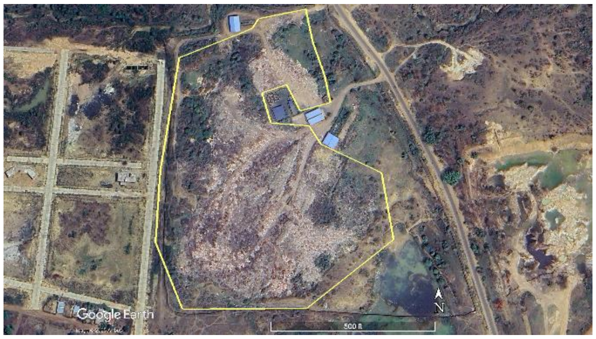

Fig. 34 Google Earth Imagery

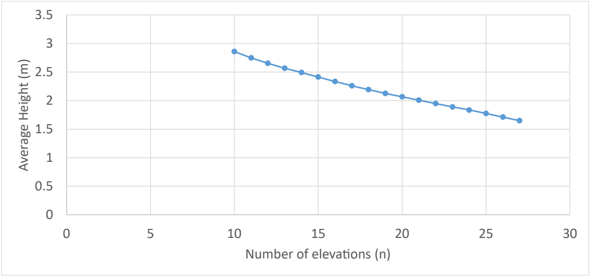

Fig. 35 Waste height (m) variation with change in pixel population (n)

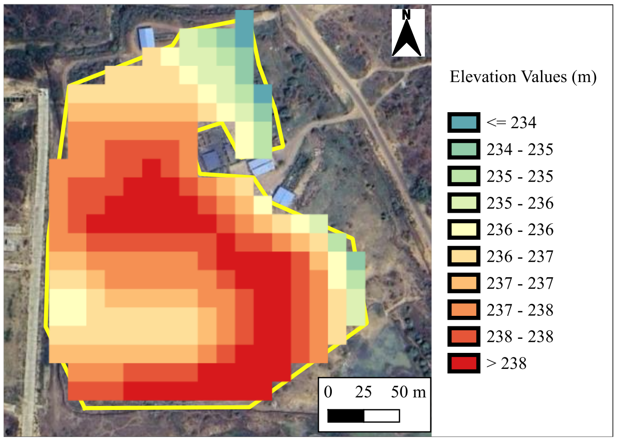

Fig. 36 Landfill elevation variations

Landfill Site 13: Bangaon Landfill

Place: Bangaon, West Bengal

Location: 23° 1'44.48"N, 88°50'32.50"E

Average Waste Height: 2.07 m

Area of Landfill: 17983.66 m^2

Estimated Volume of Waste: 37167.45 m^3

Total Pixel Population within AOI (N): 20

Sentinel-1 Image Dates: 02-Jan-2025 and 26-Jan-2025

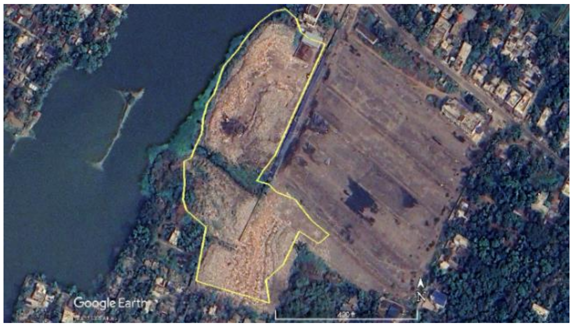

Fig. 37 Google Earth Imagery

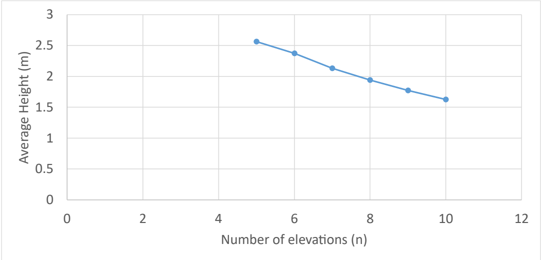

Fig. 38 Waste height (m) variation with change in pixel population (n)

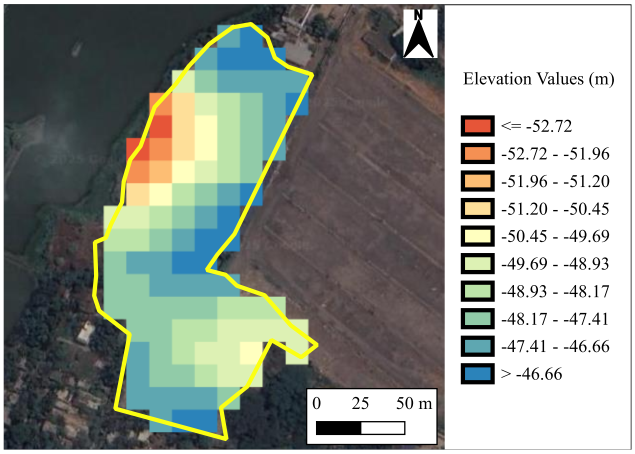

Fig. 39 Landfill elevation variations

Landfill Site 14: Bathinda Municipality Waste Disposal Landfill

Place: Bathinda, Punjab

Location: 30°10'55.55"N, 74°57'14.15"E

Average Waste Height: 7.92 m

Area of Landfill: 77292.80 m^2

Estimated Volume of Waste: 612344.77 m^3

Total Pixel Population within AOI (N): 102

Sentinel-1 Image Dates: 07-Jan-2025 and 31-Jan-2025

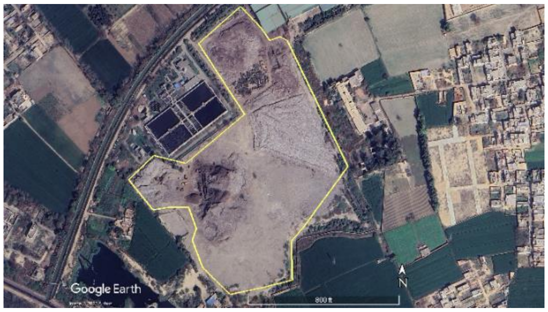

Fig. 40 Google Earth Imagery

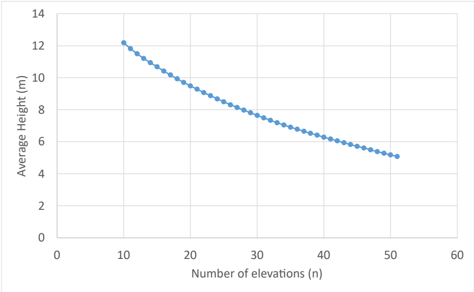

Fig. 41 Waste height (m) variation with change in pixel population (n)

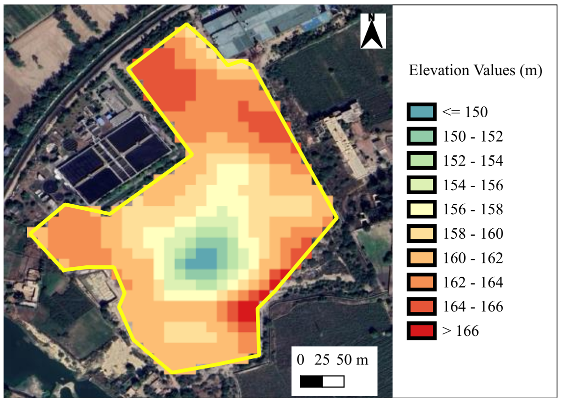

Fig. 42 Landfill elevation variations

Landfill Site 15: Bikaner Landfill

Place: Bikaner, Rajasthan

Location: 27°58'1.08"N, 73°20'48.69"E

Average Waste Height: 6.02 m

Area of Landfill: 119913.95 m^2

Estimated Volume of Waste: 721327.22 m^3

Total Pixel Population within AOI (N): 154

Sentinel-1 Image Dates: 07-Jan-2025 and 31-Jan-2025

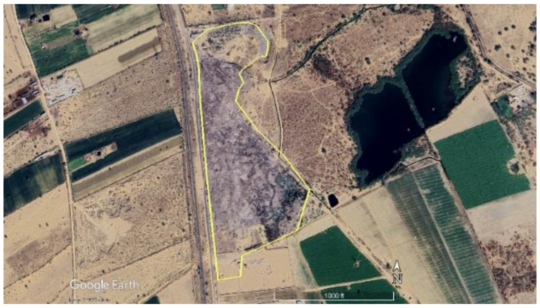

Fig. 43 Google Earth Imagery

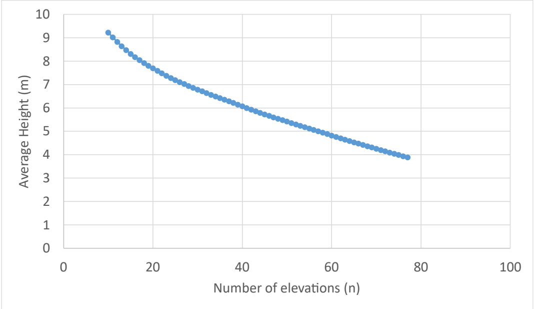

Fig. 44 Waste height (m) variation with change in pixel population (n)

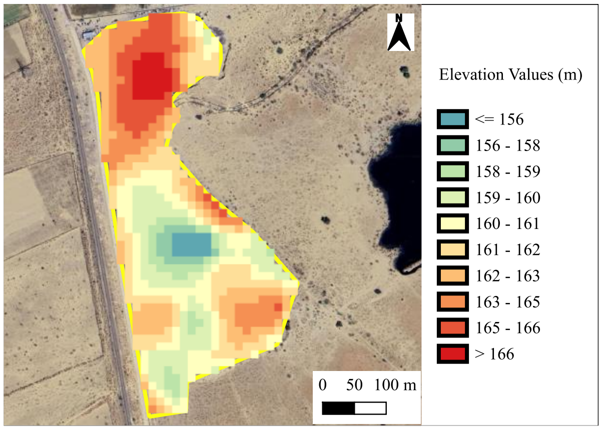

Fig. 45 Landfill elevation variations

Landfill Site 16: Chandigarh Landfill

Place: Chandigarh

Location: 30°45'26.61"N, 76°44'25.01"E

Average Waste Height: 7.49 m

Area of Landfill: 146431.95 m^2

Estimated Volume of Waste: 1096203.42 m^3

Total Pixel Population within AOI (N): 191

Sentinel-1 Image Dates: 06-Jan-2025 and 30-Jan-2025

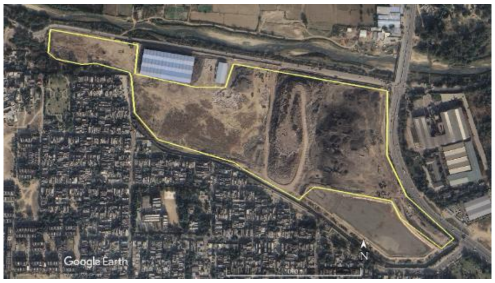

Fig. 46 Google Earth Imagery

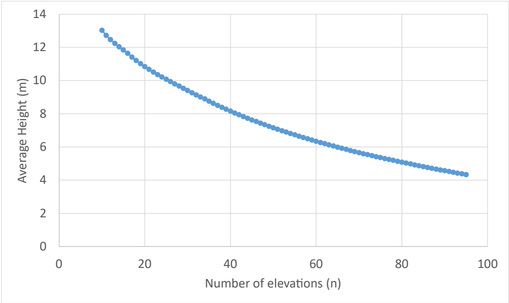

Fig. 47 Waste height (m) variation with change in pixel population (n)

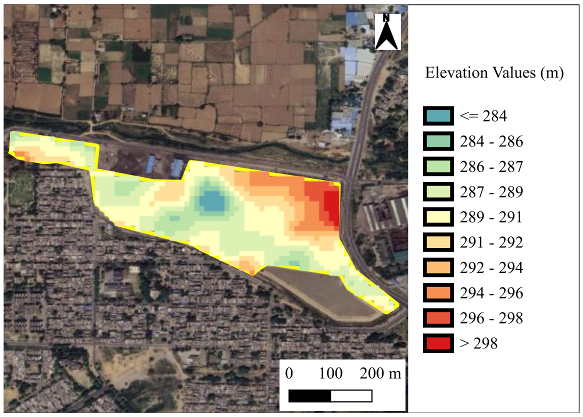

Fig. 48 Landfill elevation variations

Landfill Site 17: Chattisgarh Ambikapur Landfill

Place: Ambikapur, Chattisgarh

Location: 23° 5'35.31"N, 83° 8'57.00"E

Average Waste Height: 2.93 m

Area of Landfill: 34019.12 m^2

Estimated Volume of Waste: 99725.02 m^3

Total Pixel Population within AOI (N): 43

Sentinel-1 Image Dates: 06-Jan-2025 and 30-Jan-2025

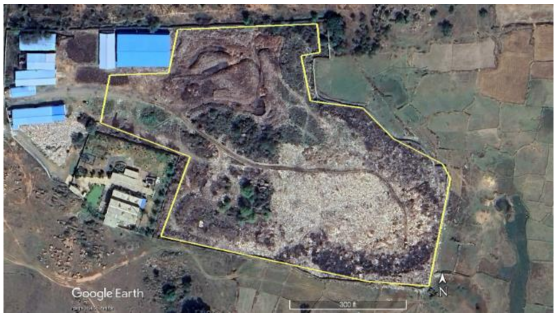

Fig. 49 Google Earth Imagery

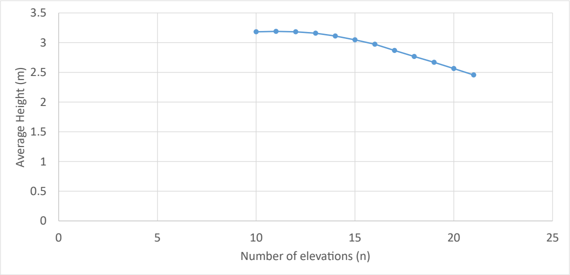

Fig. 50 Waste height (m) variation with change in pixel population (n)

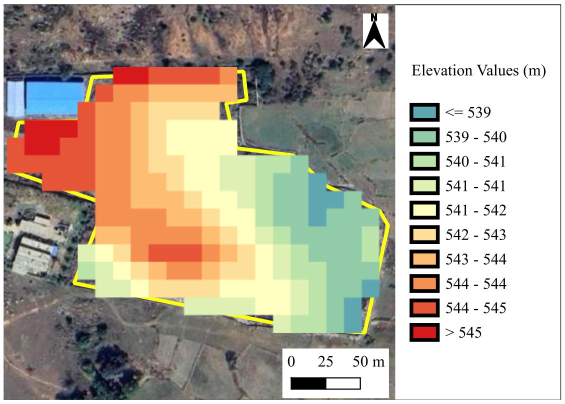

Fig. 51 Landfill elevation variations

Landfill Site 18: Chennai Dumping Ground

Place: Chennai, Tamil Nadu

Location: 13° 8'7.67"N, 80°16'6.65"E

Average Waste Height: 39.05 m

Area of Landfill: 862570.76 m^2

Estimated Volume of Waste: 33684541.94 m^3

Total Pixel Population within AOI (N): 985

Sentinel-1 Image Dates: 11-Jan-2025 and 23-Jan-2025

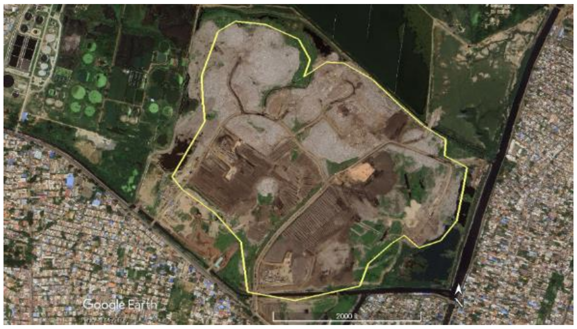

Fig. 52 Google Earth Imagery

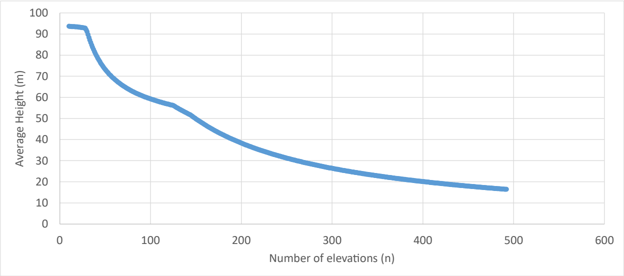

Fig. 53 Waste height (m) variation with change in pixel population (n)

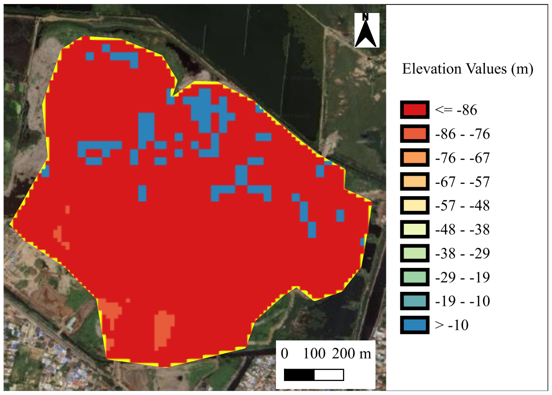

Fig. 54 Landfill elevation variations

Landfill Site 19: Chhatisgarh Rajnandgaon Landfill

Place: Rajnandgaon, Chattisgarh

Location: 21° 7'10.23"N, 81° 0'26.57"E

Average Waste Height: 1.72 m

Area of Landfill: 18407.81 m^2

Estimated Volume of Waste: 31729.28 m^3

Total Pixel Population within AOI (N): 20

Sentinel-1 Image Dates: 11-Jan-2025 and 23-Jan-2025

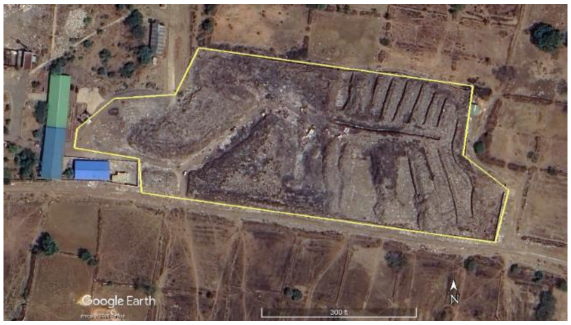

Fig. 55 Google Earth Imagery

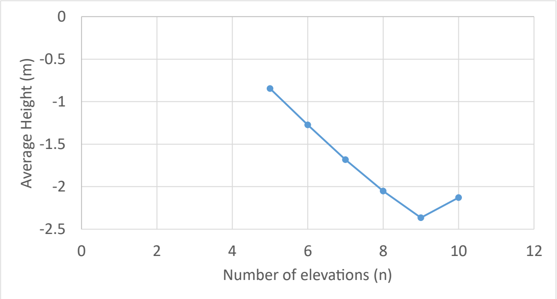

Fig. 56 Waste height (m) variation with change in pixel population (n)

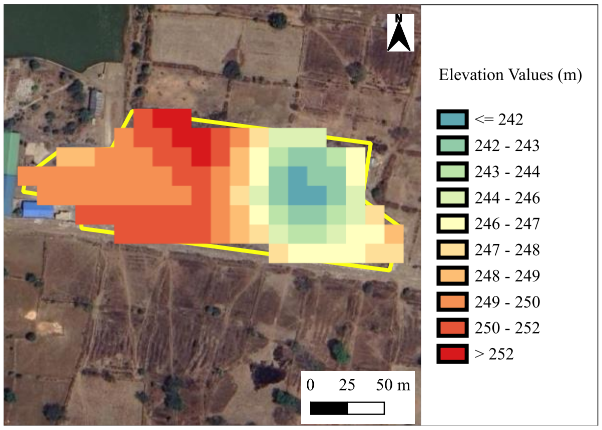

Fig. 57 Landfill elevation variations

Landfill Site 20: Delhi MCD Dumping Ground

Place: New Delhi

Location: 28°44'29.25"N, 77° 9'25.32"E

Average Waste Height: 9.95 m

Area of Landfill: 255495.21 m^2

Estimated Volume of Waste: 2541277.11 m^3

Total Pixel Population within AOI (N): 324

Sentinel-1 Image Dates: 02-Jan-2025 and 26-Jan-2025

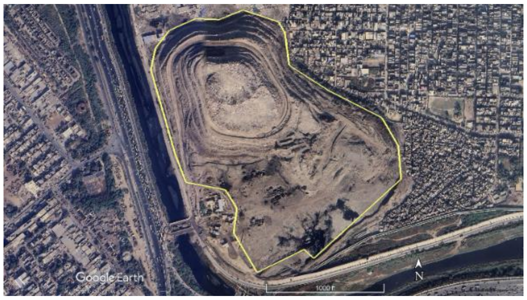

Fig. 58 Google Earth Imagery

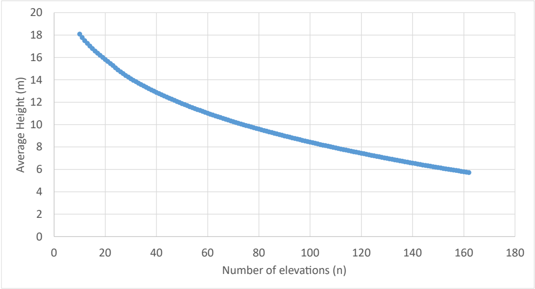

Fig. 59 Waste height (m) variation with change in pixel population (n)

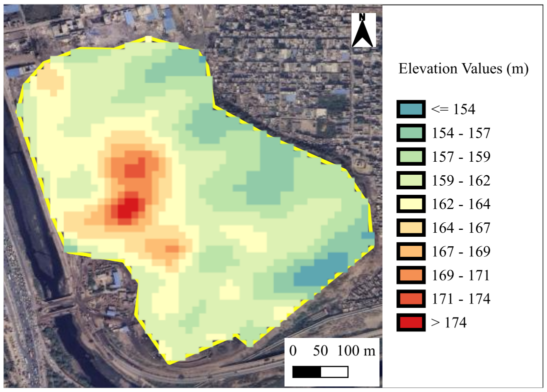

Fig. 60 Landfill elevation variations

Landfill Site 21: Deonar Municipal Dumping Ground

Place: Mumbai, Maharashtra

Location: 19° 4'10.55"N, 72°55'49.94"E

Average Waste Height: 24.25 m

Area of Landfill: 1062574.54 m^2

Estimated Volume of Waste: 25765470.45 m^3

Total Pixel Population within AOI (N): 1254

Sentinel-1 Image Dates: 07-Jan-2025 and 31-Jan-2025

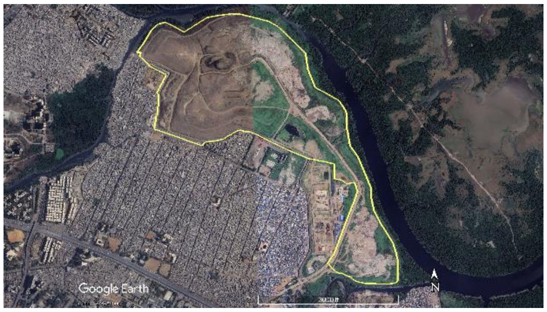

Fig. 61 Google Earth Imagery

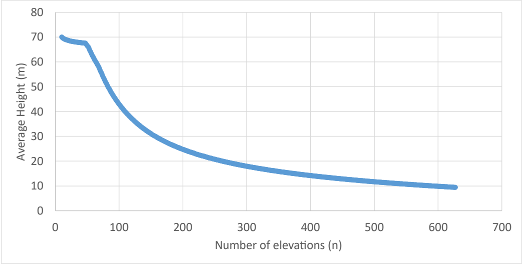

Fig. 62 Waste height (m) variation with change in pixel population (n)

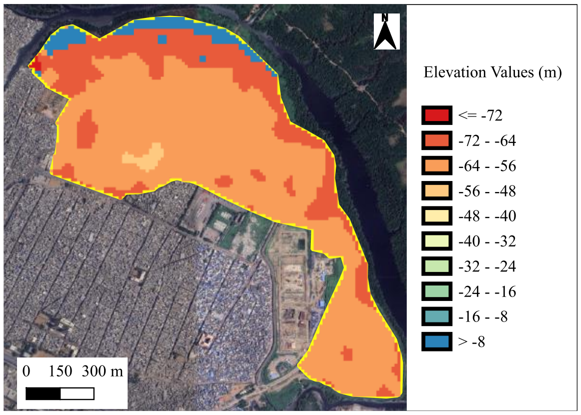

Fig. 63 Landfill elevation variations

Landfill Site 22: Durgapur Waste Dumping

Place: Durgapur, West Bengal

Location: 23°32'34.98"N, 87°21'34.66"E

Average Waste Height: 1.80 m

Area of Landfill: 44112.43 m^2

Estimated Volume of Waste: 79541.91 m^3

Total Pixel Population within AOI (N): 55

Sentinel-1 Image Dates: 08-Jan-2025 and 20-Jan-2025

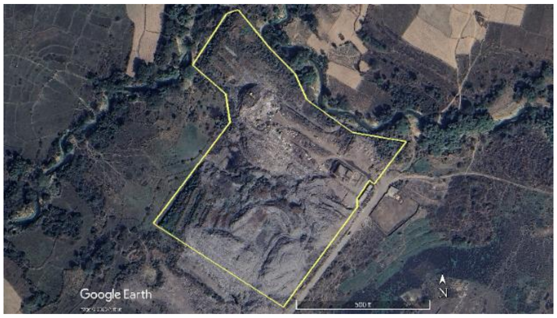

Fig. 64 Google Earth Imagery

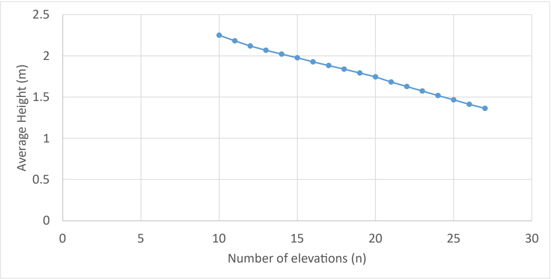

Fig. 65 Waste height (m) variation with change in pixel population (n)

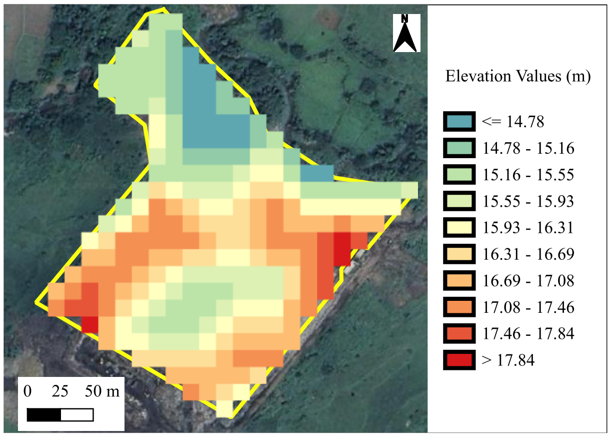

Fig. 66 Landfill elevation variations

Landfill Site 23: Ghazipur Landfill

Place: Ghazipur Area, New Delhi

Location: 28°37'28.26"N, 77°19'40.30"E

Average Waste Height: 8.83 m

Area of Landfill: 274827.27 m^2

Estimated Volume of Waste: 2426434.05 m^3

Total Pixel Population within AOI (N): 348

Sentinel-1 Image Dates: 02-Jan-2025 and 26-Jan-2025

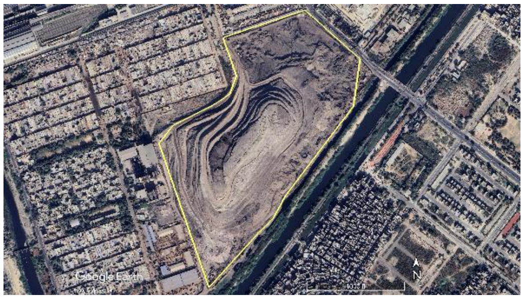

Fig. 67 Google Earth Imagery

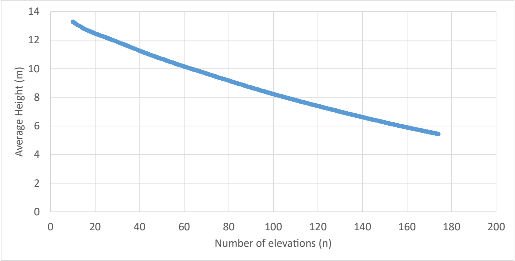

Fig. 68 Waste height (m) variation with change in pixel population (n)

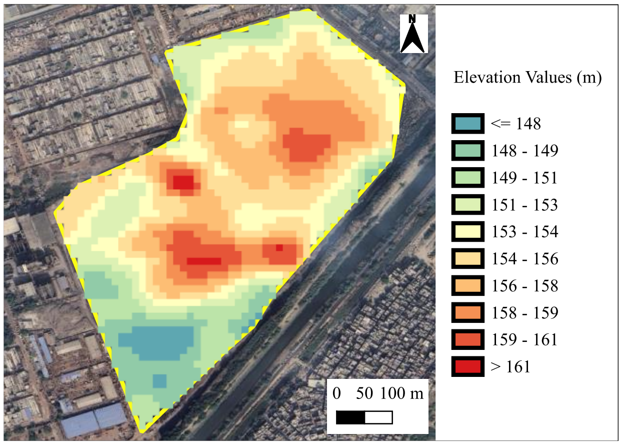

Fig. 69 Landfill elevation variations

Landfill Site 24: Harda Landfill

Place: Harda, Madhya Pradesh

Location: 22°20'15.75"N, 77° 4'41.34"E

Average Waste Height: 1.66 m

Area of Landfill: 28263.86 m^2

Estimated Volume of Waste: 47018.07 m^3

Total Pixel Population within AOI (N): 36

Sentinel-1 Image Dates: 09-Jan-2025 and 21-Jan-2025

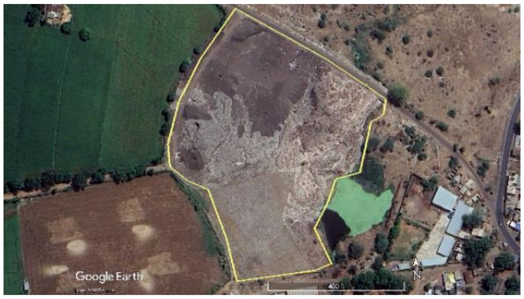

Fig. 70 Google Earth Imagery

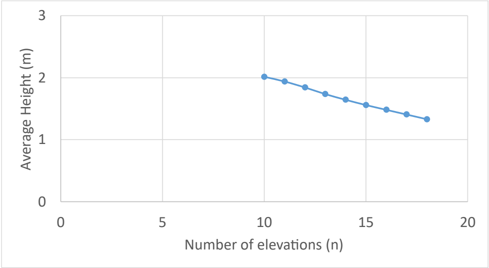

Fig. 71 Waste height (m) variation with change in pixel population (n)

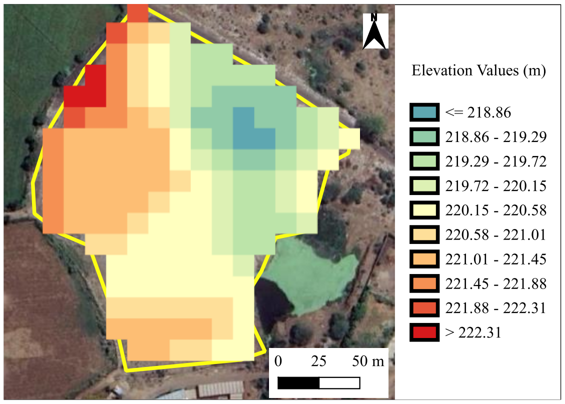

Fig. 72 Landfill elevation variations

Landfill Site 25: Haryana Faridabad Landfill

Place: Faridabad, Haryana

Location: 28°27'14.53"N, 77°19'12.44"E

Average Waste Height: 2.83 m

Area of Landfill: 13233.63 m^2

Estimated Volume of Waste: 37456.02 m^3

Total Pixel Population within AOI (N): 17

Sentinel-1 Image Dates: 02-Jan-2025 and 26-Jan-2025

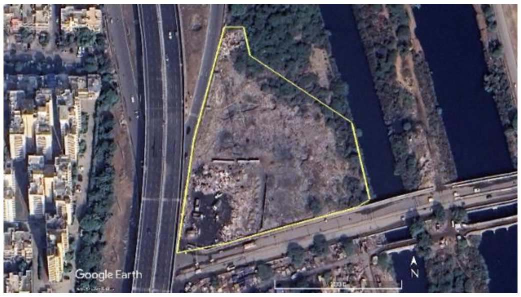

Fig. 73 Google Earth Imagery

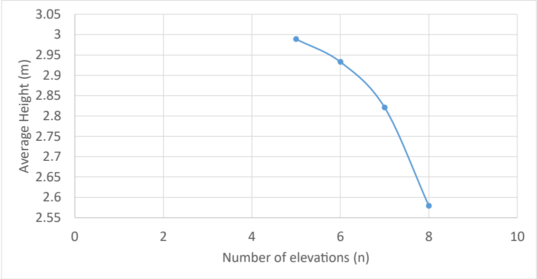

Fig. 74 Waste height (m) variation with change in pixel population (n)

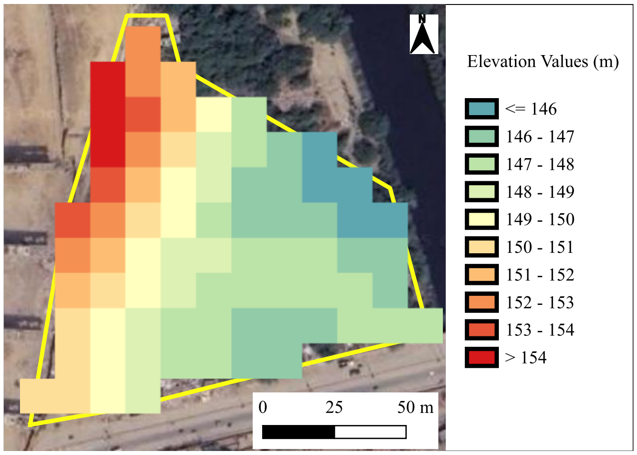

Fig. 75 Landfill elevation variations

Landfill Site 26: Haryana Gurugram Landfill

Place: Gurugram, Haryana

Location: 28°24'7.44"N, 77°10'17.70"E

Average Waste Height: 12.31 m

Area of Landfill: 119410.68 m^2

Estimated Volume of Waste: 1470093.45 m^3

Total Pixel Population within AOI (N): 153

Sentinel-1 Image Dates: 02-Jan-2025 and 26-Jan-2025

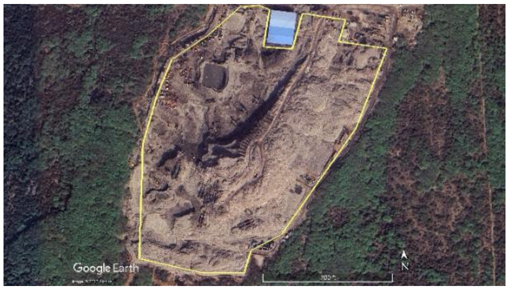

Fig. 76 Google Earth Imagery

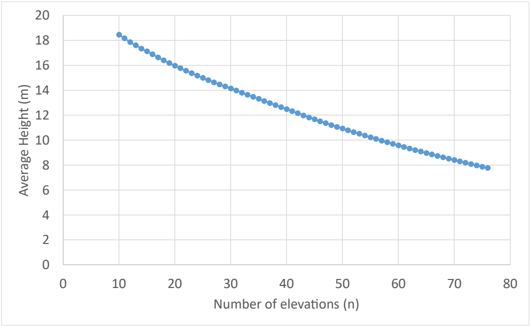

Fig. 77 Waste height (m) variation with change in pixel population (n)

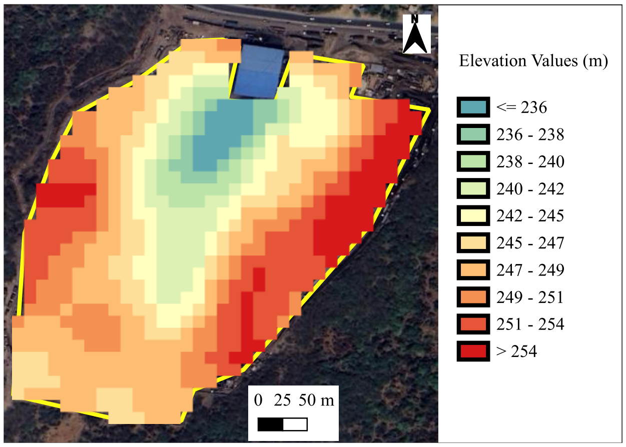

Fig. 78 Landfill elevation variations

Landfill Site 27: Punjab Kharar Landfill

Place: Kharar, Punjab

Location: 30°45'4.81"N, 76°37'23.15"E

Average Waste Height: 2.56 m

Area of Landfill: 35992.17 m^2

Estimated Volume of Waste: 92188.35 m^3

Total Pixel Population within AOI (N): 47

Sentinel-1 Image Dates: 02-Jan-2025 and 26-Jan-2025

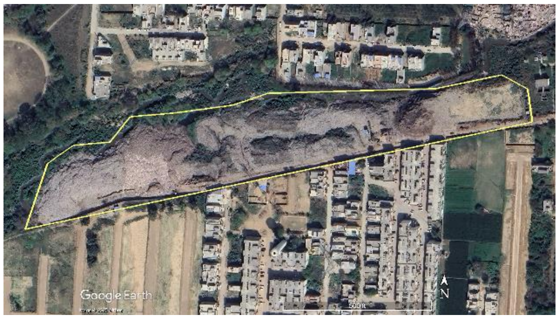

Fig. 79 Google Earth Imagery

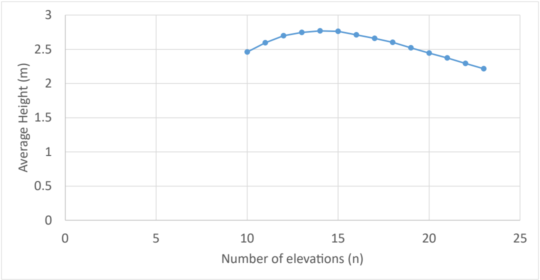

Fig. 80 Waste height (m) variation with change in pixel population (n)

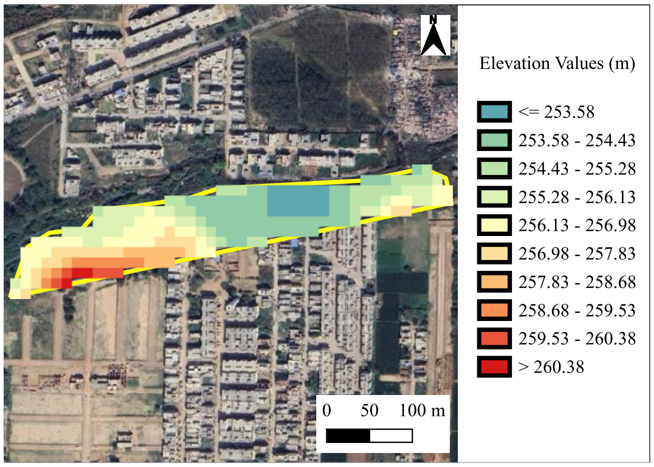

Fig. 81 Landfill elevation variations

Landfill Site 28: Haryana Panchkula Landfill

Place: Panchkula, Haryana

Location: 30°40'44.44"N, 76°53'31.99"E

Average Waste Height: 3.66 m

Area of Landfill: 33334.20 m^2

Estimated Volume of Waste: 121929.12 m^3

Total Pixel Population within AOI (N): 41

Sentinel-1 Image Dates: 02-Jan-2025 and 26-Jan-2025

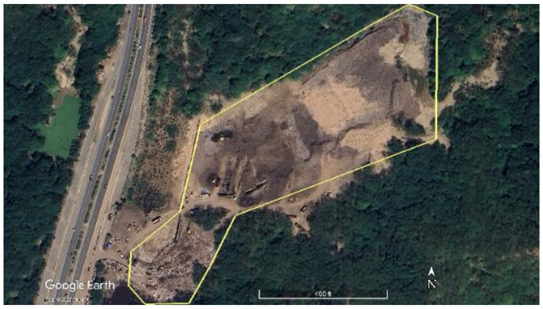

Fig. 82 Google Earth Imagery

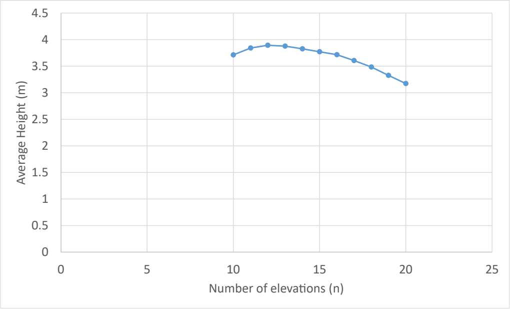

Fig. 83 Waste height (m) variation with change in pixel population (n)

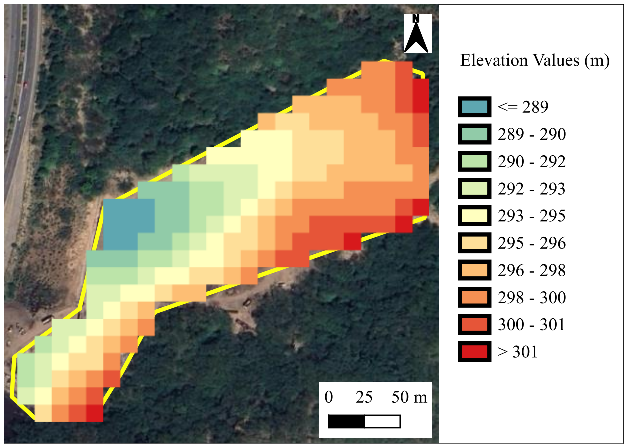

Fig. 84 Landfill elevation variations

Landfill Site 29: Hubbali Dharwad Municipal Corporation Landfill

Place: Hubbali, Karnataka

Location: 15°19'27.39"N, 75° 6'37.22"E

Average Waste Height: 9.12 m

Area of Landfill: 90301.73 m^2

Estimated Volume of Waste: 823591.17 m^3

Total Pixel Population within AOI (N): 102

Sentinel-1 Image Dates: 09-Jan-2025 and 21-Jan-2025

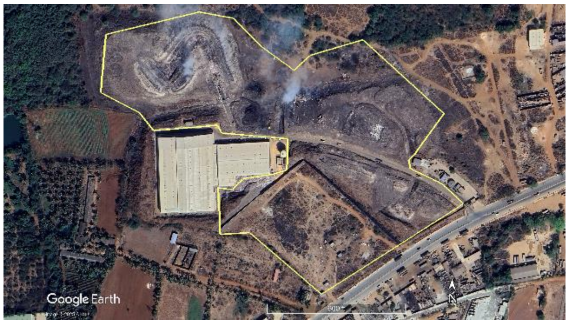

Fig. 85 Google Earth Imagery

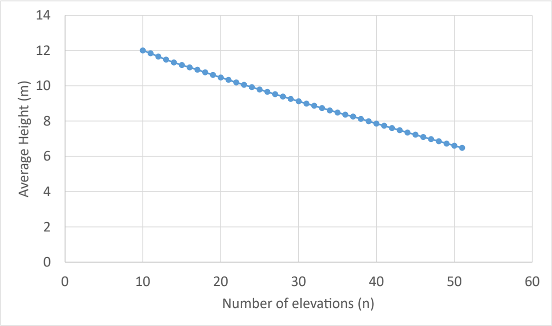

Fig. 86 Waste height (m) variation with change in pixel population (n)

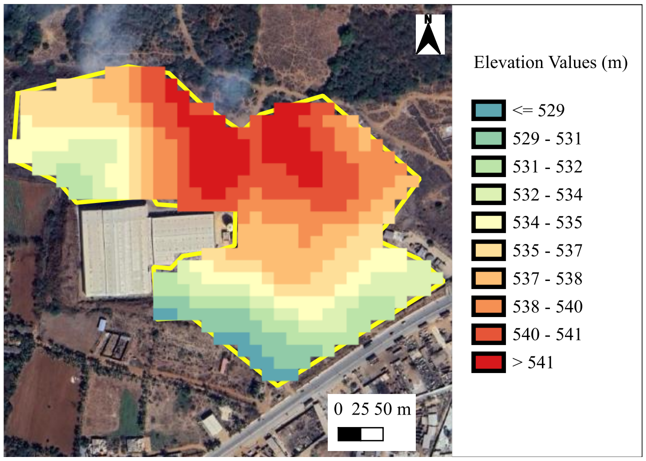

Fig. 87 Landfill elevation variations

Landfill Site 30: Ichalkaranji Landfill

Place: Ichalkaranji, Maharashtra

Location: 16°42'10.28"N, 74°28'52.86"E

Average Waste Height: 2.61 m

Area of Landfill: 22503.31 m^2

Estimated Volume of Waste: 58648.26 m^3

Total Pixel Population within AOI (N): 27

Sentinel-1 Image Dates: 02-Jan-2025 and 26-Jan-2025

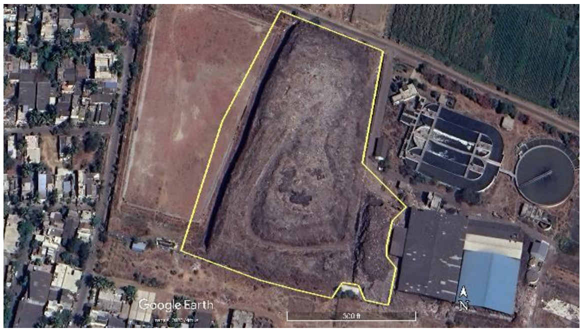

Fig. 88 Google Earth Imagery

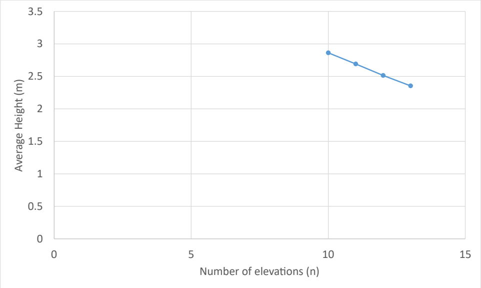

Fig. 89 Waste height (m) variation with change in pixel population (n)

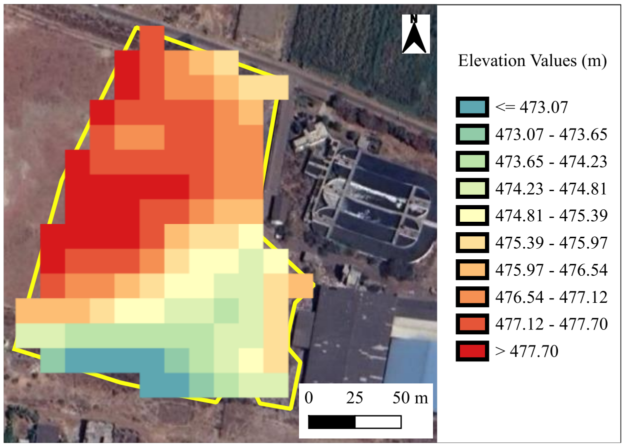

Fig. 90 Landfill elevation variations

Landfill Site 31: Jaipur Landfill 1

Place: Jaipur, Rajasthan

Location: 26°56'46.47"N, 75°55'5.42"E

Average Waste Height: 5.54 m

Area of Landfill: 302028.40 m^2

Estimated Volume of Waste: 1672491.41 m^3

Total Pixel Population within AOI (N): 383

Sentinel-1 Image Dates: 02-Jan-2025 and 26-Jan-2025

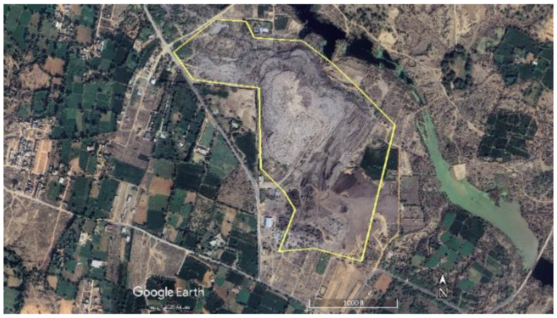

Fig. 91 Google Earth Imagery

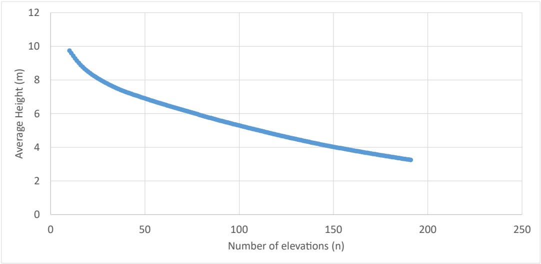

Fig. 92 Waste height (m) variation with change in pixel population (n)

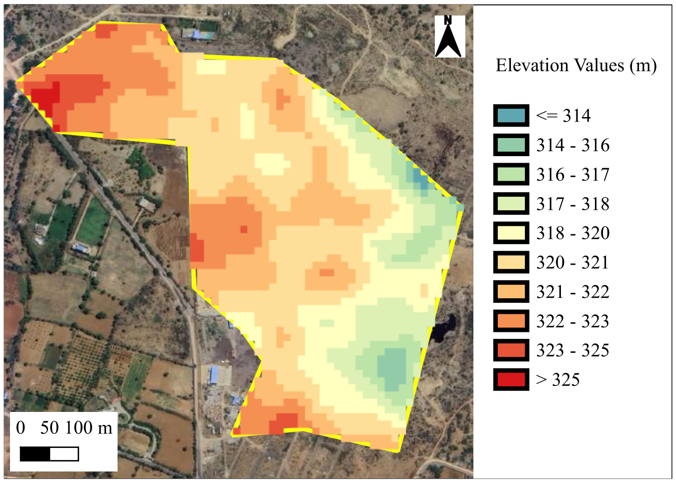

Fig. 93 Landfill elevation variations

Landfill Site 32: Jaipur Landfill 2

Place: Jaipur, Rajasthan

Location: 27° 2'29.85"N, 75°47'58.84"E

Average Waste Height: 9.56 m

Area of Landfill: 196179.15 m^2

Estimated Volume of Waste: 1876046.89 m^3

Total Pixel Population within AOI (N): 246

Sentinel-1 Image Dates: 02-Jan-2025 and 26-Jan-2025

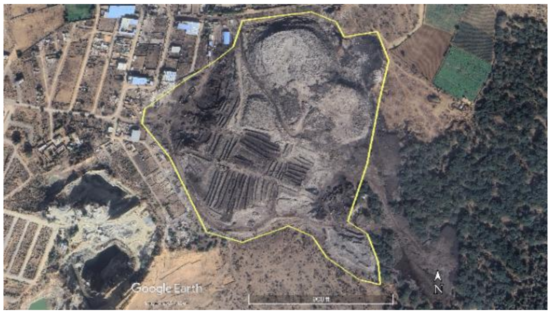

Fig. 94 Google Earth Imagery

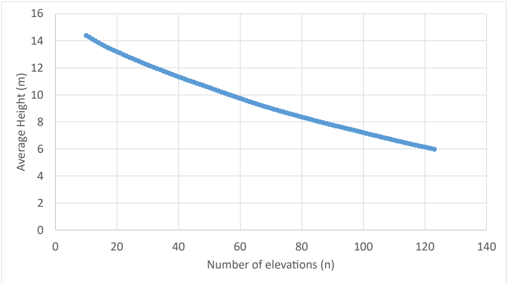

Fig. 95 Waste height (m) variation with change in pixel population (n)

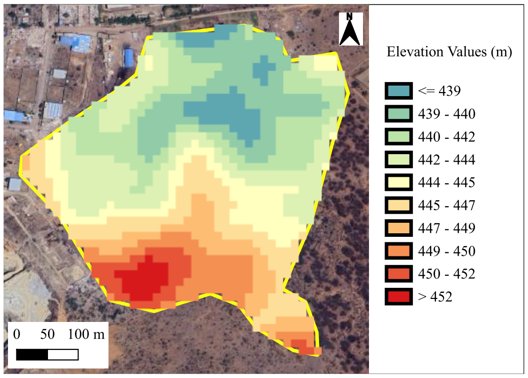

Fig. 96 Landfill elevation variations

Landfill Site 33: Jalore Landfill

Place: Jalore, Rajasthan

Location: 25°21'17.28"N, 72°36'58.82"E

Average Waste Height: 2.44 m

Area of Landfill: 19319.25 m^2

Estimated Volume of Waste: 47083.59 m^3

Total Pixel Population within AOI (N): 23

Sentinel-1 Image Dates: 07-Jan-2025 and 31-Jan-2025

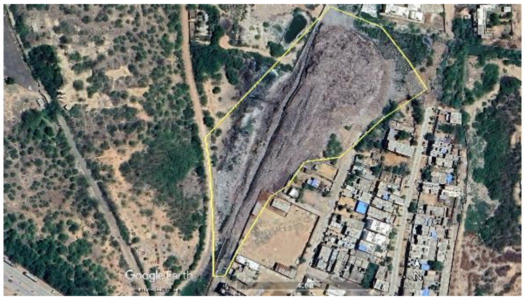

Fig. 97 Google Earth Imagery

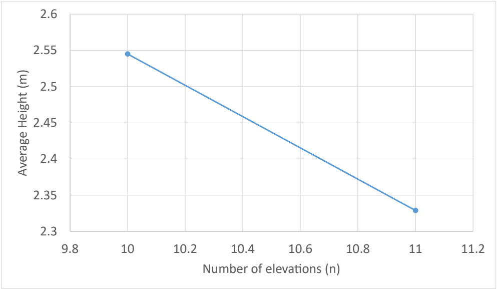

Fig. 98 Waste height (m) variation with change in pixel population (n)

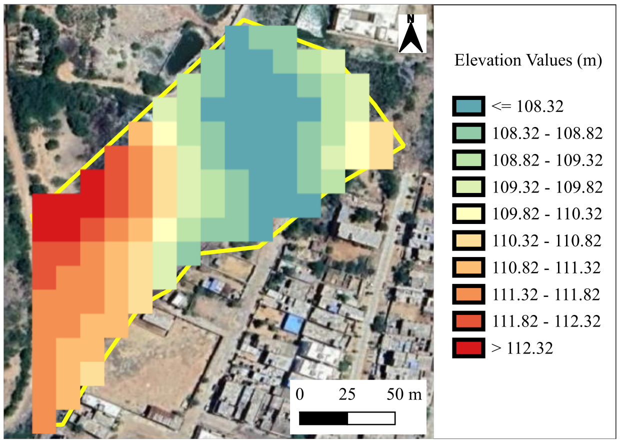

Fig. 99 Landfill elevation variations

Landfill Site 34: Jharkhand Deoghar Landfill

Place: Deoghar, Jharkhand

Location: 24°32'37.88"N, 86°41'52.71"E

Average Waste Height: 2.70 m

Area of Landfill: 35587.78 m^2

Estimated Volume of Waste: 95969.94 m^3

Total Pixel Population within AOI (N): 45

Sentinel-1 Image Dates: 01-Jan-2025 and 25-Jan-2025

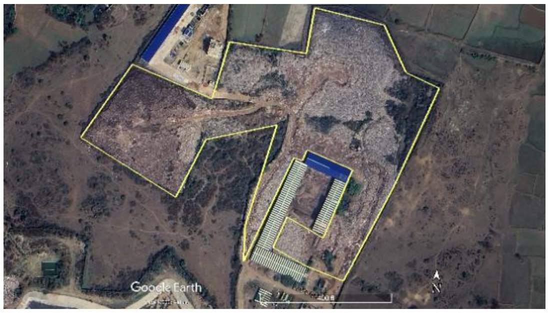

Fig. 100 Google Earth Imagery

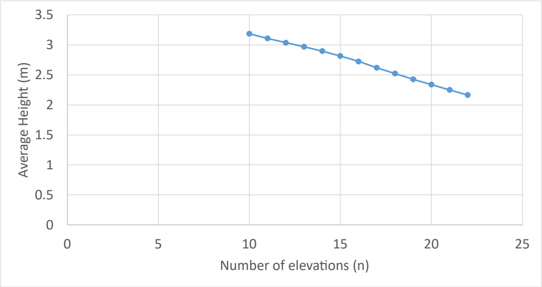

Fig. 101 Waste height (m) variation with change in pixel population (n)

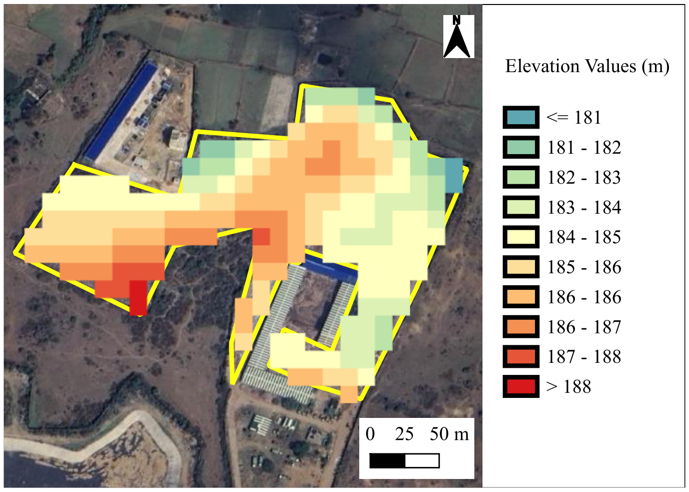

Fig. 102 Landfill elevation variations

Landfill Site 35: Jharkhand Ranchi Landfill

Place: Ranchi, Jharkhand

Location: 23°24'40.11"N, 85°15'18.10"E

Average Waste Height: 19.17 m

Area of Landfill: 141846.52 m^2

Estimated Volume of Waste: 2719443.66 m^3

Total Pixel Population within AOI (N): 171

Sentinel-1 Image Dates: 01-Jan-2025 and 25-Jan-2025

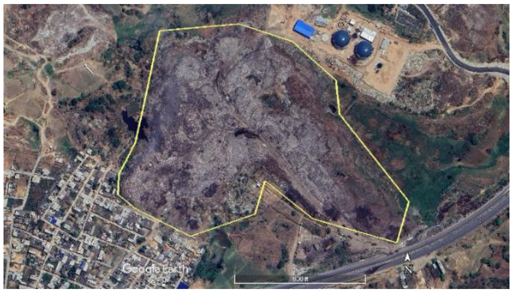

Fig. 103 Google Earth Imagery

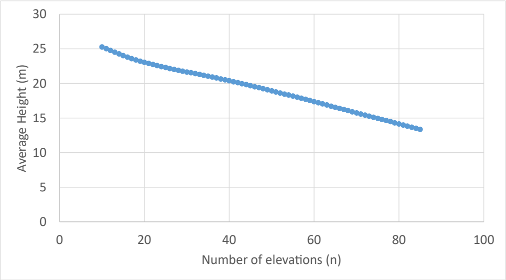

Fig. 104 Waste height (m) variation with change in pixel population (n)

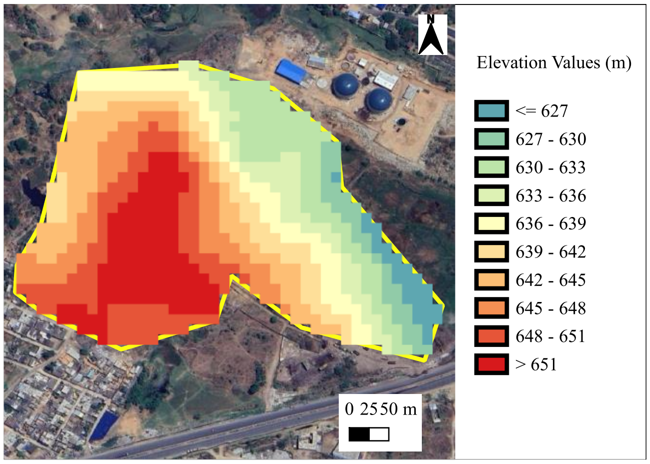

Fig. 105 Landfill elevation variations

Landfill Site 36: Jharkhand Shahpur Landfill

Place: Shahpur, Jharkhand

Location: 24° 2'31.11"N, 84° 2'41.52"E

Average Waste Height: 2.67 m

Area of Landfill: 17745.46 m^2

Estimated Volume of Waste: 47448.32 m^3

Total Pixel Population within AOI (N): 23

Sentinel-1 Image Dates: 06-Jan-2025 and 30-Jan-2025

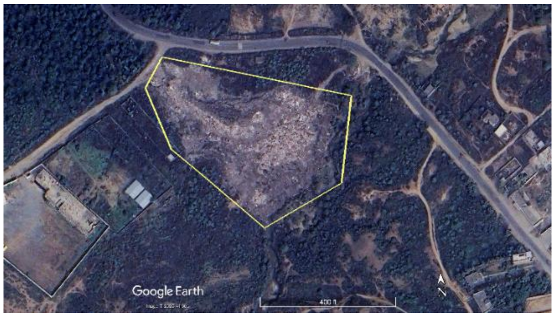

Fig. 106 Google Earth Imagery

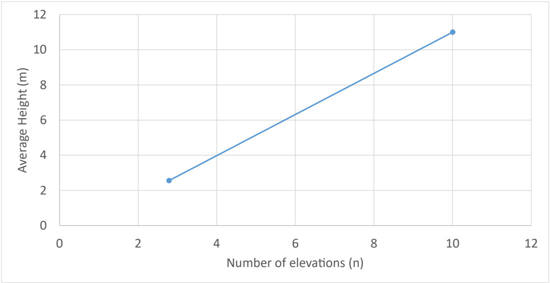

Fig. 107 Waste height (m) variation with change in pixel population (n)

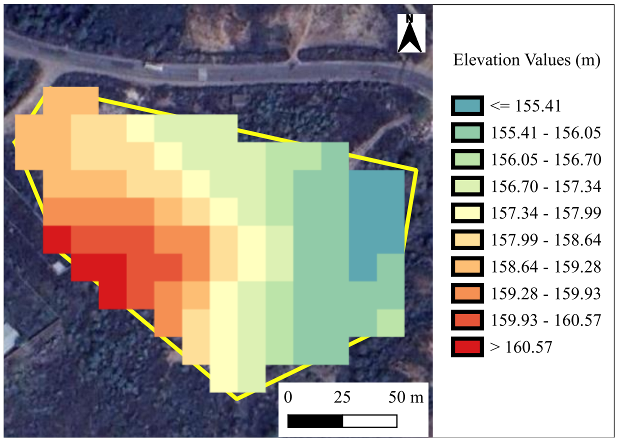

Fig. 108 Landfill elevation variations

Landfill Site 37: Jharkhand Sindri Landfill

Place: Sindri, Jharkhand

Location: 23°38'37.94"N, 86°29'1.02"E

Average Waste Height: 2.32 m

Area of Landfill: 60153.63 m^2

Estimated Volume of Waste: 139571.69 m^3

Total Pixel Population within AOI (N): 75

Sentinel-1 Image Dates: 01-Jan-2025 and 25-Jan-2025

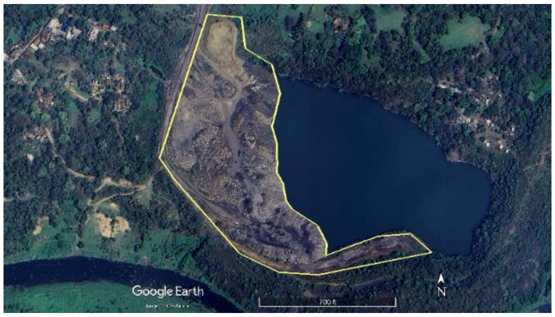

Fig. 109 Google Earth Imagery

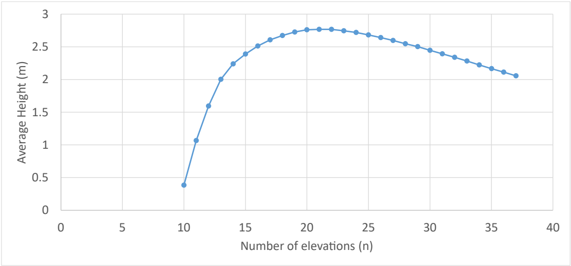

Fig. 110 Waste height (m) variation with change in pixel population (n)

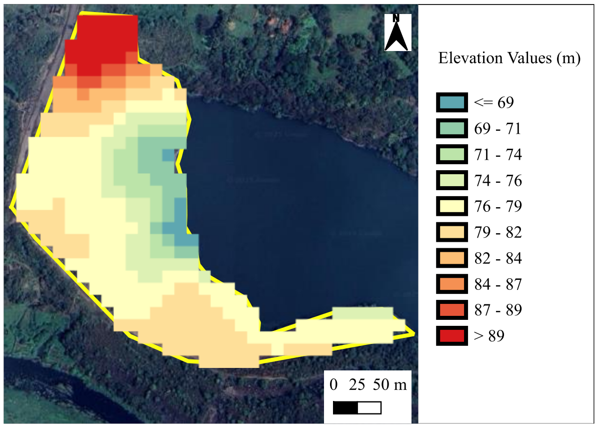

Fig. 111 Landfill elevation variations

Landfill Site 38: Kanpur Pankabahadur Nagar Municipal Landfill

Place: Kanpur, Uttar Pradesh

Location: 26°26'55.11"N, 80°14'3.86"E

Average Waste Height: 4.86 m

Area of Landfill: 266275.98 m^2

Estimated Volume of Waste: 1292969.16 m^3

Total Pixel Population within AOI (N): 334

Sentinel-1 Image Dates: 04-Jan-2025 and 28-Jan-2025

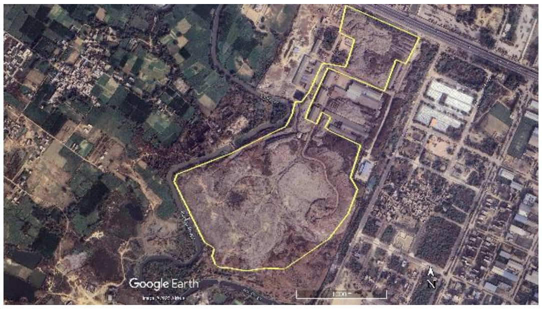

Fig. 112 Google Earth Imagery

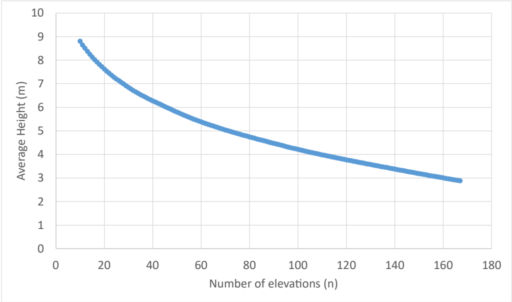

Fig. 113 Waste height (m) variation with change in pixel population (n)

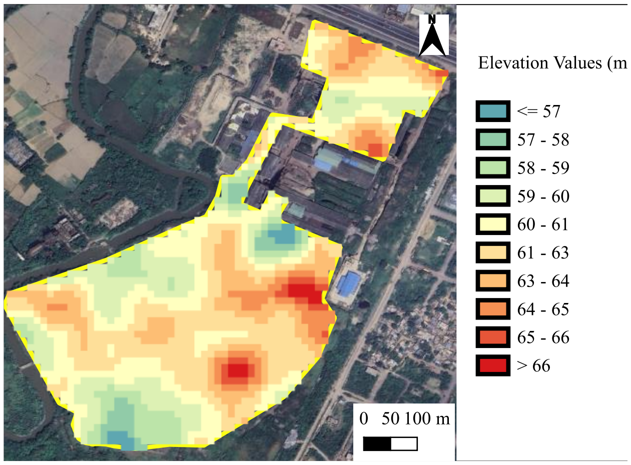

Fig. 114 Landfill elevation variations

Landfill Site 39: Kerela Brahmapuram Dumping Ground

Place: Ernakulam, Kerela

Location: 9°59'30.32"N, 76°21'53.31"E

Average Waste Height: 30.38 m

Area of Landfill: 134220.37 m^2

Estimated Volume of Waste: 4078147.29 m^3

Total Pixel Population within AOI (N): 153

Sentinel-1 Image Dates: 04-Jan-2025 and 28-Jan-2025

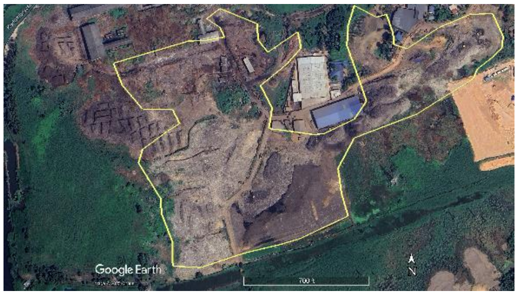

Fig. 115 Google Earth Imagery

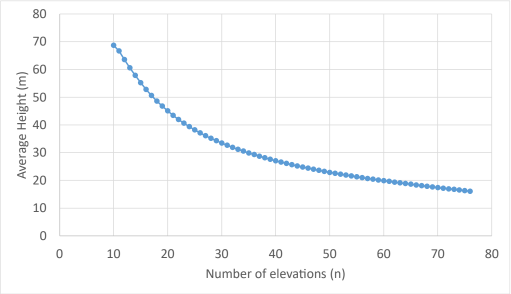

Fig. 116 Waste height (m) variation with change in pixel population (n)

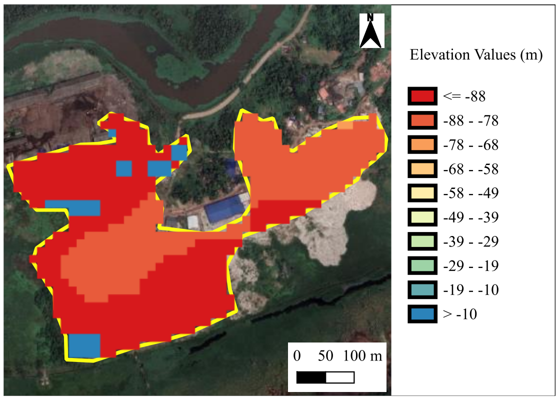

Fig. 117 Landfill elevation variations

Landfill Site 40: Khargone Landfill

Place: Khargone, Madhya Pradesh

Location: 21°48'10.03"N, 75°38'10.64"E

Average Waste Height: 5.42 m

Area of Landfill: 70407.61 m^2

Estimated Volume of Waste: 381365.71 m^3

Total Pixel Population within AOI (N): 91

Sentinel-1 Image Dates: 02-Jan-2025 and 26-Jan-2025

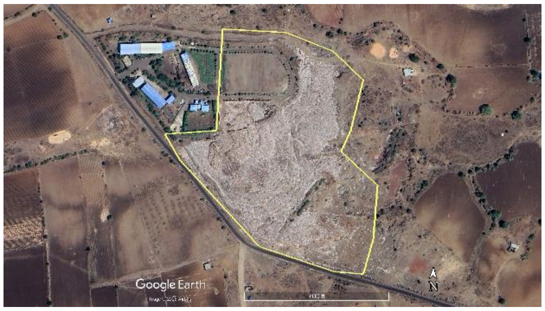

Fig. 118 Google Earth Imagery

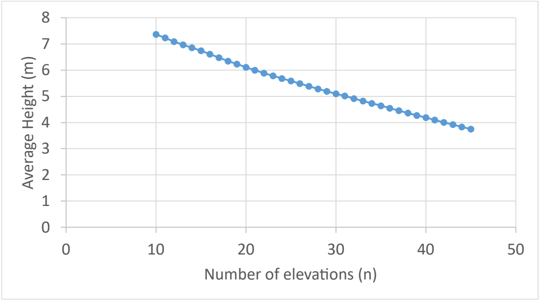

Fig. 119 Waste height (m) variation with change in pixel population (n)

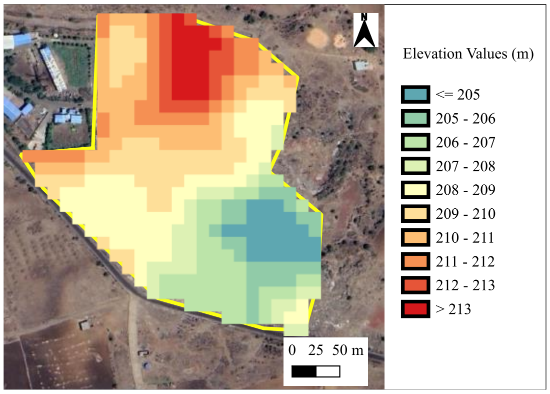

Fig. 120 Landfill elevation variations

Landfill Site 41: Kolhapur Landfill

Place: Kolhapur, Maharashtra

Location: 16°43'29.64"N, 74°14'58.42"E

Average Waste Height: 4.64 m

Area of Landfill: 90358.82 m^2

Estimated Volume of Waste: 419200.75 m^3

Total Pixel Population within AOI (N): 107

Sentinel-1 Image Dates: 02-Jan-2025 and 26-Jan-2025

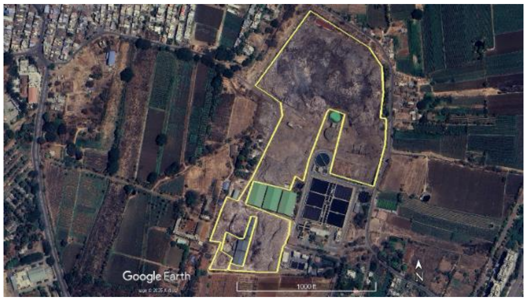

Fig. 121 Google Earth Imagery

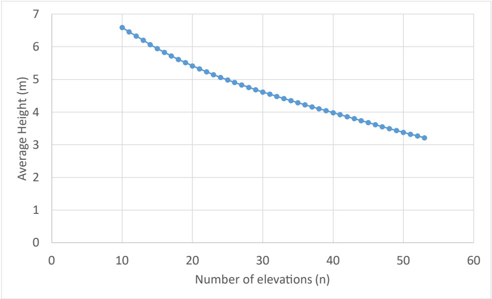

Fig. 122 Waste height (m) variation with change in pixel population (n)

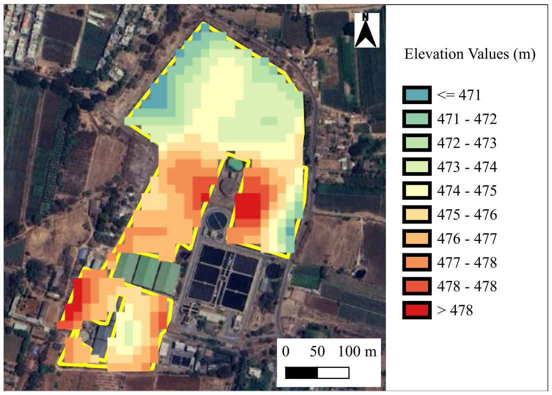

Fig. 123 Landfill elevation variations

Landfill Site 42: Kolkatta Dhapa Garbage Dumping Ground

Place: Kolkata, West Bengal

Location: 22°32'13.85"N, 88°25'28.74"E

Average Waste Height: 5.22 m

Area of Landfill: 482911.19 m^2

Estimated Volume of Waste: 2522007.78 m^3

Total Pixel Population within AOI (N): 576

Sentinel-1 Image Dates: 05-Jan-2025 and 29-Jan-2025

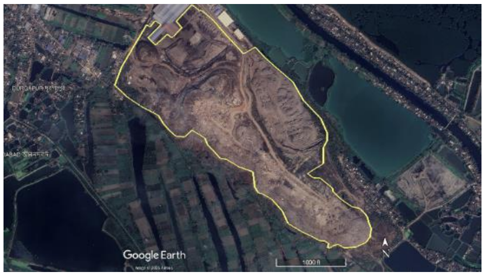

Fig. 124 Google Earth Imagery

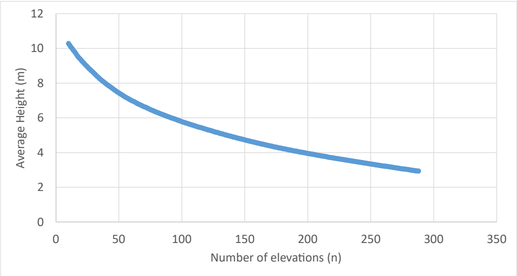

Fig. 125 Waste height (m) variation with change in pixel population (n)

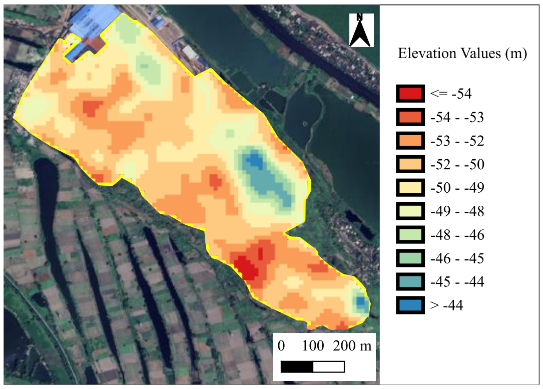

Fig. 126 Landfill elevation variations

Landfill Site 43: Latur Landfill

Place: Latur, Maharashtra

Location: 18°26'13.69"N, 76°32'17.82"E

Average Waste Height: 3.65 m

Area of Landfill: 52122.71 m^2

Estimated Volume of Waste: 190219.41 m^3

Total Pixel Population within AOI (N): 61

Sentinel-1 Image Dates: 09-Jan-2025 and 21-Jan-2025

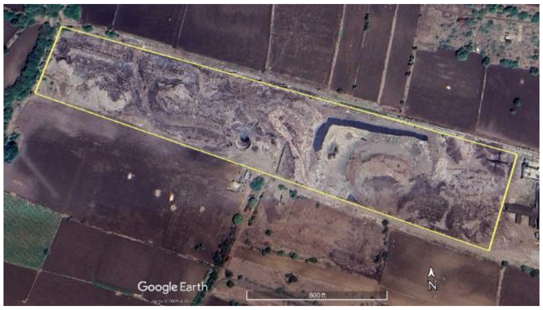

Fig. 127 Google Earth Imagery

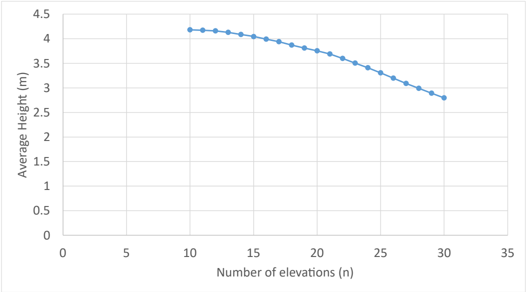

Fig. 128 Waste height (m) variation with change in pixel population (n)

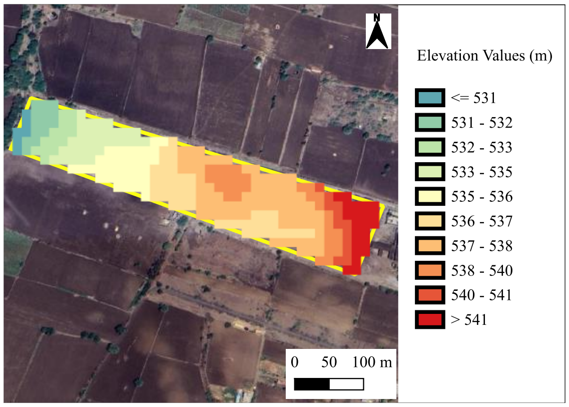

Fig. 129 Landfill elevation variations

Landfill Site 44: MBMC Dumping Ground

Place: Mumbai, Maharashtra

Location: 19°17'15.88"N, 72°47'38.54"E

Average Waste Height: 37.05 m

Area of Landfill: 88005.24 m^2

Estimated Volume of Waste: 3260420.33 m^3

Total Pixel Population within AOI (N): 104

Sentinel-1 Image Dates: 07-Jan-2025 and 31-Jan-2025

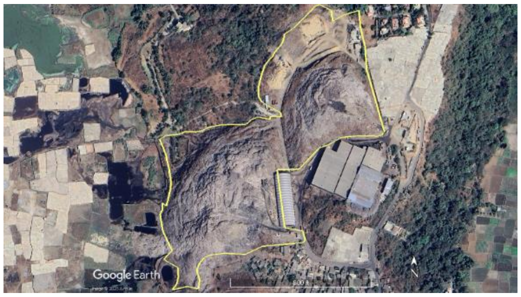

Fig. 130 Google Earth Imagery

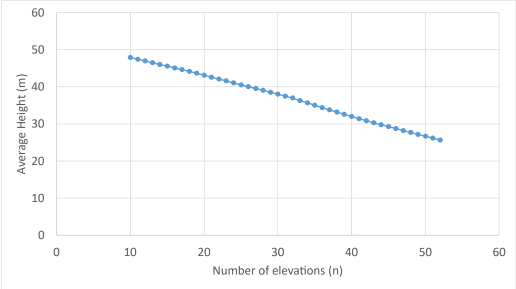

Fig. 131 Waste height (m) variation with change in pixel population (n)

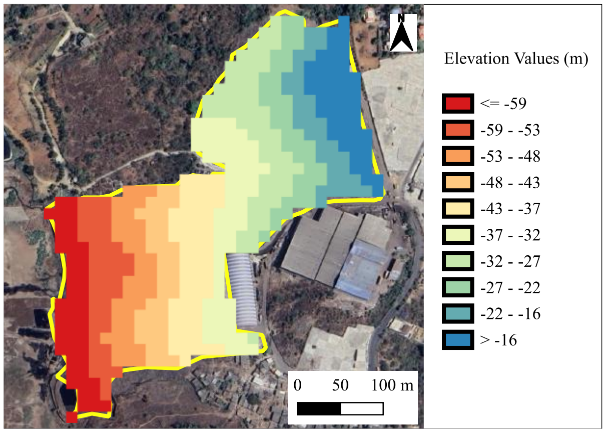

Fig. 132 Landfill elevation variations

Landfill Site 45: Meerut Landfill

Place: Meerut, Uttar Pradesh

Location: 28°55'35.58"N, 77°43'57.27"E

Average Waste Height: 4.61 m

Area of Landfill: 114968.91 m^2

Estimated Volume of Waste: 530471.12 m^3

Total Pixel Population within AOI (N): 147

Sentinel-1 Image Dates: 02-Jan-2025 and 26-Jan-2025

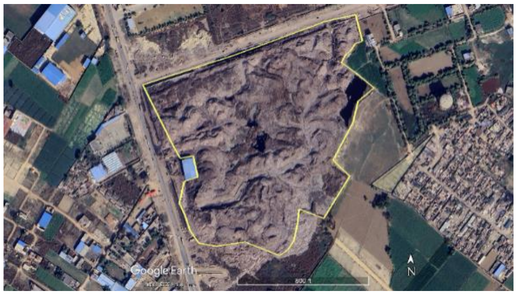

Fig. 133 Google Earth Imagery

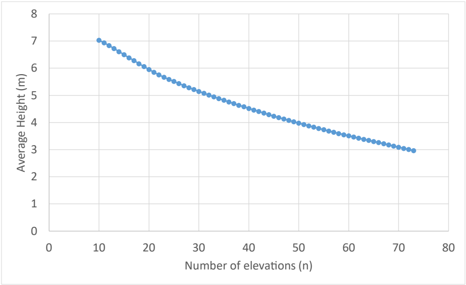

Fig. 134 Waste height (m) variation with change in pixel population (n)

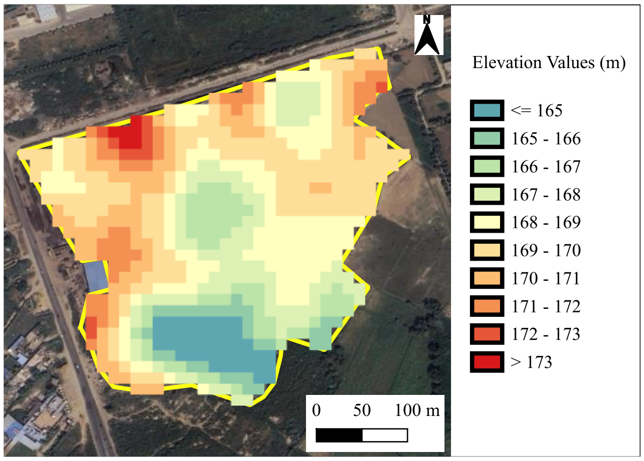

Fig. 135 Landfill elevation variations

Landfill Site 46: Meghalaya Tura Landfill

Place: Tura, Meghalaya

Location: 25°31'50.30"N, 90°11'16.93"E

Average Waste Height: 0.58 m

Area of Landfill: 10070.63 m^2

Estimated Volume of Waste: 5834.28 m^3

Total Pixel Population within AOI (N): 14

Sentinel-1 Image Dates: 02-Jan-2025 and 26-Jan-2025

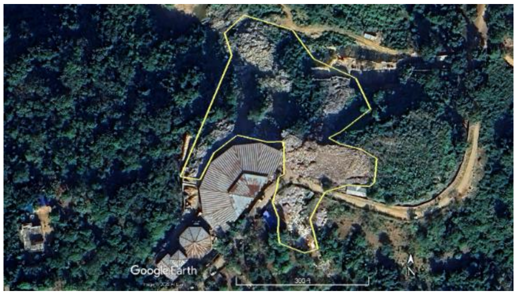

Fig. 136 Google Earth Imagery

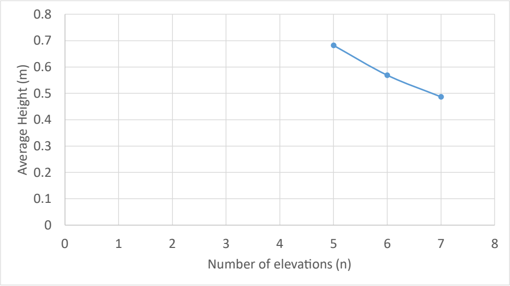

Fig. 137 Waste height (m) variation with change in pixel population (n)

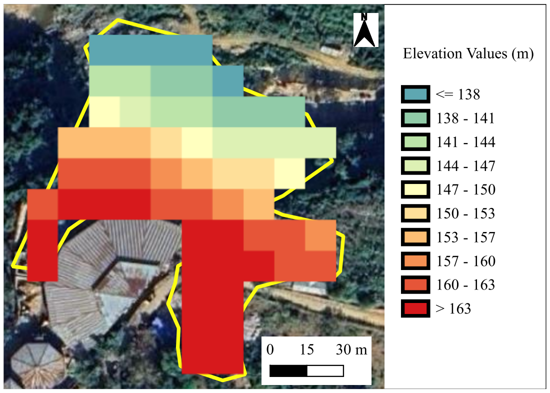

Fig. 138 Landfill elevation variations

Landfill Site 47: Miyati Landfill

Place: Miyati, Madhya Pradesh

Location: 23° 4'22.27"N, 74°33'40.57"E

Average Waste Height: 1.93 m

Area of Landfill: 25229.77 m^2

Estimated Volume of Waste: 48723.98 m^3

Total Pixel Population within AOI (N): 30

Sentinel-1 Image Dates: 07-Jan-2025 and 31-Jan-2025

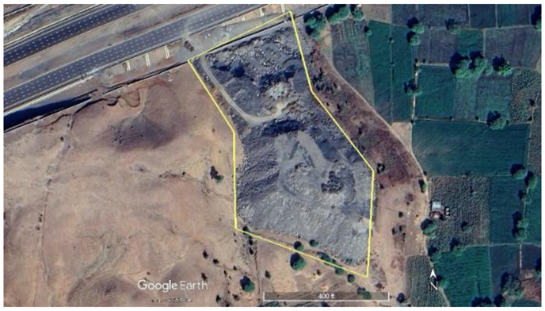

Fig. 139 Google Earth Imagery

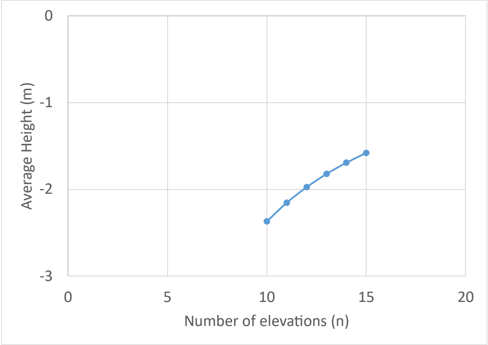

Fig. 140 Waste height (m) variation with change in pixel population (n)

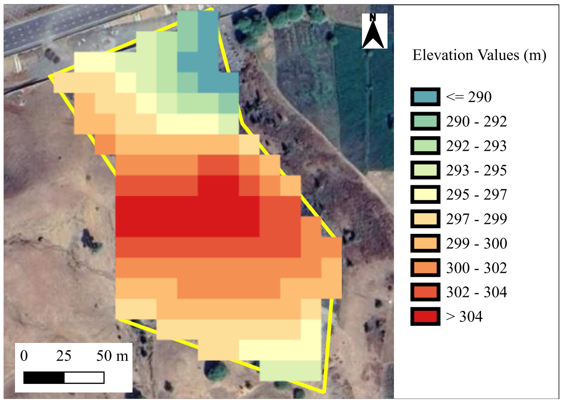

Fig. 141 Landfill elevation variations

Landfill Site 48: Mumbai Kalyan Landfill

Place: Mumbai, Maharashtra

Location: 19°18'28.28"N, 73° 4'57.50"E

Average Waste Height: 11.33 m

Area of Landfill: 64824.91 m^2

Estimated Volume of Waste: 734649.26 m^3

Total Pixel Population within AOI (N): 77

Sentinel-1 Image Dates: 07-Jan-2025 and 31-Jan-2025

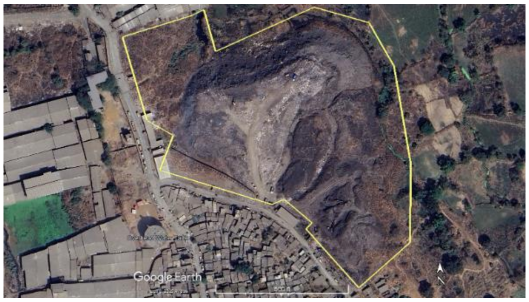

Fig. 142 Google Earth Imagery

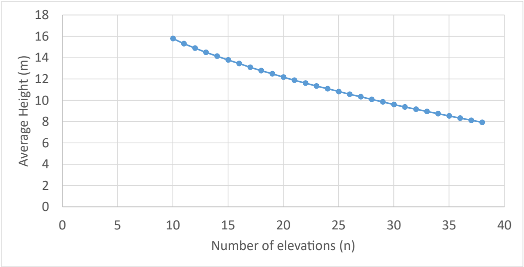

Fig. 143 Waste height (m) variation with change in pixel population (n)

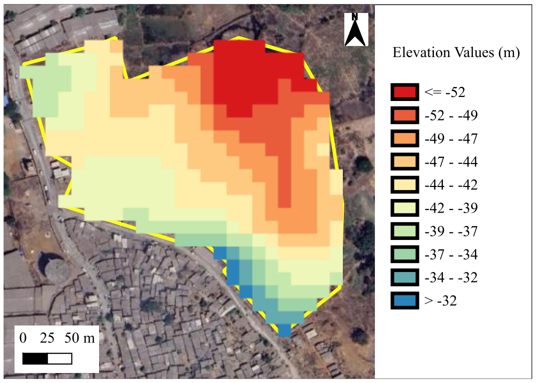

Fig. 144 Landfill elevation variations

Landfill Site 49: Mumbai KDMC Landfill

Place: Mumbai, Maharashtra

Location: 19°16'37.77"N, 73° 7'26.68"E

Average Waste Height: 1.88 m

Area of Landfill: 64406.42 m^2

Estimated Volume of Waste: 120797.00 m^3

Total Pixel Population within AOI (N): 77

Sentinel-1 Image Dates: 07-Jan-2025 and 31-Jan-2025

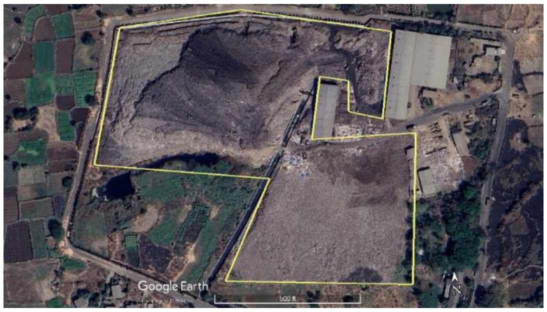

Fig. 145 Google Earth Imagery

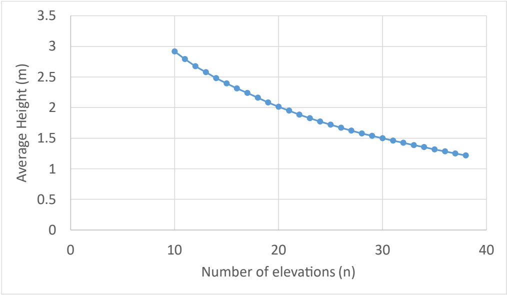

Fig. 146 Waste height (m) variation with change in pixel population (n)

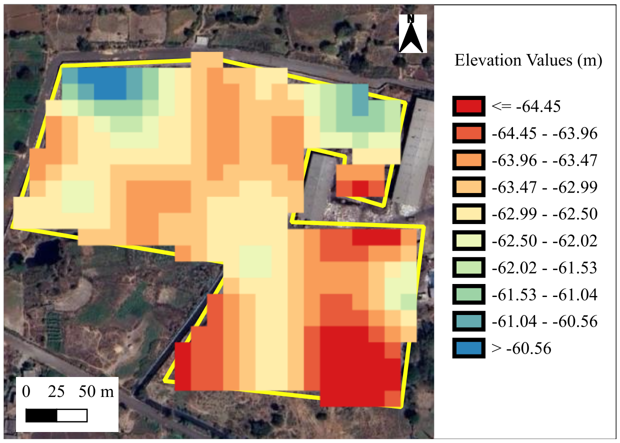

Fig. 147 Landfill elevation variations

Landfill Site 50: Mumbai VVMC Landfill

Place: Mumbai, Maharashtra

Location: 19°24'5.52"N, 72°51'35.24"E

Average Waste Height: 10.85 m

Area of Landfill: 85194.36 m^2

Estimated Volume of Waste: 924400.12 m^3

Total Pixel Population within AOI (N): 98

Sentinel-1 Image Dates: 07-Jan-2025 and 31-Jan-2025

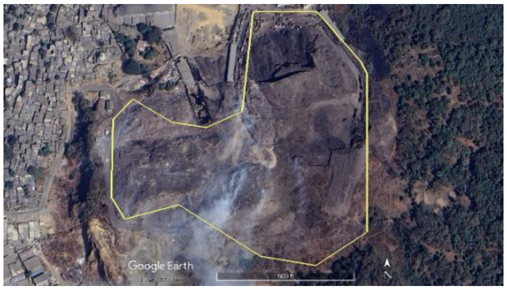

Fig. 148 Google Earth Imagery

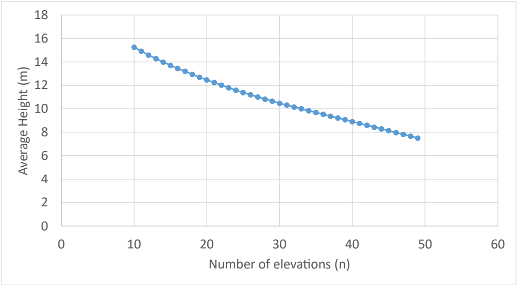

Fig. 149 Waste height (m) variation with change in pixel population (n)

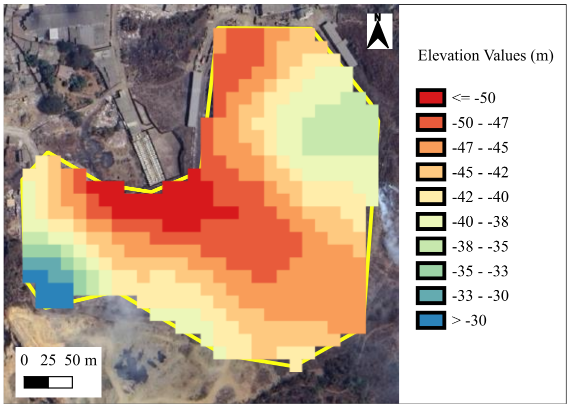

Fig. 150 Landfill elevation variations

Landfill Site 51: Muradnagar Landfill

Place: Muradnagar, Uttar Pradesh

Location: 28°46'24.52"N, 77°31'9.38"E

Average Waste Height: 4.96 m

Area of Landfill: 38744.08 m^2

Estimated Volume of Waste: 192326.13 m^3

Total Pixel Population within AOI (N): 49

Sentinel-1 Image Dates: 02-Jan-2025 and 26-Jan-2025

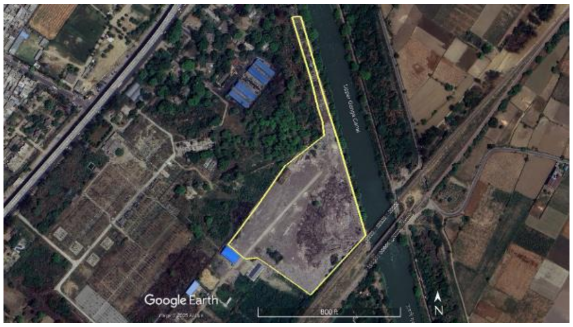

Fig. 151 Google Earth Imagery

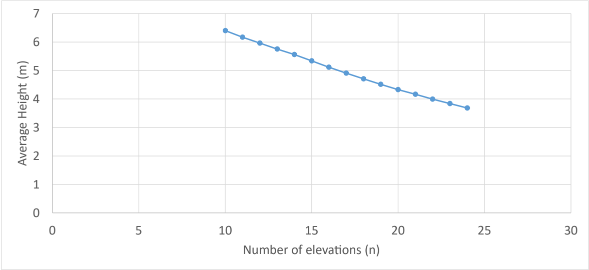

Fig. 152 Waste height (m) variation with change in pixel population (n)

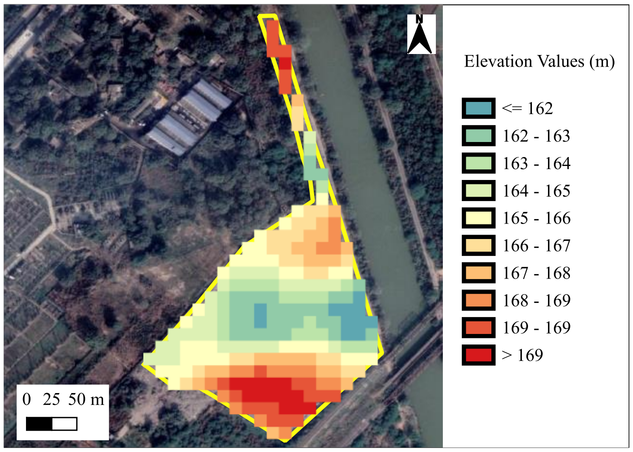

Fig. 153 Landfill elevation variations

Landfill Site 52: Mysuru Landfill Site

Place: Mysuru, Karnataka

Location: 12°16'7.22"N, 76°39'16.96"E

Average Waste Height: 11.04 m

Area of Landfill: 145606.90 m^2

Estimated Volume of Waste: 1608044.99m^3

Total Pixel Population within AOI (N): 168

Sentinel-1 Image Dates: 04-Jan-2025 and 28-Jan-2025

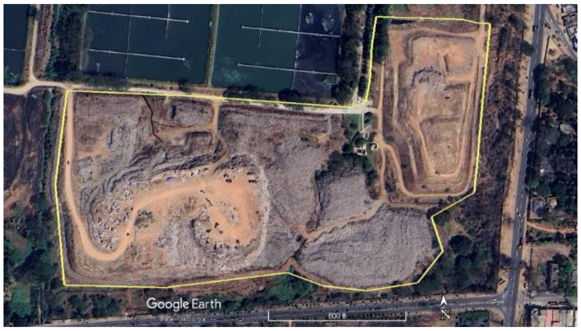

Fig. 154 Google Earth Imagery

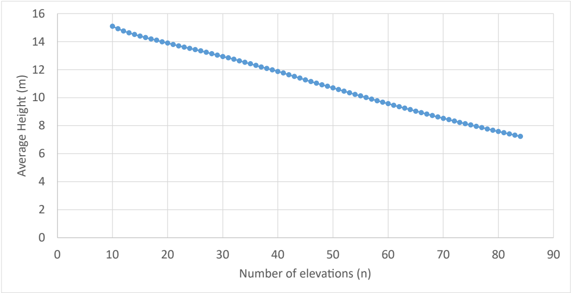

Fig. 155 Waste height (m) variation with change in pixel population (n)

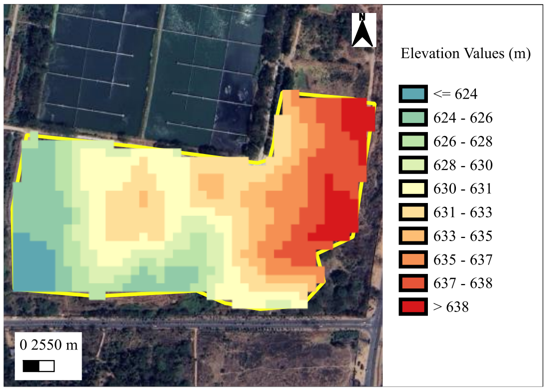

Fig. 156 Landfill elevation variations

Landfill Site 53: Nagpur Landfill

Place: Nagpur, Maharashtra

Location: 21° 8'30.09"N, 79° 9'8.74"E

Average Waste Height: 4.58 m

Area of Landfill: 125860.33 m^2

Estimated Volume of Waste: 575960.36 m^3

Total Pixel Population within AOI (N): 151

Sentinel-1 Image Dates: 04-Jan-2025 and 28-Jan-2025

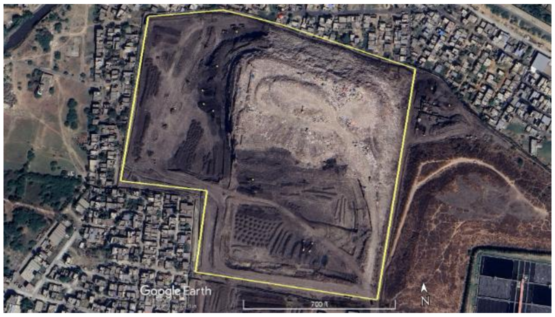

Fig. 157 Google Earth Imagery

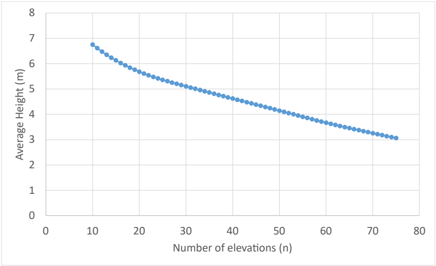

Fig. 158 Waste height (m) variation with change in pixel population (n)

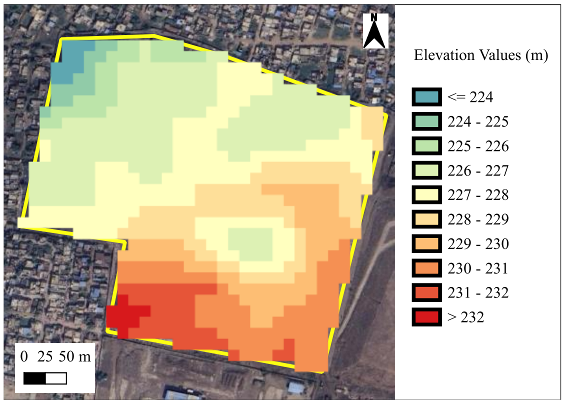

Fig. 159 Landfill elevation variations

Landfill Site 54: Naregaon Landfill

Place: Naregaon, Maharashtra

Location: 19°53'32.67"N, 75°23'54.46"E

Average Waste Height: 3.97 m

Area of Landfill: 179894.93 m^2

Estimated Volume of Waste: 713286.51 m^3

Total Pixel Population within AOI (N): 209

Sentinel-1 Image Dates: 02-Jan-2025 and 26-Jan-2025

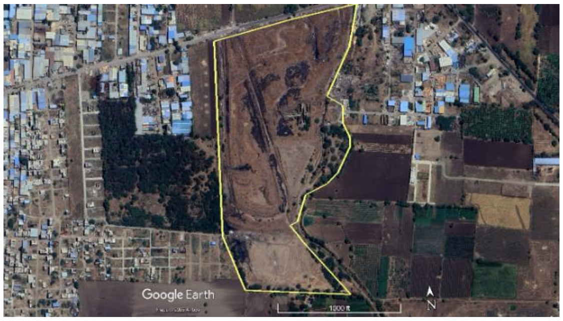

Fig. 160 Google Earth Imagery

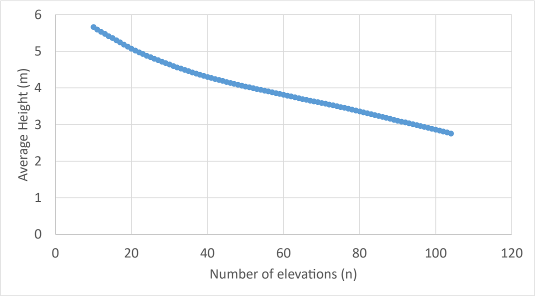

Fig. 161 Waste height (m) variation with change in pixel population (n)

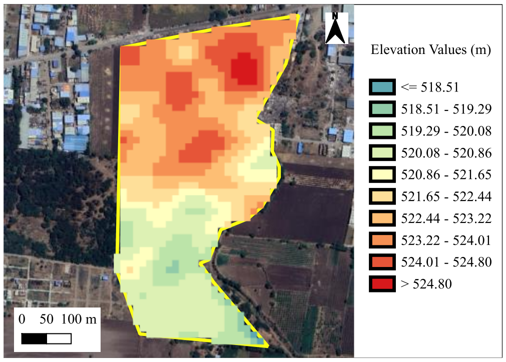

Fig. 162 Landfill elevation variations

Landfill Site 55: Narela Bawana Landfill

Place: New Delhi

Location: 28°48'7.30"N, 77° 4'6.39"E

Average Waste Height: 4.35 m

Area of Landfill: 143659.72 m^2

Estimated Volume of Waste: 624711.58 m^3

Total Pixel Population within AOI (N): 176

Sentinel-1 Image Dates: 02-Jan-2025 and 26-Jan-2025

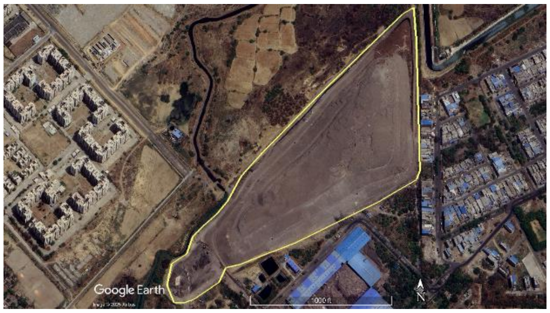

Fig. 163 Google Earth Imagery

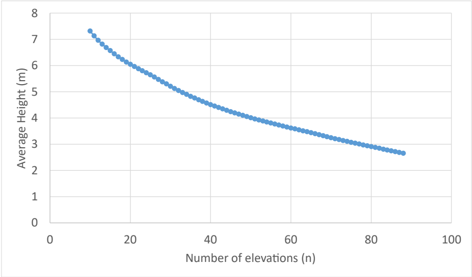

Fig. 164 Waste height (m) variation with change in pixel population (n)

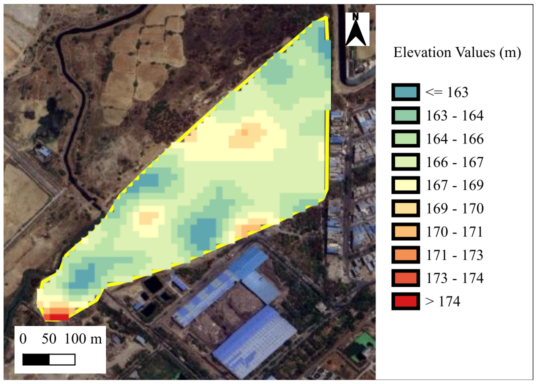

Fig. 165 Landfill elevation variations

Landfill Site 56: Near Mangaluru Landfill

Place: Mangluru, Karnataka

Location: 12°54'36.13"N, 74°53'24.85"E

Average Waste Height: 0.66 m

Area of Landfill: 50464.96 m^2

Estimated Volume of Waste: 33261.31 m^3

Total Pixel Population within AOI (N): 57

Sentinel-1 Image Dates: 09-Jan-2025 and 21-Jan-2025

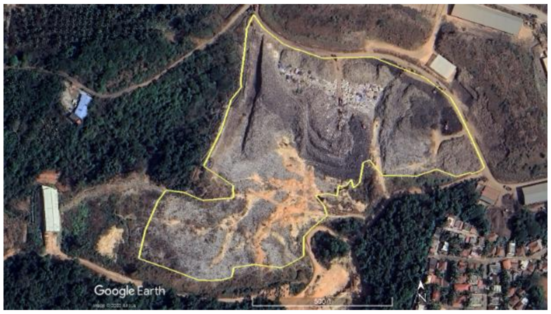

Fig. 166 Google Earth Imagery

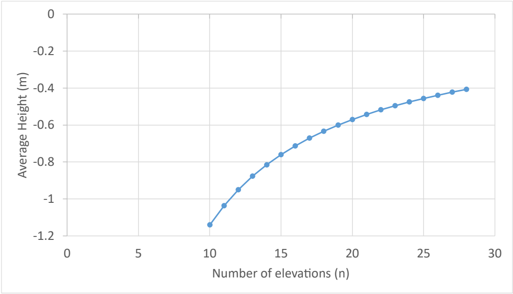

Fig. 167 Waste height (m) variation with change in pixel population (n)

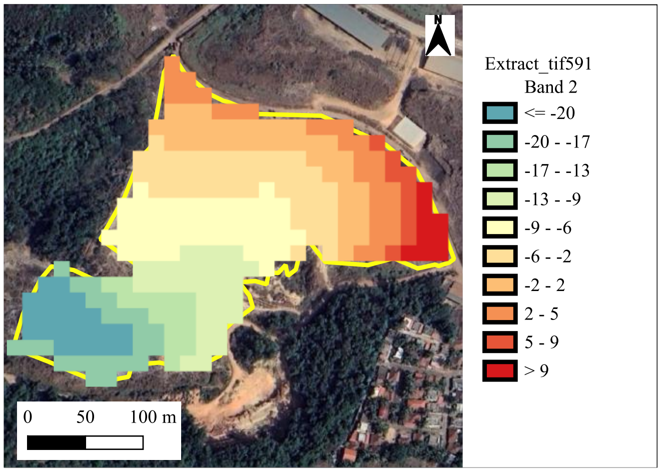

Fig. 168 Landfill elevation variations

Landfill Site 57: Odihsa Bhubaneswar Landfill 3

Place: Bhubaneshwar, Odisha

Location: 20°24'52.58"N, 85°46'34.34"E

Average Waste Height: 6.33 m

Area of Landfill: 80809.99 m^2

Estimated Volume of Waste: 511737.90 m^3

Total Pixel Population within AOI (N): 95

Sentinel-1 Image Dates: 01-Jan-2025 and 25-Jan-2025

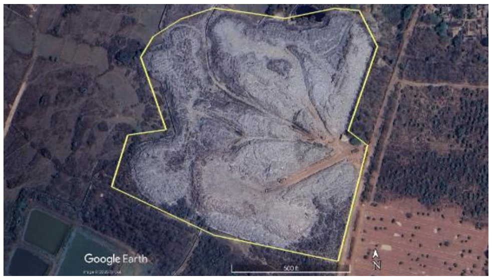

Fig. 169 Google Earth Imagery

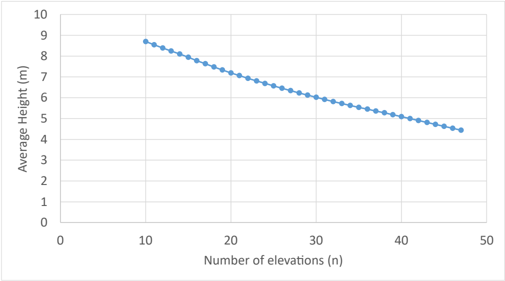

Fig. 170 Waste height (m) variation with change in pixel population (n)

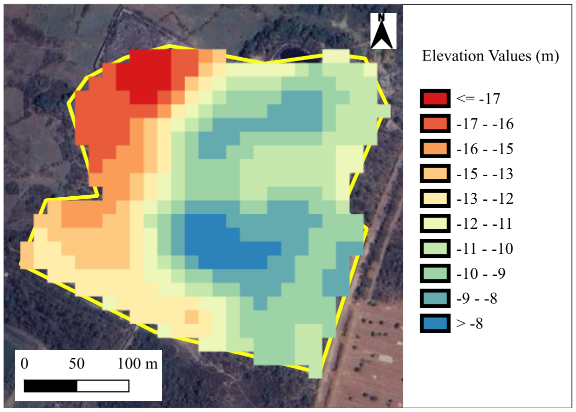

Fig. 171 Landfill elevation variations

Landfill Site 58: Odisha Bhubaneswar Landfill

Place: Bhubaneshwar, Odisha

Location: 20°18'58.21"N, 85°50'23.23"E

Average Waste Height: 4.67 m

Area of Landfill: 33393.50 m^2

Estimated Volume of Waste: 155933.65 m^3

Total Pixel Population within AOI (N): 45

Sentinel-1 Image Dates: 01-Jan-2025 and 25-Jan-2025

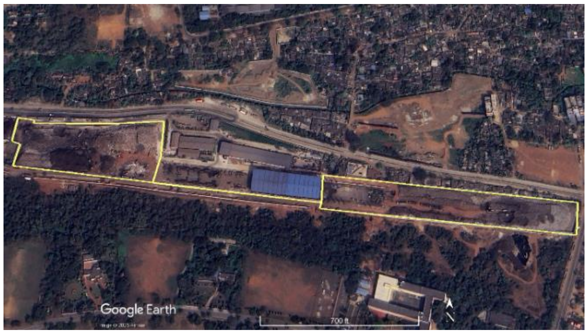

Fig. 172 Google Earth Imagery

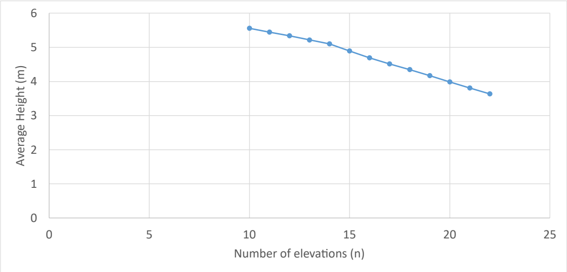

Fig. 173 Waste height (m) variation with change in pixel population (n)

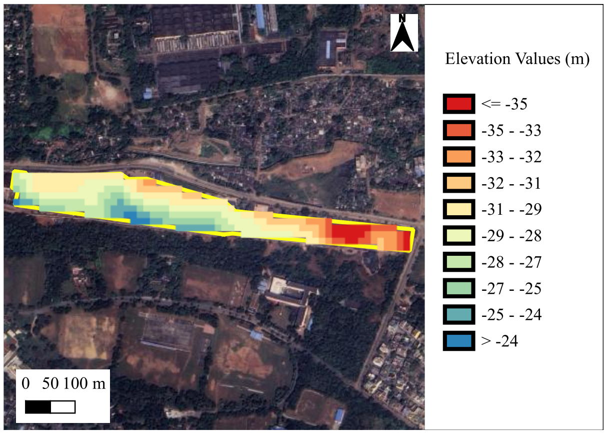

Fig. 174 Landfill elevation variations

Landfill Site 59: Odisha Bhubaneswar Landfill 2

Place: Bhubaneshwar, Odisha

Location: 20°23'28.98"N, 85°47'12.41"E

Average Waste Height: 11.64 m

Area of Landfill: 234482.79 m^2

Estimated Volume of Waste: 2730507.84 m^3

Total Pixel Population within AOI (N): 282

Sentinel-1 Image Dates: 01-Jan-2025 and 25-Jan-2025

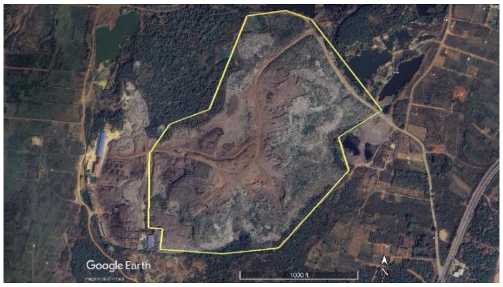

Fig. 175 Google Earth Imagery

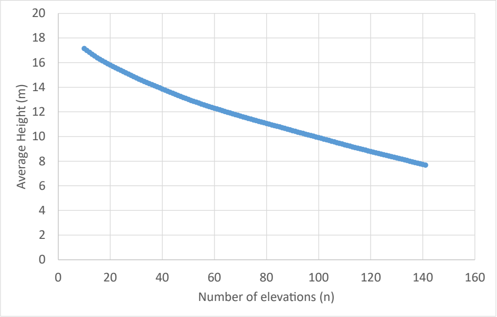

Fig. 176 Waste height (m) variation with change in pixel population (n)

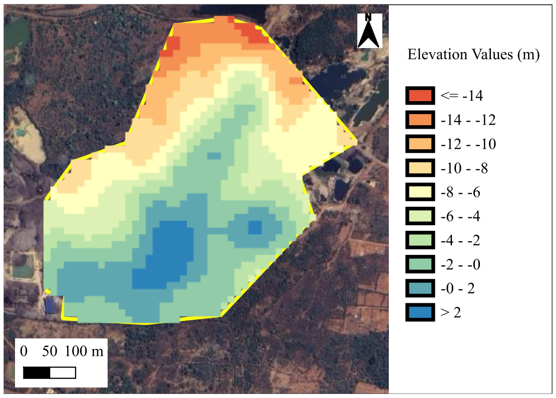

Fig. 177 Landfill elevation variations

Landfill Site 60: Okhla Landfill

Place: New Delhi

Location: 28°30'41.98"N, 77°17'3.44"E

Average Waste Height: 20.81 m

Area of Landfill: 229504.68 m^2

Estimated Volume of Waste: 4774996.47 m^3

Total Pixel Population within AOI (N): 292

Sentinel-1 Image Dates: 02-Jan-2025 and 26-Jan-2025

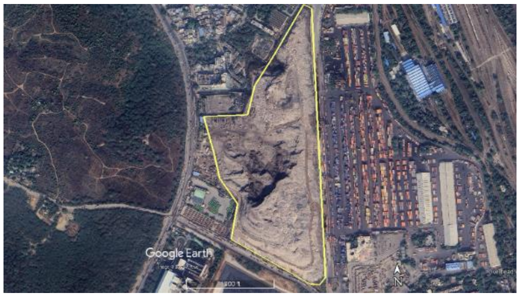

Fig. 178 Google Earth Imagery

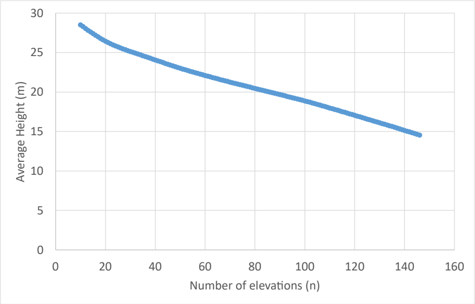

Fig. 179 Waste height (m) variation with change in pixel population (n)

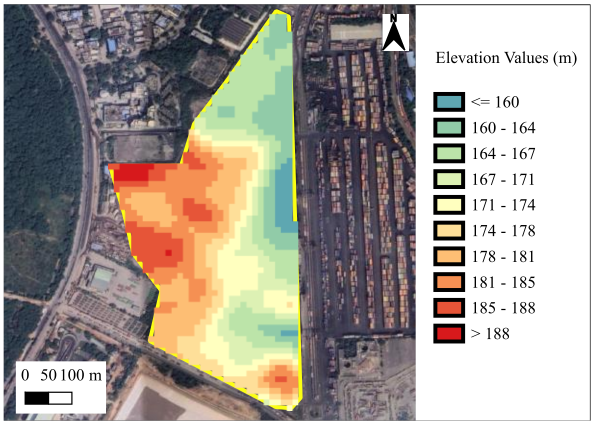

Fig. 180 Landfill elevation variations

Landfill Site 61: Pune Landfill

Place: Pune, Maharashtra

Location: 18°39'23.19"N, 73°51'26.64"E

Average Waste Height: 0.63 m

Area of Landfill: 59700.16 m^2

Estimated Volume of Waste: 37847.52 m^3

Total Pixel Population within AOI (N): 69

Sentinel-1 Image Dates: 02-Jan-2025 and 26-Jan-2025

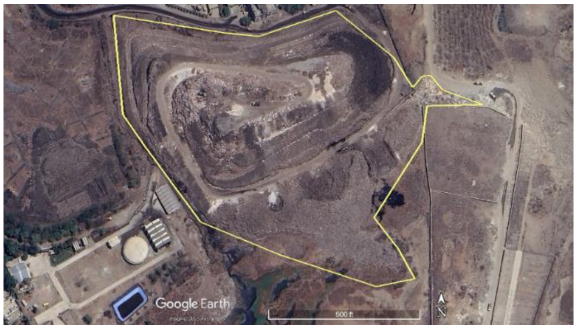

Fig. 181 Google Earth Imagery

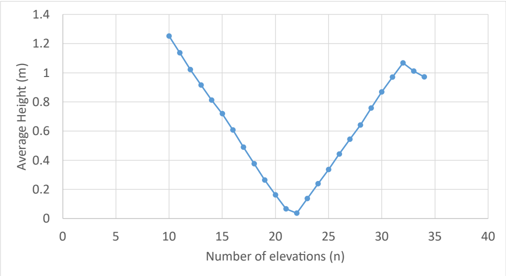

Fig. 182 Waste height (m) variation with change in pixel population (n)

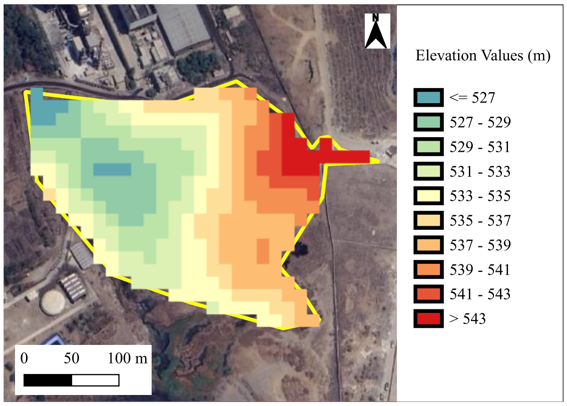

Fig. 183 Landfill elevation variations

Landfill Site 62: Pune Landfill 2

Place: Pune, Maharashtra

Location: 18°39'37.91"N, 73°51'17.35"E

Average Waste Height: 10.65 m

Area of Landfill: 97019.16 m^2

Estimated Volume of Waste: 1033601.67 m^3

Total Pixel Population within AOI (N): 116

Sentinel-1 Image Dates: 02-Jan-2025 and 26-Jan-2025

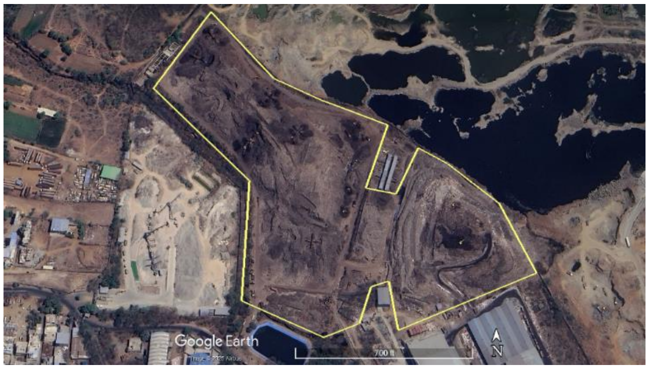

Fig. 184 Google Earth Imagery

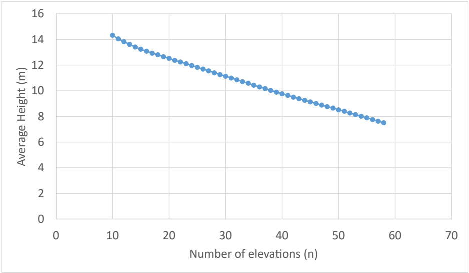

Fig. 185 Waste height (m) variation with change in pixel population (n)

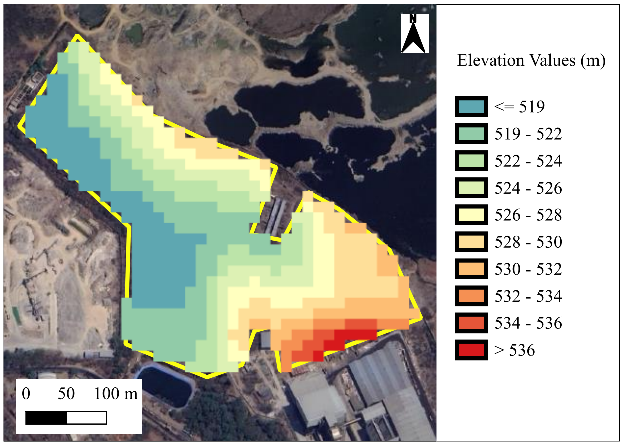

Fig. 186 Landfill elevation variations

Landfill Site 63: Punjab Zirakpur Landfill

Place: Zirakpur, Punjab

Location: 30°38'46.60"N, 76°49'59.76"E

Average Waste Height: 2.21 m

Area of Landfill: 59939.73 m^2

Estimated Volume of Waste: 132324.76 m^3

Total Pixel Population within AOI (N): 79

Sentinel-1 Image Dates: 02-Jan-2025 and 26-Jan-2025

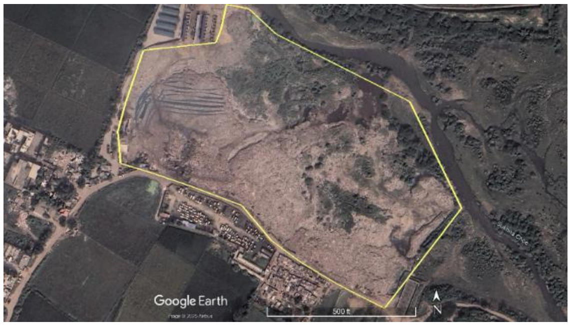

Fig. 187 Google Earth Imagery

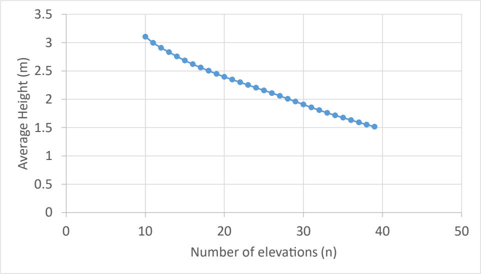

Fig. 188 Waste height (m) variation with change in pixel population (n)

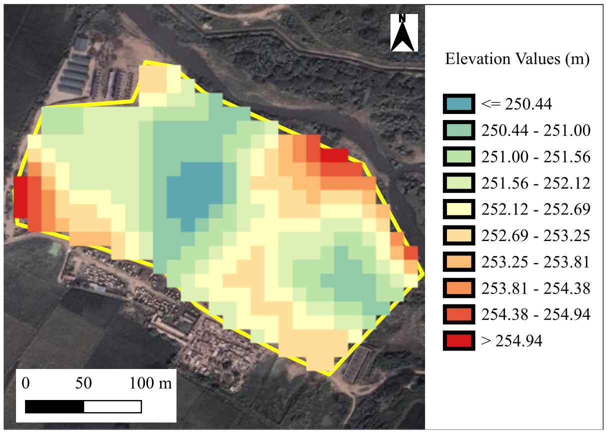

Fig. 189 Landfill elevation variations

Landfill Site 64: Rajgarh Landfill

Place: Rajgarh, Rajasthan

Location: 28°37'20.26"N, 75°23'57.77"E

Average Waste Height: 3.37 m

Area of Landfill: 47976.72 m^2

Estimated Volume of Waste: 161554.75 m^3

Total Pixel Population within AOI (N): 61

Sentinel-1 Image Dates: 02-Jan-2025 and 26-Jan-2025

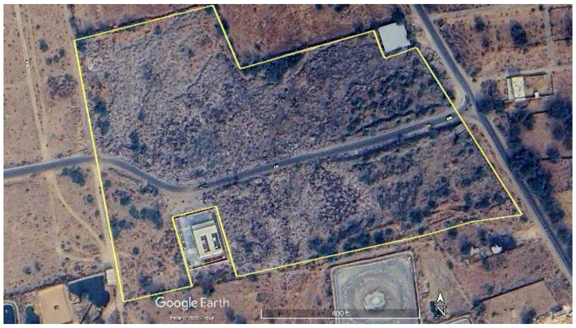

Fig. 190 Google Earth Imagery

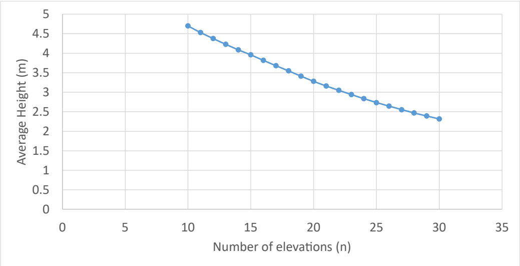

Fig. 191 Waste height (m) variation with change in pixel population (n)

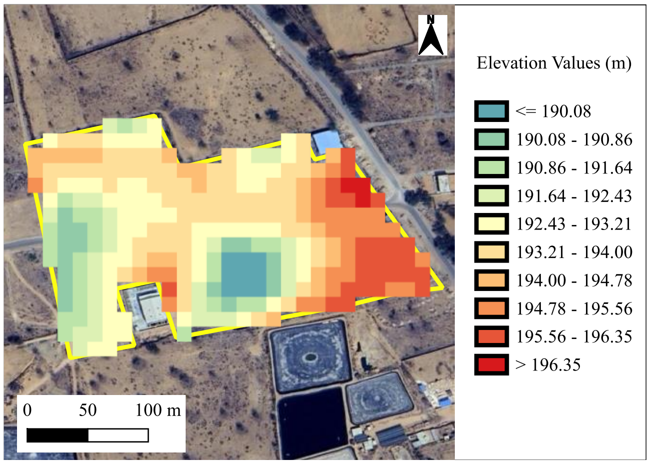

Fig. 192 Landfill elevation variations

Landfill Site 65: Ratlam Landfill

Place: Ratlam, Madhya Pradesh

Location: 23°21'5.35"N, 74°59'48.62"E

Average Waste Height: 3.83 m

Area of Landfill: 41520.73 m^2

Estimated Volume of Waste: 158890.62 m^3

Total Pixel Population within AOI (N): 50

Sentinel-1 Image Dates: 02-Jan-2025 and 26-Jan-2025

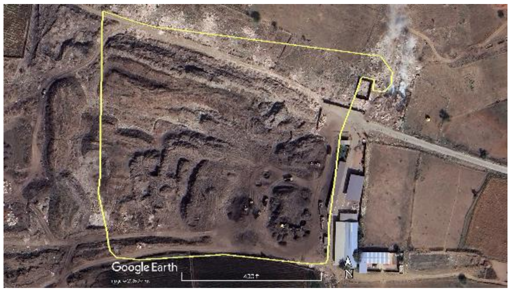

Fig. 193 Google Earth Imagery

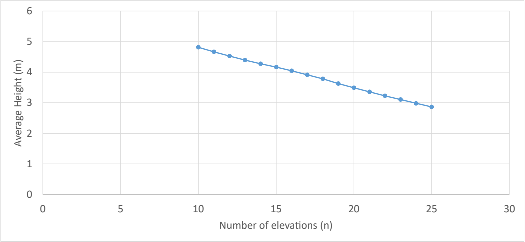

Fig. 194 Waste height (m) variation with change in pixel population (n)

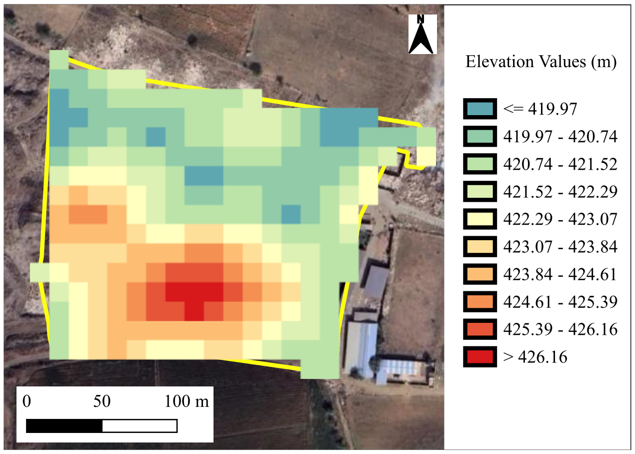

Fig. 195 Landfill elevation variations

Landfill Site 66: Sahibzada Ajit Singh Nagar Landfill

Place: Sahibzada Ajit Singh Nagar, Punjab

Location: 30°42'36.25"N, 76°40'59.28"E

Average Waste Height: 2.86 m

Area of Landfill: 47944.54 m^2

Estimated Volume of Waste: 137329.68 m^3

Total Pixel Population within AOI (N): 64

Sentinel-1 Image Dates: 02-Jan-2025 and 26-Jan-2025

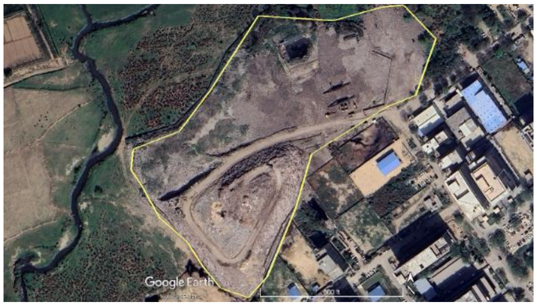

Fig. 196 Google Earth Imagery

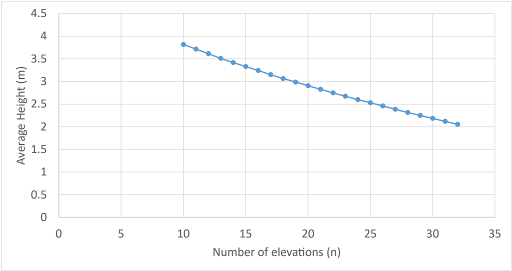

Fig. 197 Waste height (m) variation with change in pixel population (n)

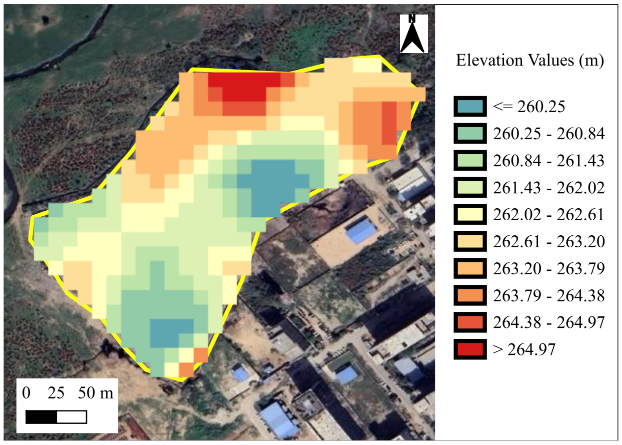

Fig. 198 Landfill elevation variations

Landfill Site 67: Satana Landfill

Place: Satana, Maharashtra

Location: 20°35'52.14"N, 74°13'5.87"E

Average Waste Height: 2.54 m

Area of Landfill: 21939.49 m^2

Estimated Volume of Waste: 55784.77 m^3

Total Pixel Population within AOI (N): 27

Sentinel-1 Image Dates: 02-Jan-2025 and 26-Jan-2025

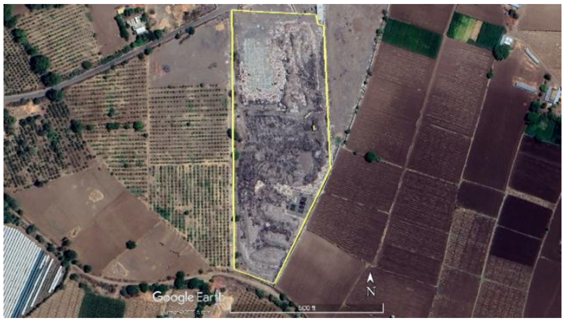

Fig. 199 Google Earth Imagery

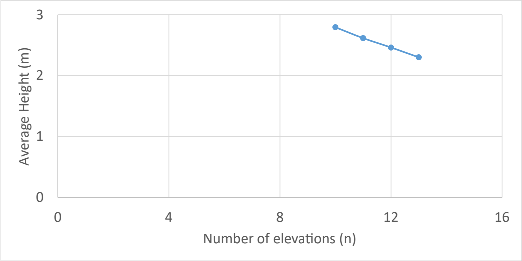

Fig. 200 Waste height (m) variation with change in pixel population (n)

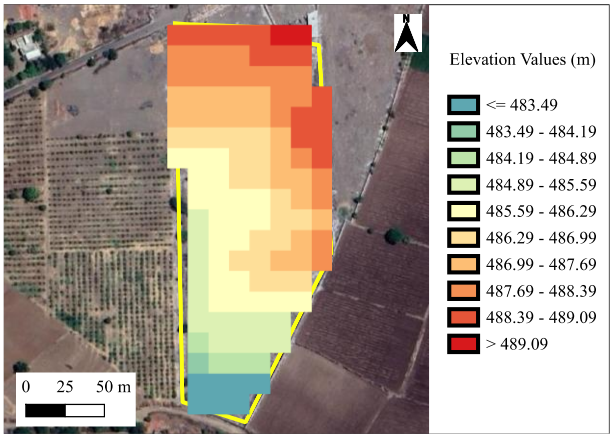

Fig. 201 Landfill elevation variations

Landfill Site 68: Siliguri Landfill

Place: Siliguri, West Bengal

Location: 26°44'47.99"N, 88°27'5.03"E

Average Waste Height: 2.71 m

Area of Landfill: 60561.34 m^2

Estimated Volume of Waste: 163855.42 m^3

Total Pixel Population within AOI (N): 76

Sentinel-1 Image Dates: 08-Jan-2025 and 20-Jan-2025

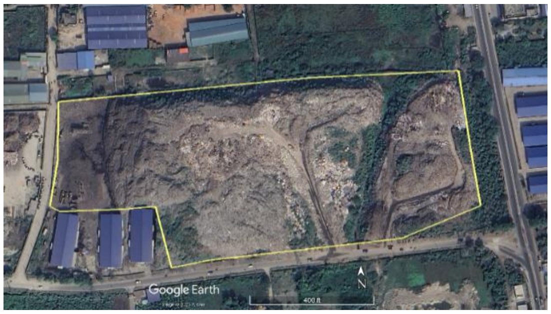

Fig. 202 Google Earth Imagery

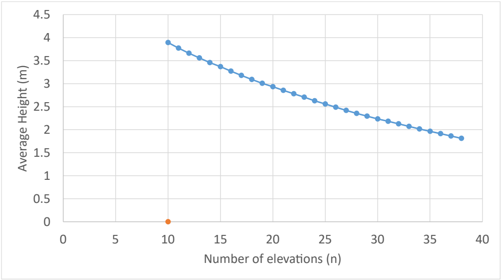

Fig. 203 Waste height (m) variation with change in pixel population (n)

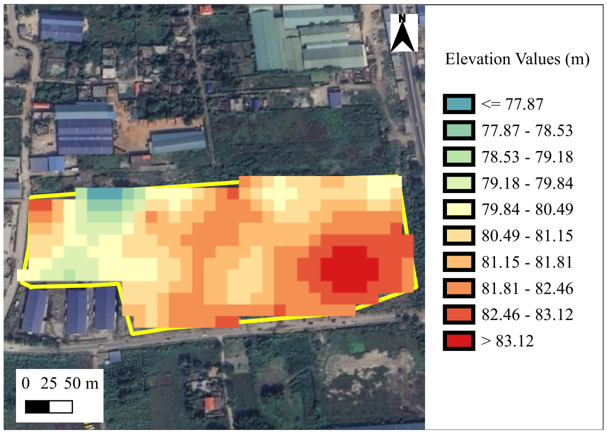

Fig. 204 Landfill elevation variations

Landfill Site 69: Tamil Nadu Chennai Dumping Ground 02

Place: Chengalpattu, Tamil Nadu

Location: 12°41'25.95"N, 79°57'54.75"E

Average Waste Height: 0.31 m

Area of Landfill: 14185.18 m^2

Estimated Volume of Waste: 4348.18 m^3

Total Pixel Population within AOI (N): 15

Sentinel-1 Image Dates: 11-Jan-2025 and 23-Jan-2025

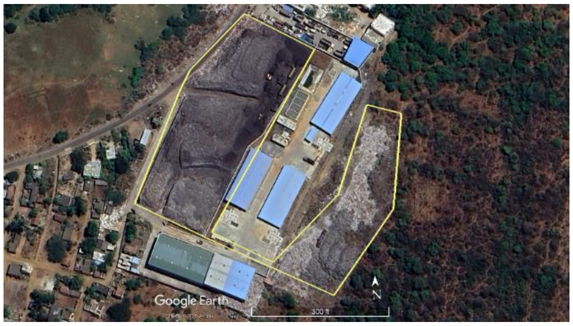

Fig. 205 Google Earth Imagery

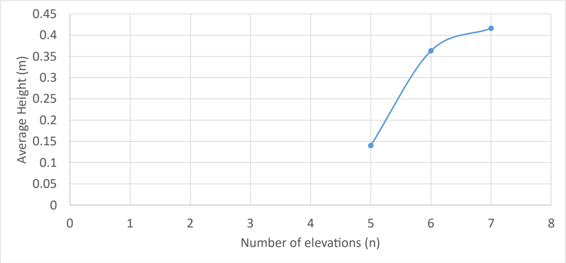

Fig. 206 Waste height (m) variation with change in pixel population (n)

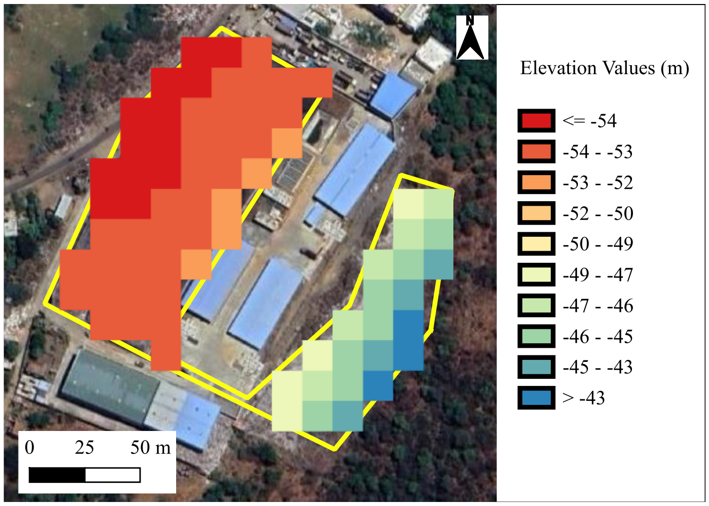

Fig. 207 Landfill elevation variations

Landfill Site 70: Tamil Nadu Tambaram Dumping Ground

Place: Tambaram, Tamil Nadu

Location: 12°56'4.22"N, 80° 6'2.19"E

Average Waste Height: 2.18 m

Area of Landfill: 17929.77 m^2

Estimated Volume of Waste: 39150.97 m^3

Total Pixel Population within AOI (N): 19

Sentinel-1 Image Dates: 11-Jan-2025 and 23-Jan-2025

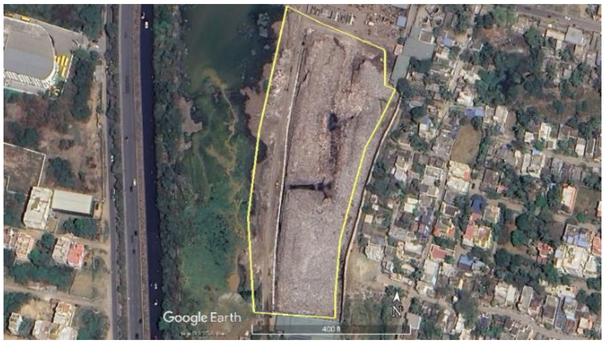

Fig. 208 Google Earth Imagery

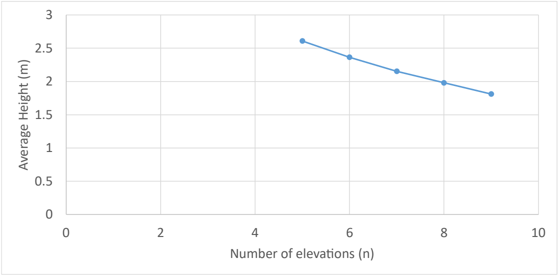

Fig. 209 Waste height (m) variation with change in pixel population (n)

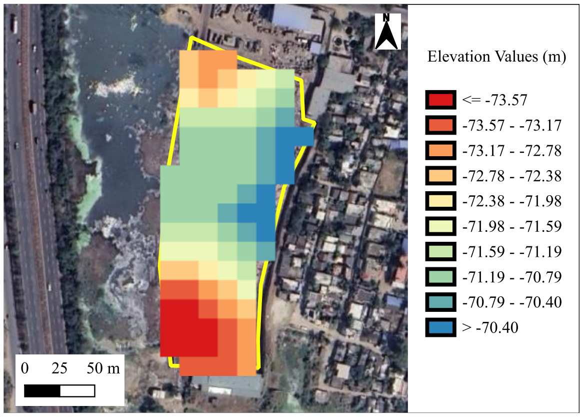

Fig. 210 Landfill elevation variations

Landfill Site 71: Telangana Madikonda Landfill

Place: Warangal, Telangana

Location: 17°57'10.07"N, 79°27'11.80"E

Average Waste Height: 2.97 m

Area of Landfill: 122171.78 m^2

Estimated Volume of Waste: 363238.53 m^3

Total Pixel Population within AOI (N): 143

Sentinel-1 Image Dates: 04-Jan-2025 and 28-Jan-2025

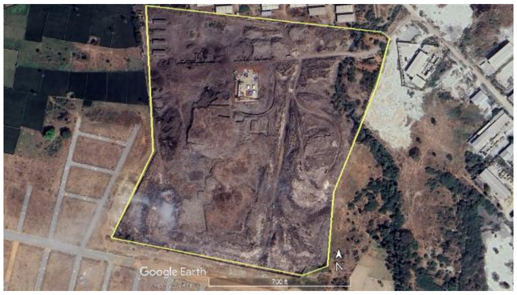

Fig. 211 Google Earth Imagery

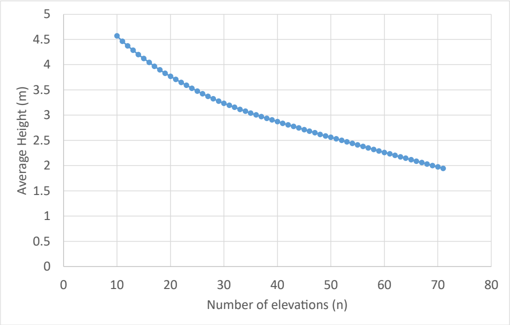

Fig. 212 Waste height (m) variation with change in pixel population (n)

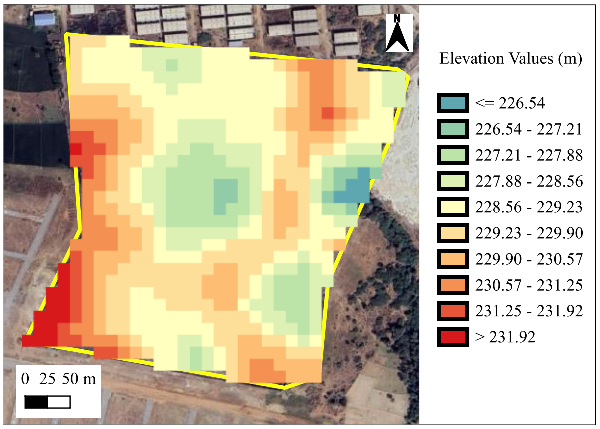

Fig. 213 Landfill elevation variations

Landfill Site 72: Titagarh Landfill

Place: Titagarh, West Bengal

Location: 22°44'56.43"N, 88°22'58.19"E

Average Waste Height: 2.48 m

Area of Landfill: 18972.57 m^2

Estimated Volume of Waste: 47106.79 m^3

Total Pixel Population within AOI (N): 22

Sentinel-1 Image Dates: 08-Jan-2025 and 20-Jan-2025

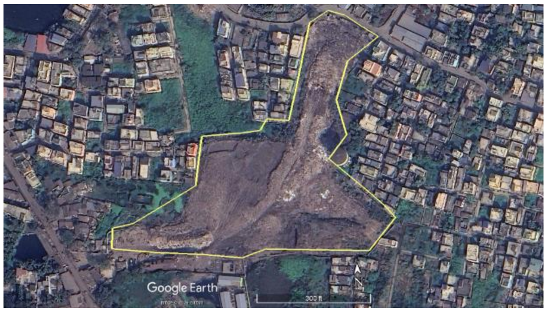

Fig. 214 Google Earth Imagery

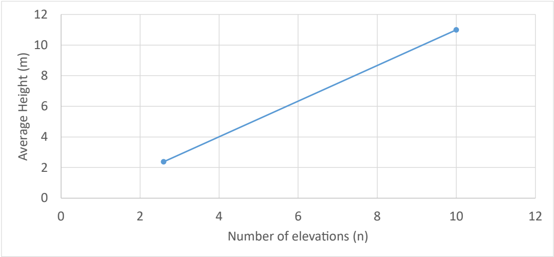

Fig. 215 Waste height (m) variation with change in pixel population (n)

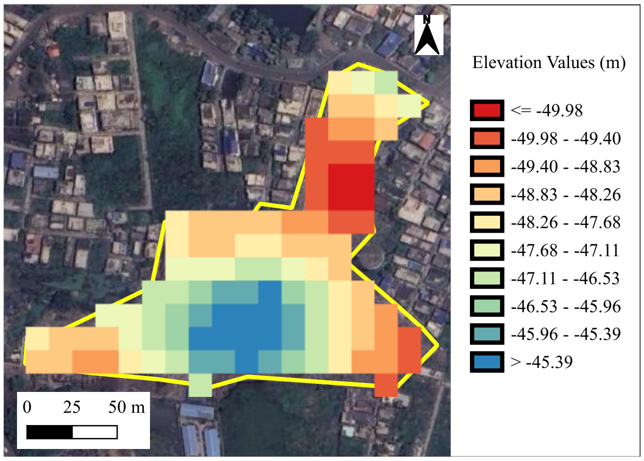

Fig. 216 Landfill elevation variations

Landfill Site 73: Trenching ground Indore

Place: Indore, Madhya Pradesh

Location: 22°40'21.84"N, 75°55'31.31"E

Average Waste Height: 10.95 m

Area of Landfill: 131095.66 m^2

Estimated Volume of Waste: 1435735.02 m^3

Total Pixel Population within AOI (N): 155

Sentinel-1 Image Dates: 02-Jan-2025 and 26-Jan-2025

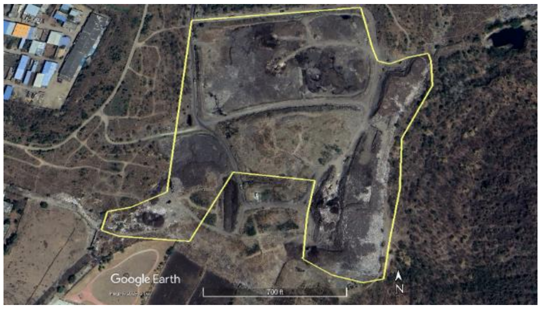

Fig. 217 Google Earth Imagery

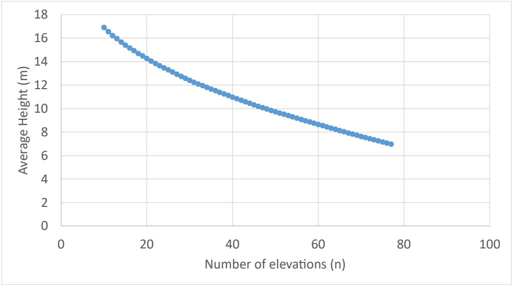

Fig. 218 Waste height (m) variation with change in pixel population (n)

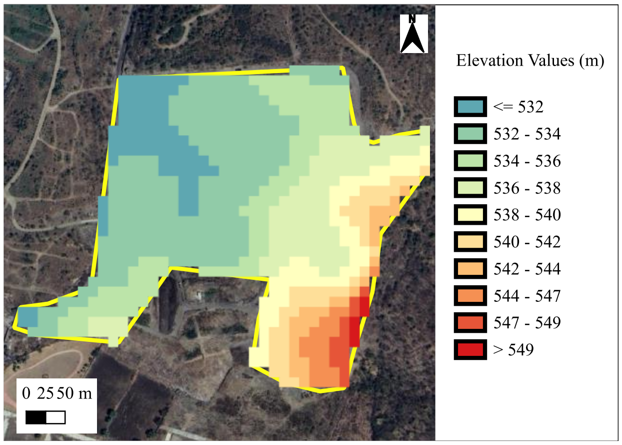

Fig. 219 Landfill elevation variations

Landfill Site 74: Tripura Debendranagar Landfill

Place: Agartala, Tripura

Location: 23°53'10.67"N, 91°19'20.48"E

Average Waste Height: 3.72 m

Area of Landfill: 51675.27 m^2

Estimated Volume of Waste: 192136.86 m^3

Total Pixel Population within AOI (N): 61

Sentinel-1 Image Dates: 09-Jan-2025 and 21-Jan-2025

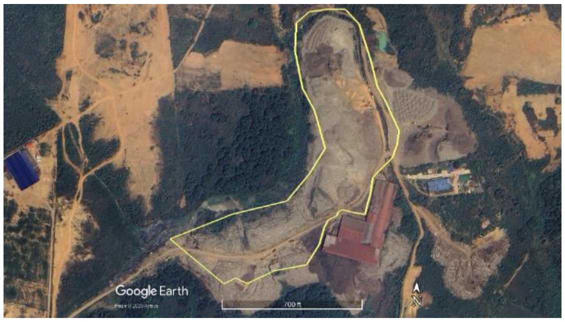

Fig. 220 Google Earth Imagery

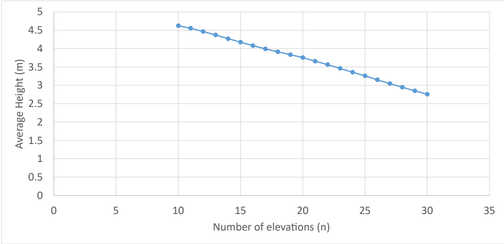

Fig. 221 Waste height (m) variation with change in pixel population (n)

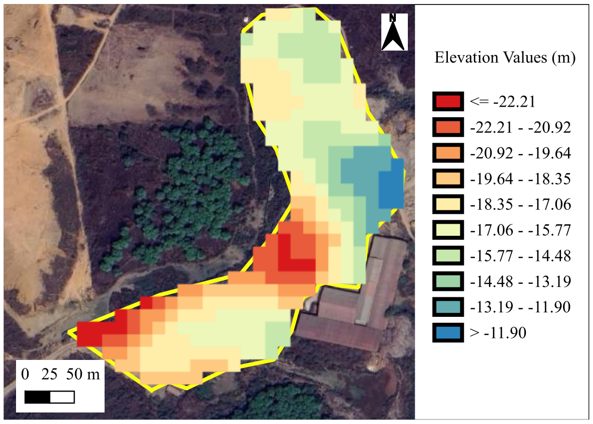

Fig. 222 Landfill elevation variations

Landfill Site 75: UK_Dehradun_Landfill

Place: Dehradun, Uttarakhand

Location: 30°20'36.61"N, 77°52'5.23"E

Average Waste Height: 2.20 m

Area of Landfill: 46495.50 m^2

Estimated Volume of Waste: 102227.88 m^3

Total Pixel Population within AOI (N): 62

Sentinel-1 Image Dates: 02-Jan-2025 and 26-Jan-2025

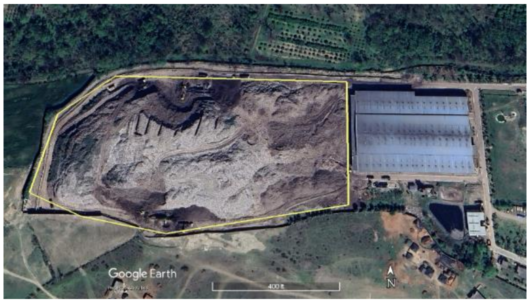

Fig. 223 Google Earth Imagery

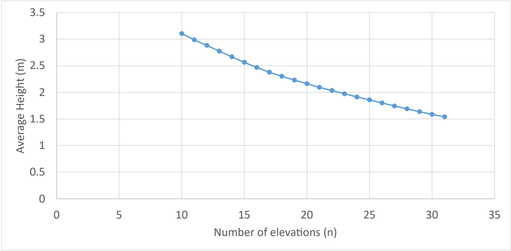

Fig. 224 Waste height (m) variation with change in pixel population (n)

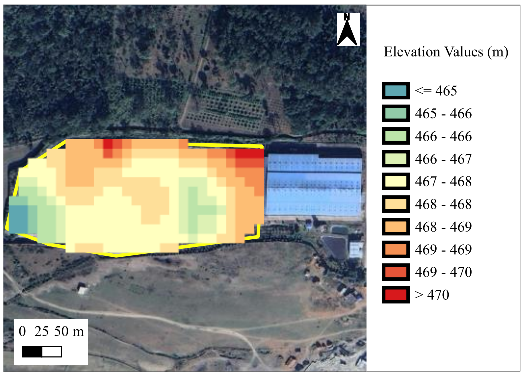

Fig. 225 Landfill elevation variations

Landfill Site 76: UK_Dehradun_Landfill_2

Place: Dehradun, Uttarakhand

Location: 30°21'33.62"N, 78° 0'46.56"E

Average Waste Height: 1.74 m

Area of Landfill: 21438.12 m^2

Estimated Volume of Waste: 37317.61 m^3

Total Pixel Population within AOI (N): 27

Sentinel-1 Image Dates: 02-Jan-2025 and 26-Jan-2025

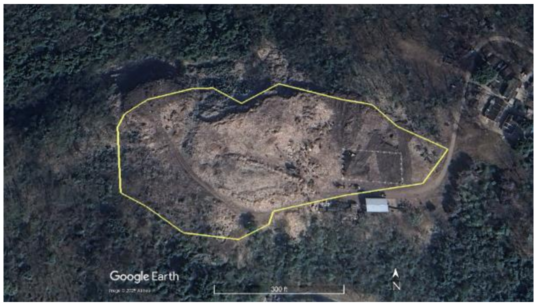

Fig. 226 Google Earth Imagery

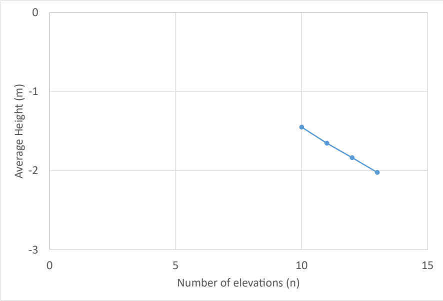

Fig. 227 Waste height (m) variation with change in pixel population (n)

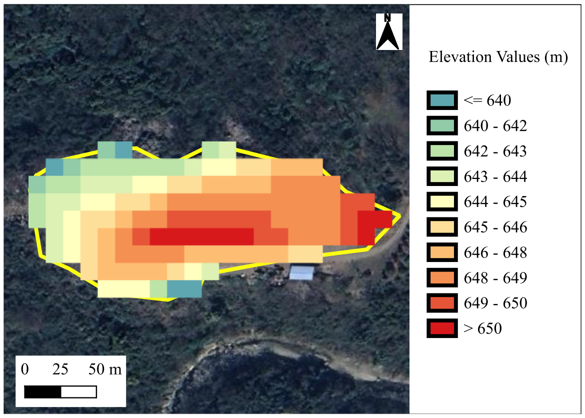

Fig. 228 Landfill elevation variations

Landfill Site 77: Vadodra_Atladara_Landfill

Place: Vadodara, Gujarat

Location: 22°16'42.07"N, 73° 9'59.75"E

Average Waste Height: 2.86 m

Area of Landfill: 23581.91 m^2

Estimated Volume of Waste: 67538.53 m^3

Total Pixel Population within AOI (N): 28

Sentinel-1 Image Dates: 07-Jan-2025 and 31-Jan-2025

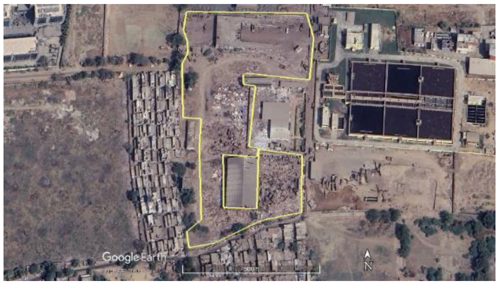

Fig. 229 Google Earth Imagery

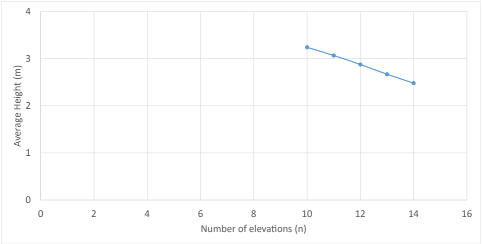

Fig. 230 Waste height (m) variation with change in pixel population (n)

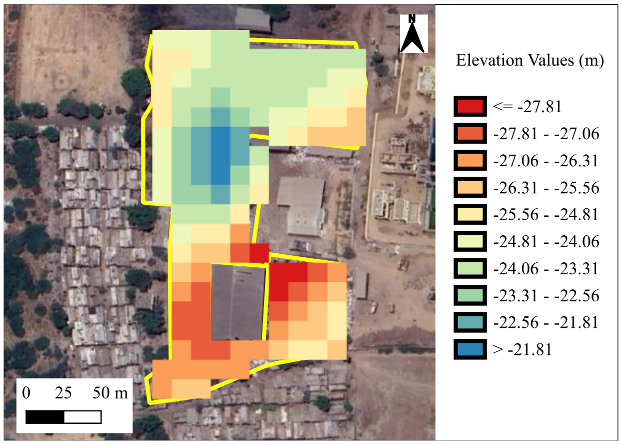

Fig. 231 Landfill elevation variations

Landfill Site 78: Varanasi_Near_IIT_BHU_Landfill

Place: Varanasi, Uttar Pradesh

Location: 25°15'27.40"N, 82°59'40.60"E

Average Waste Height: 1.37 m

Area of Landfill: 10381.92 m^2

Estimated Volume of Waste: 14223.99 m^3

Total Pixel Population within AOI (N): 13

Sentinel-1 Image Dates: 11-Jan-2025 and 23-Jan-2025

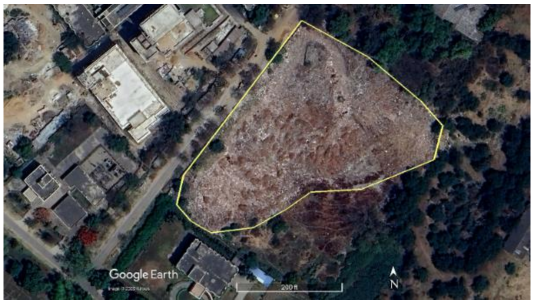

Fig. 232 Google Earth Imagery

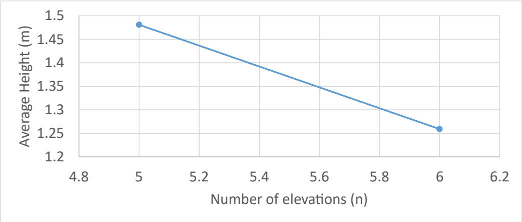

Fig. 233 Waste height (m) variation with change in pixel population (n)

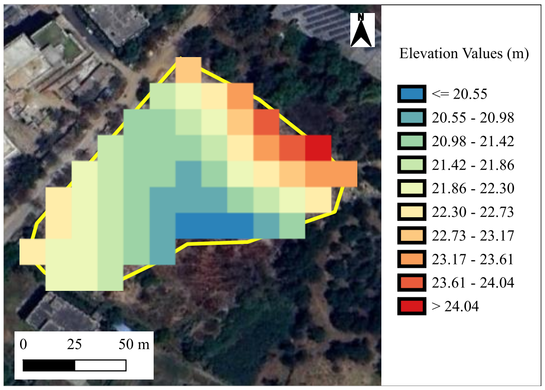

Fig. 234 Landfill elevation variations
